# Architecture of the 8 MDa Hdr–Vhu–Fwd super-assembly in class I methanogens

**DOI:** 10.1038/s41586-026-10744-9

**Published:** 2026-07-08

**Authors:** Sophia Paul, Tomas C. Pascoa, Max A. Klamke, Stefan Bohn, Frank Abendroth, Darja Deobald, Olalla Vázquez, Sven T. Stripp, Jan M. Schuller

**Affiliations:** 1 Department of Chemistry and Center for Synthetic Microbiology (SYNMIKRO), Philipps-Universität Marburg, Marburg, Germany; 2 Spectroscopy & Biocatalysis, Institute of Chemistry, University of Potsdam, Potsdam, Germany; 3 Helmholtz Munich Cryo-Electron Microscopy Platform, Helmholtz Munich, Neuherberg, Germany; 4 Department of Environmental Biotechnology, Helmholtz Centre for Environmental Research (UFZ), Leipzig, Germany; 5 Microbes-for-Climate (M4C) Cluster of Excellence, Marburg, Germany

**Keywords:** Cryoelectron microscopy, Bioenergetics, Archaeal biology

## Abstract

Methanogens are central to global carbon cycling and among the largest biological sources of methane, a potent greenhouse gas^1^. At the heart of their energy metabolism lies the Hdr–Vhu–Fwd super-assembly, which couples H_2_ oxidation with CO_2_ reduction through flavin-based electron bifurcation. Here we present the cryogenic electron microscopy structure of the Hdr–Vhu–Fwd super-assembly from *Methanococcus maripaludis*, revealing an 8 MDa complex comprising 252 polypeptide chains and over 600 redox cofactors. Cryo-electron tomography further support that this super-assembly forms an intact structure within the cytoplasm of intact cells. This architecture comprises two hexameric HdrABC–Vhu rings linked by a tetrameric FwdF core, forming a continuous, circular electron chain. In this unique arrangement, 12 polyferredoxin subunits (VhuB) connect the Vhu–Hdr and Fwd complexes, thereby coupling electron bifurcation with CO_2_ reduction and directly linking the last and the first step of methanogenesis. Moreover, we identify a modular variant of the complex in which the [NiFe]-hydrogenase Vhu is substituted by tungsten-containing formate dehydrogenase (FdhAB), indicating flexible integration of electron-input modules facilitating metabolic adaptation under diverse environmental conditions^2^. Analysis of the taxonomic distribution reveals that this architecture is specific to class I methanogens and is distinct from the smaller Hdr–Fmd complex of class II^3^. Together, our study reveals that the the Hdr–Vhu–Fwd super-assembly has a modular and adaptable bioenergetic assembly, suggesting a lineage-specific architecture to adapt to diverse anaerobic niches.

## Abstract (web summary)

The structure of the Hdr–Vhu–Fwd super-assembly reveals a lineage-specific and modular 8 MDa complex with the ability to adapt to diverse anaerobic niches.

## Main

Methanogenic archaea substantially impact the global climate by producing the majority of all biogenic methane (CH_4_)^1,4^, yet they also offer a valuable source of renewable energy. Understanding the biochemical pathways of microbial CH_4_ formation is therefore essential for climate change mitigation, the development of synthetic biology and biotechnological applications. In hydrogenotrophic methanogenesis, electrons from donors such as hydrogen (H_2_) and formate are used to reduce carbon dioxide (CO_2_) through a series of C_1_ carriers, ultimately producing CH_4_ and the heterodisulfide CoM-S-S-CoB in the last CH_4_-forming reaction^5,6^. This provides a direct mechanistic link between the final and initial steps of methanogenesis, shaping a continuous cycle termed the Wolfe cycle^7^.

This linkage is realized through a super-assembly in which the heterodisulfide reductase (HdrABC) directly interacts with the tungsten-containing CO_2_-reducing formyl-methanofuran dehydrogenase (FwdABCDFG) or its molybdenum isoform (Fmd)^2^. These enzymes are powered with either a [NiFe]-hydrogenase (VhuADGU) or a formate dehydrogenase (FdhAB), connecting the exergonic reduction of CoM-S-S-CoB (standard reduction potential *E*^0^′ = −281 mV)^8^ with the endergonic reduction of polyferredoxin subunits (approximately *E*^0^′ = −500 mV)^9^ through flavin-based electron bifurcation (FBEB)^10^. In this process, the energy of an electron pair can be distributed asymmetrically, giving rise to a low-potential electron for CO_2_ reduction and a high-potential electron for the reduction of CoM-S-S-CoB to CoM-SH and CoB-SH. The polyferredoxins VhuB and FwdF transfer the low-potential electrons to the active site of FwdBC, where they are used to reduce CO_2_ to formate, which is then condensed to the C_1_-carrier methanofuran to form formyl-methanofuran^11,12^. This intermediate is then transferred to tetrahydromethanopterin (H_4_MPT) and stepwise reduced along the methyl branch of the Wood–Ljungdahl pathway to methyl- H_4_MPT.

Currently, our understanding of these super-assemblies is based on a single structure from *Methanospirillum hungatei*^3,13^. The complex revealed a hexameric architecture that directly connects the CO_2_-reducing formyl-methanofuran dehydrogenase (Fmd)^12^ with the heterodisulfide reductase (HdrABC)^14^ through FmdF, supporting direct transfer of low-potential electrons within the complex. Despite its initial consideration as broadly representative, it remains uncertain how well this structure reflects the diversity of methanogenic species. This is underscored by the deep phylogenetic division of methanogenic archaea into two major clades: class I (such as *Methanopyrus*, *Methanococcales* and *Methanobacteriales*) and class II (such as *Methanomicrobiales*, *Methanocellales* and *Methanosarcinales*) that differ in their evolutionary origin and physiology^15–17^. Whereas many genes are conserved among methanogenic archaea, distinct differences can be found between those that grow under different environmental conditions. Many class I methanogens exhibit hydrogenotrophic growth and thrive under high-temperature conditions, except for *Methanococcus*, which is reflected in their reduced genome sizes. Class II methanogens can grow on a broader substrate range and at lower temperatures, resulting in a larger genome and the ability to activate different metabolic pathways^18–20^. Biochemical and proteomic analyses suggest that class I species may rely on a fundamentally distinct architecture, whereby the polyferredoxin VhuB (also known as MvhB) bridges the Vhu–Hdr and Fwd complexes^2,21^. However, without structural information, the mechanistic basis for efficient CO_2_ reduction and energy conservation across methanogenic diversity remains unresolved. Yet, this understanding is essential to decipher how different methanogen species persists under extreme, energy-limited conditions across different environmental conditions and stresses.

Here we reveal the structure of the Hdr–Vhu–Fwd super-assembly from the class I hydrogenotrophic methanogen *M. maripaludis*^22^. Our structure shows an 8 MDa super-assembly comprising 12 copies of the Hdr–Vhu dimer, which organize into two symmetric hexameric rings bridged by three tetrameric, hourglass-shaped Fwd(ABCDFG)_4_ complexes. In this unique arrangement, 12 polyferredoxin subunits (VhuB) link the Vhu–Hdr and Fwd complexes, thereby coupling FBEB with CO_2_ reduction and directly linking the last and the first step of methanogenesis. Functional analysis using in situ infrared spectroscopy corroborated electronic coupling between all catalytic subunits in the Hdr–Vhu–Fwd super-assembly. Conservation analysis of Hdr, Fwd/Fmd and VhuB components indicates that this architecture is representative of class I methanogens. Together, these findings highlight the structural divergence of Hdr super-assemblies of class I and class II methanogens and underscore the evolutionary diversification of methanogenic bioenergetic systems across archaeal lineages.

## Isolation of the Hdr super-assembly

**Extended Data Fig. 1:**
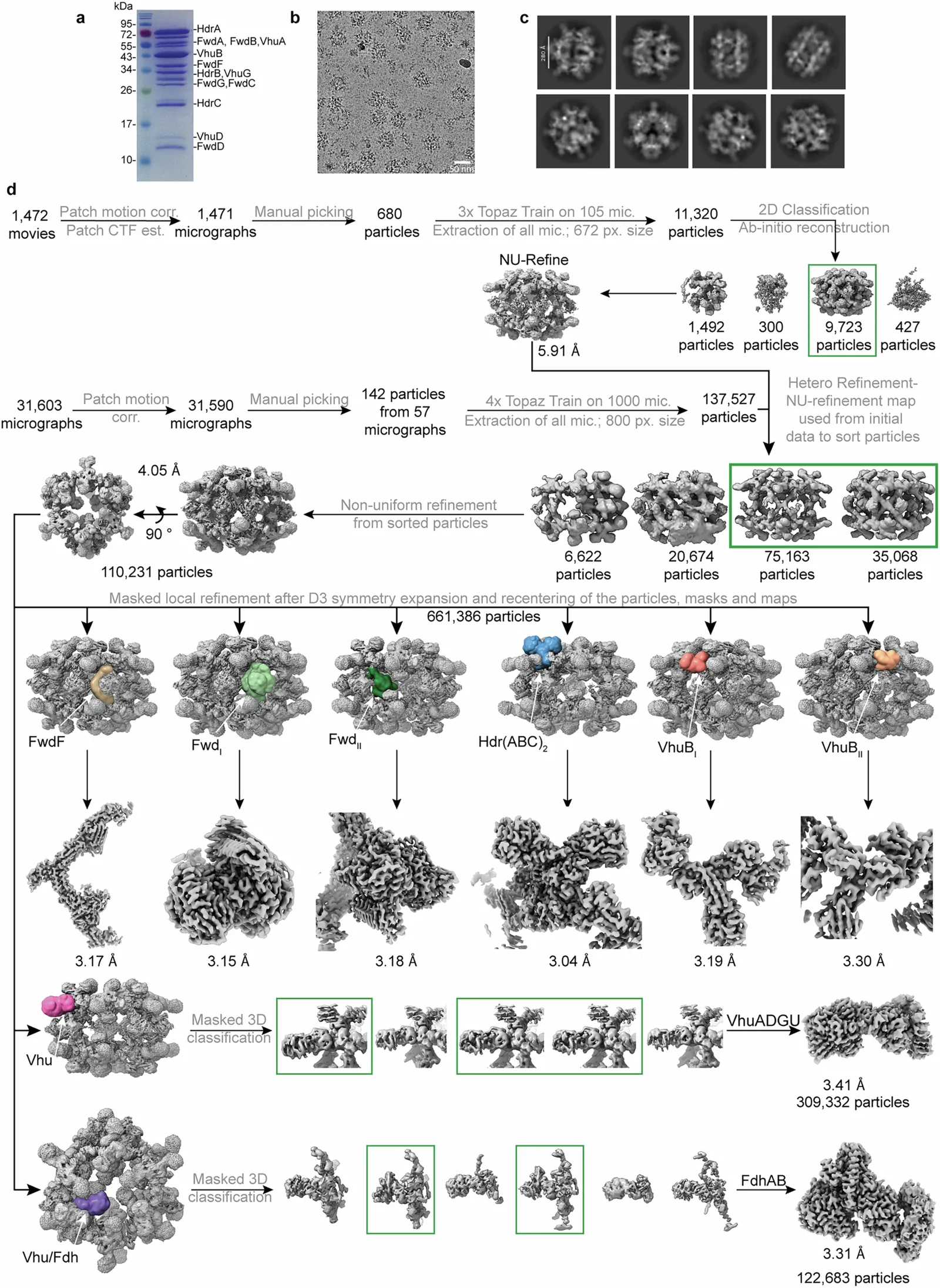
Cryo EM processing workflow. **a**, 15% SDS-PAGE of the purified protein sample with the individual subunits after anaerobic affinity chromatography. At least three independent SDS-PAGE analyses showed the same pattern. (For uncropped gel, see Supplementary Fig. 3). **b**, Representative micrograph of the super-assembly showing ring shaped particles. The same pattern and similar features were observed in numerous micrographs. **c**, 2D-classes after Topaz-training revealing two stacked rings forming the assembly. Scale bar, 50 nm. **d**, Processing tree showcasing the steps performed to determine the structure of the 8 MDa super-assembly. Moreover, local refinements were performed on the individual subunits and flexible regions to generate a composite map.

To purify the Hdr complex from the hydrogenotrophic class I methanogen *M. maripaludis*, we used the shuttle vector pLW40^23^ and transiently overexpressed the HdrB2 subunit with an N-terminal Twin-Strep tag in *M. maripaludis* JJ Δ*upt*^24^ (Extended Data Fig. 1a and Supplementary Tables 1 and 2). This enabled us to perform rapid single-step affinity chromatography, which assisted in preserving the fragile protein assembly. Using mass spectrometry (MS), we confirmed the presence of HdrABC, VhuADGU, FwdABCDFG and VhuB (Supplementary Table 3). Moreover, we identified an alternative electron-input module, the formate dehydrogenase FdhA2B2, suggesting a partial replacement of hydrogenases to preserve the architecture and function of the complex during stress conditions such as hydrogen limitation^2,25^.

## *M. maripaludis* Hdr is an 8 MDa assembly

**Fig. 1:**
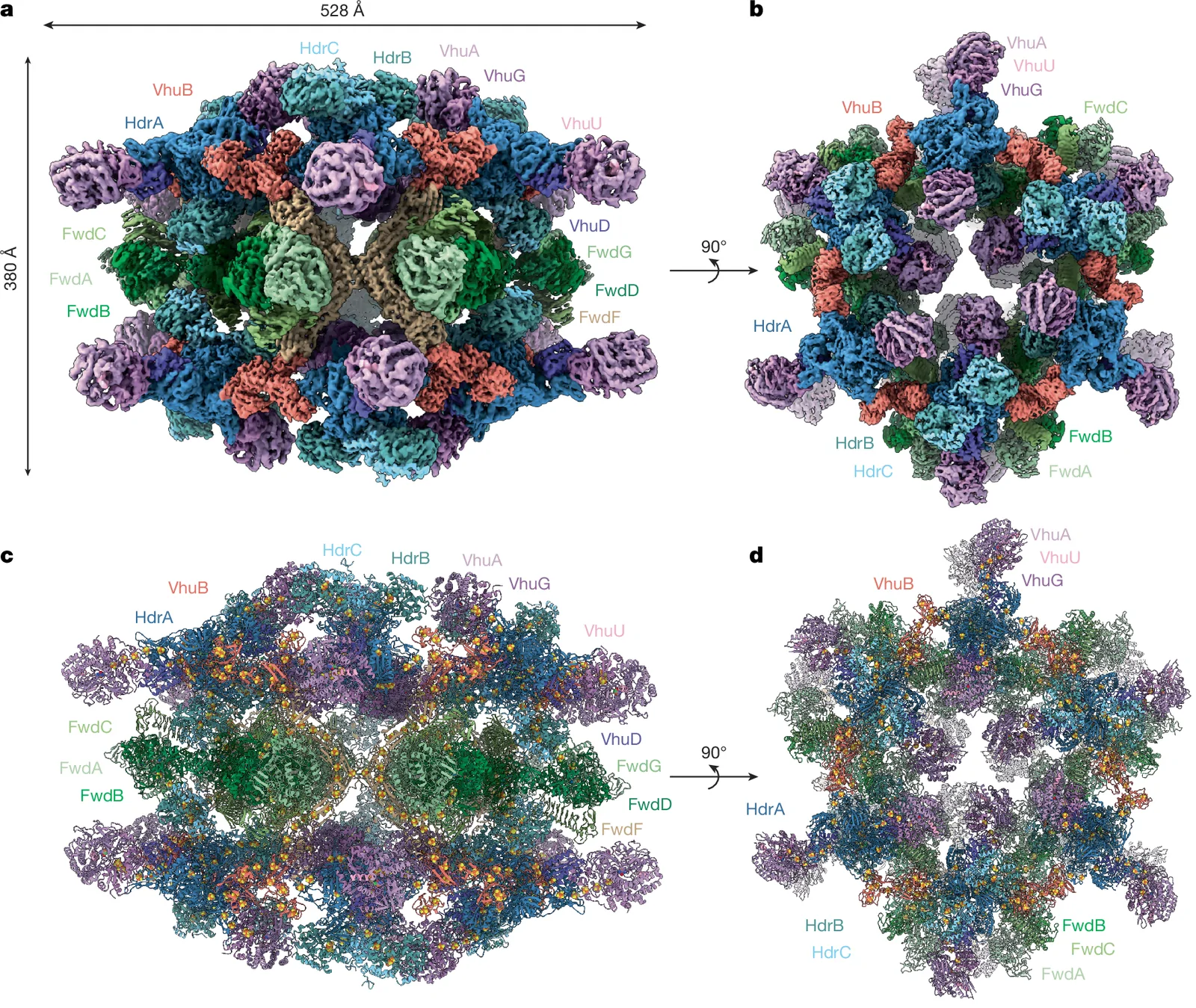
The overall architecture of the 8 MDa Hdr–Vhu–Fwd complex. **a**, Segmented and coloured composite cryo-EM map from the side (**a**) and top (**b**) view, with the corresponding protein model from the side (**c**) and top (**d**) view.

**Extended Data Fig. 2:**
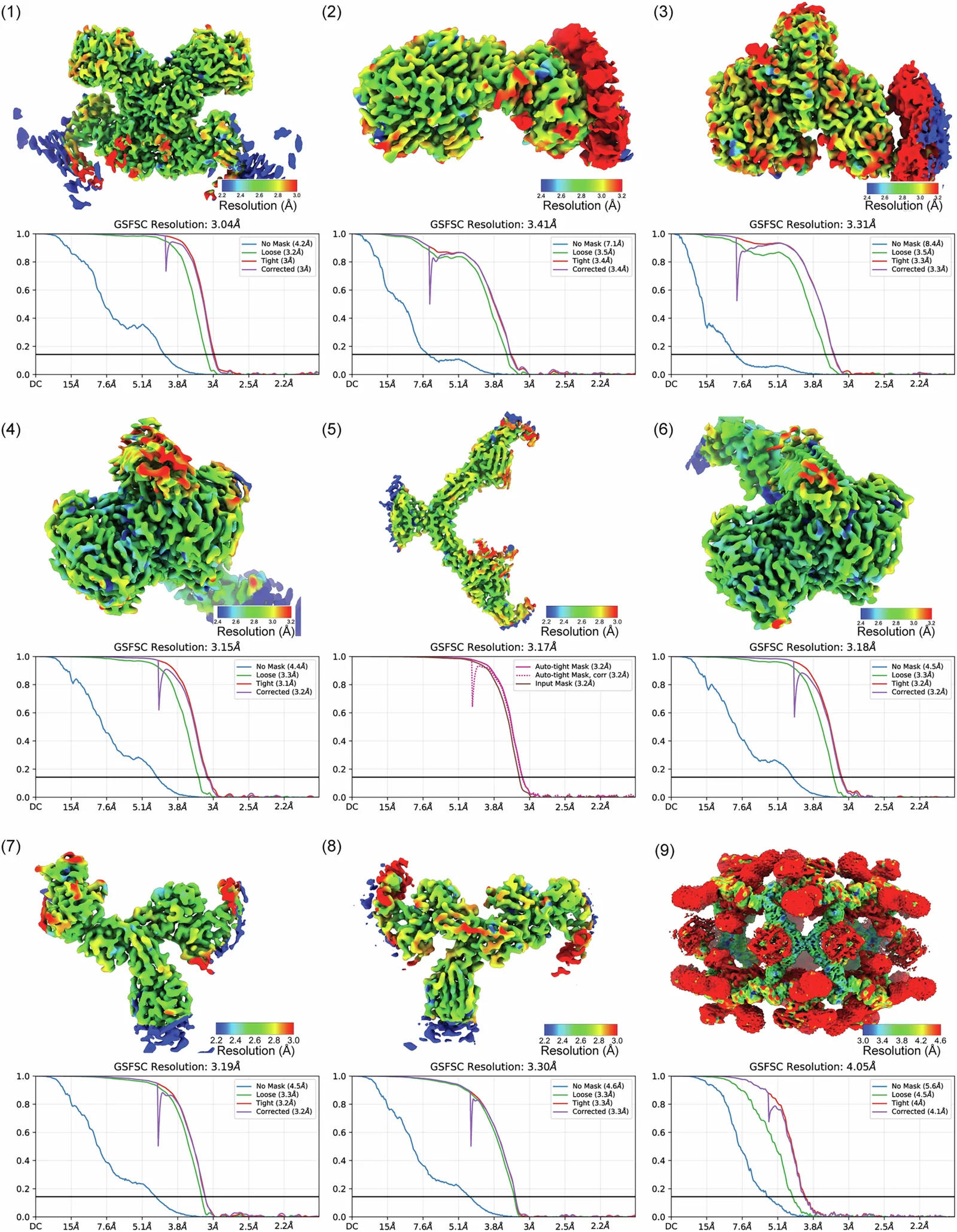
Cryo EM map resolution plots. Focused refined cryoEM maps coloured by their local resolution (up) calculated by the corresponding GSFSC plots (down). Displayed are the focus refined maps for Hdr(ABC)_2_ (1), the hydrogenase-arm VhuADGU (2), the formate dehydrogenase (3), the formyl-methanofuran dehydrogenase I (4) and II (6), as well as the FwdF- Dimer (5). Additionally, the polyferredoxin VhuB is represented in (7) and (8) alongside the consensus map (9) from the whole complex.

**Extended Data Fig. 3:**
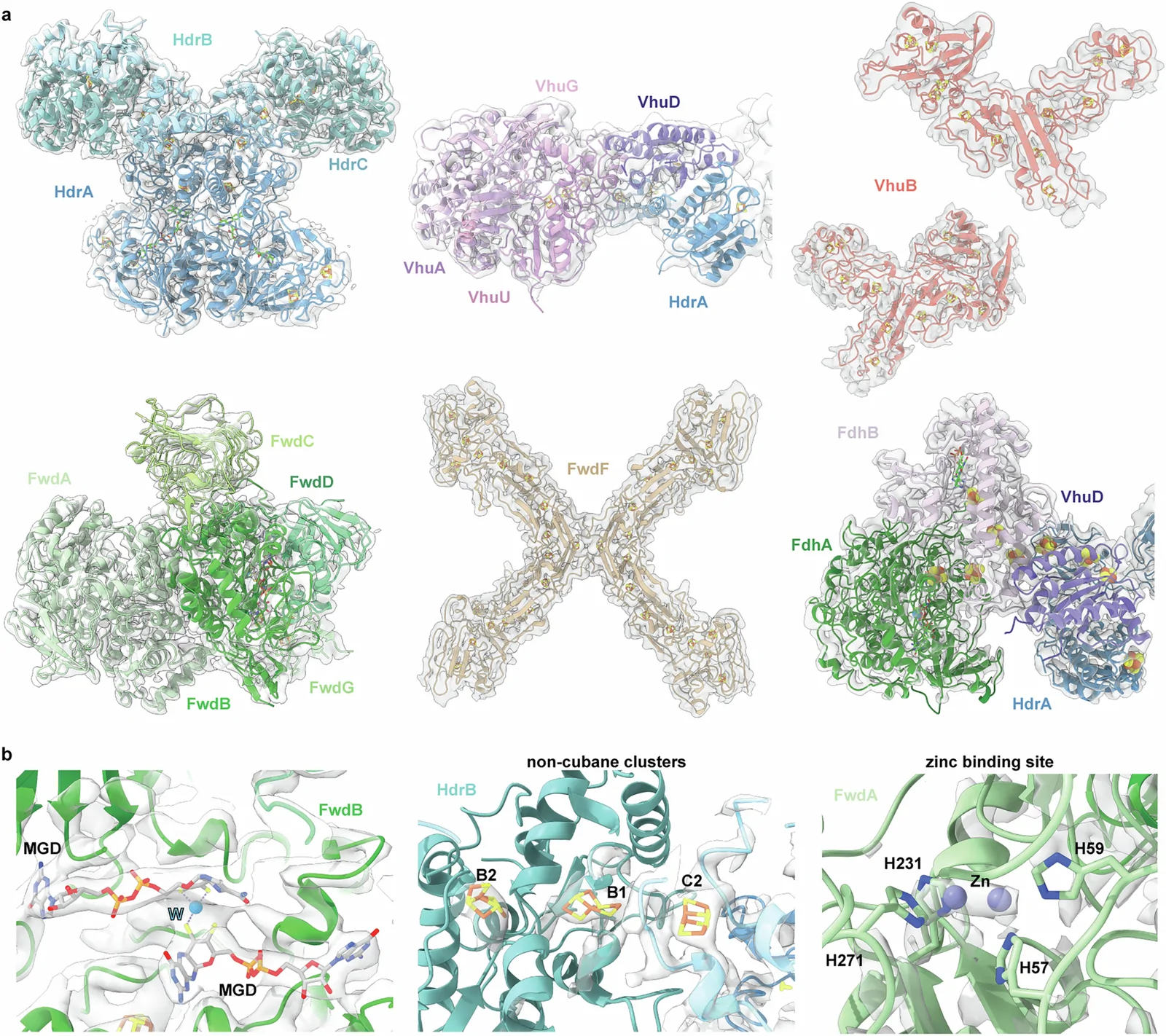
Cryo-EM density from local refinements with corresponding models. **a**, Models of the different subcomplexes with their surrounding cryo-EM density. **b**, Showing the active site for CO_2_-reduction (left), the non-cubane clusters of HdrB where CoM-S-S-CoB is reduced (middle) and the zinc-binding for the formyl-group transfer on methanofuran (right).

To identify the molecular architecture of the Hdr super-assembly, we performed redox-controlled cryogenic electron microscopy (cryo-EM) single-particle analysis (SPA) under anoxic conditions. Inspection of individual micrographs and reference-free 2D class averages revealed exclusively large ring-shaped particles with a diameter close to 50 nm (Extended Data Fig. 1b,c). After three-dimensional (3D) refinement, we obtained a 4-Å-resolution map of the ring-shaped complex, revealing an overall *D*_3_-symmetric organization (Fig. 1a,b and Extended Data Fig. 1d). To characterize the individual subcomplexes, we applied focused refinement on the HdrABC, VhuB, FwdF dimer and FwdABCDG. Notably, the hydrogenase (Vhu) subunits displayed considerable flexibility, prompting the use of 3D classification and focused refinement on these subunits (Extended Data Fig. 1d). This approach generated a series of local maps with resolutions ranging from 3.04 to 3.41 Å (Extended Data Fig. 2 and Extended Data Table 1), enabling us to model the entire super-assembly (Fig. 1c,d and Extended Data Fig. 3). Our structural model reveals that this large redox assembly contains 252 chains from 14 different proteins and over 690 cofactors: 546 [4Fe-4S], 24 [2Fe-2S], 48 non-cubane [4Fe-4S] clusters, 24 [NiFe] active-site cofactors, 24 flavin adenine dinucleotide (FAD) and 24 molybdopterin guanine dinucleotide (Supplementary Table 4).

## In situ cryo-ET confirms the assembly

**Fig. 2:**
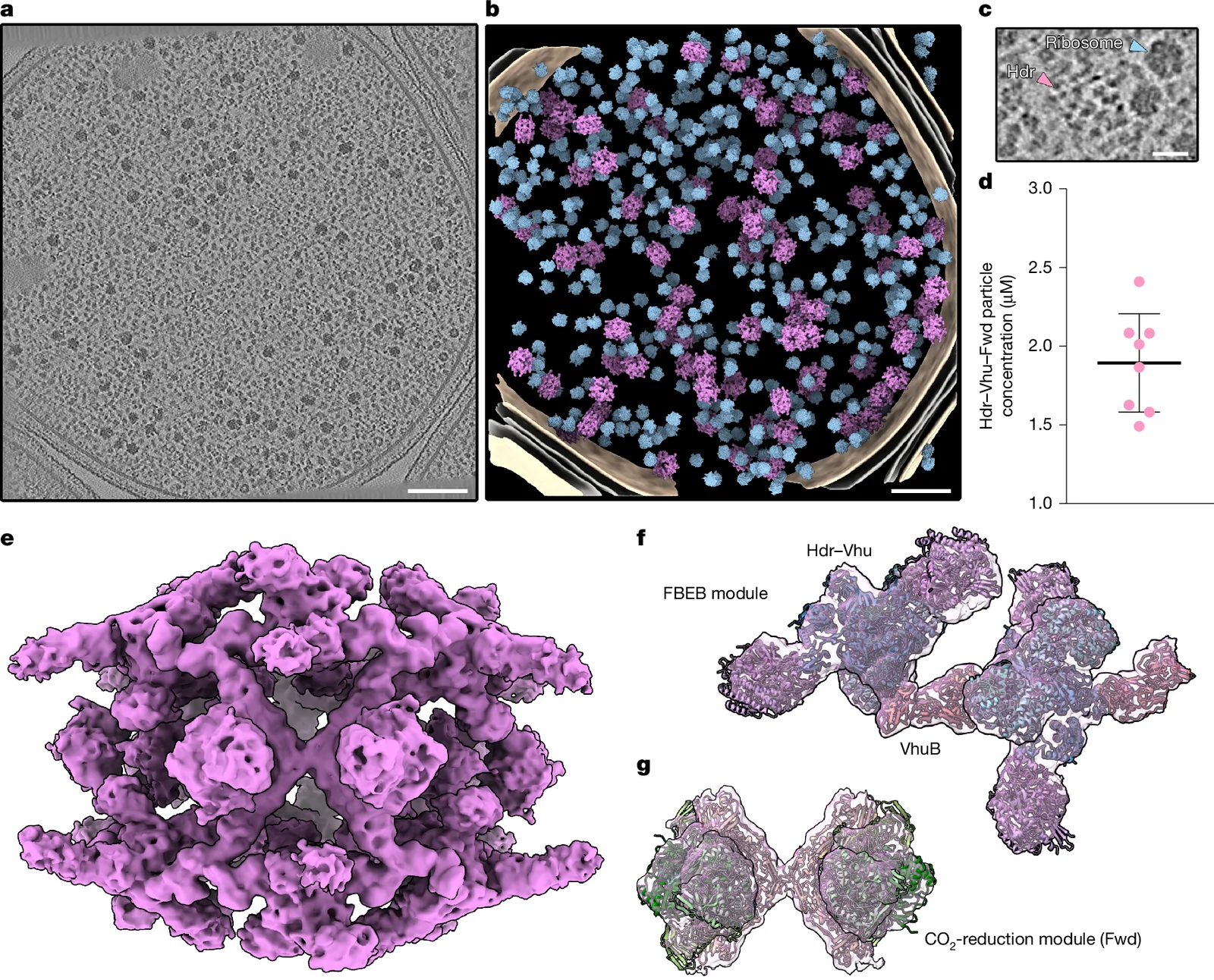
In situ visualization of the Hdr–Vhu–Fwd super-assembly in *M. maripaludis*. **a**, Slice through a representative *M. maripaludis* tomogram. Slice thickness, 7.72 Å. Scale bar, 100 nm. **b**, Segmentation and back-projection of subtomogram averages into the tomogram shown in **a**. The Hdr–Vhu–Fwd super-assembly is shown in pink and ribosomes are shown in blue; membrane and S-layer segmentations are shown in tan and grey, respectively. Scale bar, 100 nm. **c**, Magnified view of the location of a Hdr–Vhu–Fwd super-assembly. Scale bar, 20 nm. **d**, Cytoplasmic Hdr–Vhu–Fwd particle concentration calculated from a representative subset of eight tomograms. Data are mean ± s.d. *n* = 8 tomograms. **e**, The subtomogram average of the Hdr–Vhu–Fwd super-assembly. **f**,**g**, Fit of single-particle cryo-EM models of the Hdr–Vhu (**f**) and Fwd (**g**) subcomplexes into the subtomogram average. Source data

**Extended Data Fig. 4:**
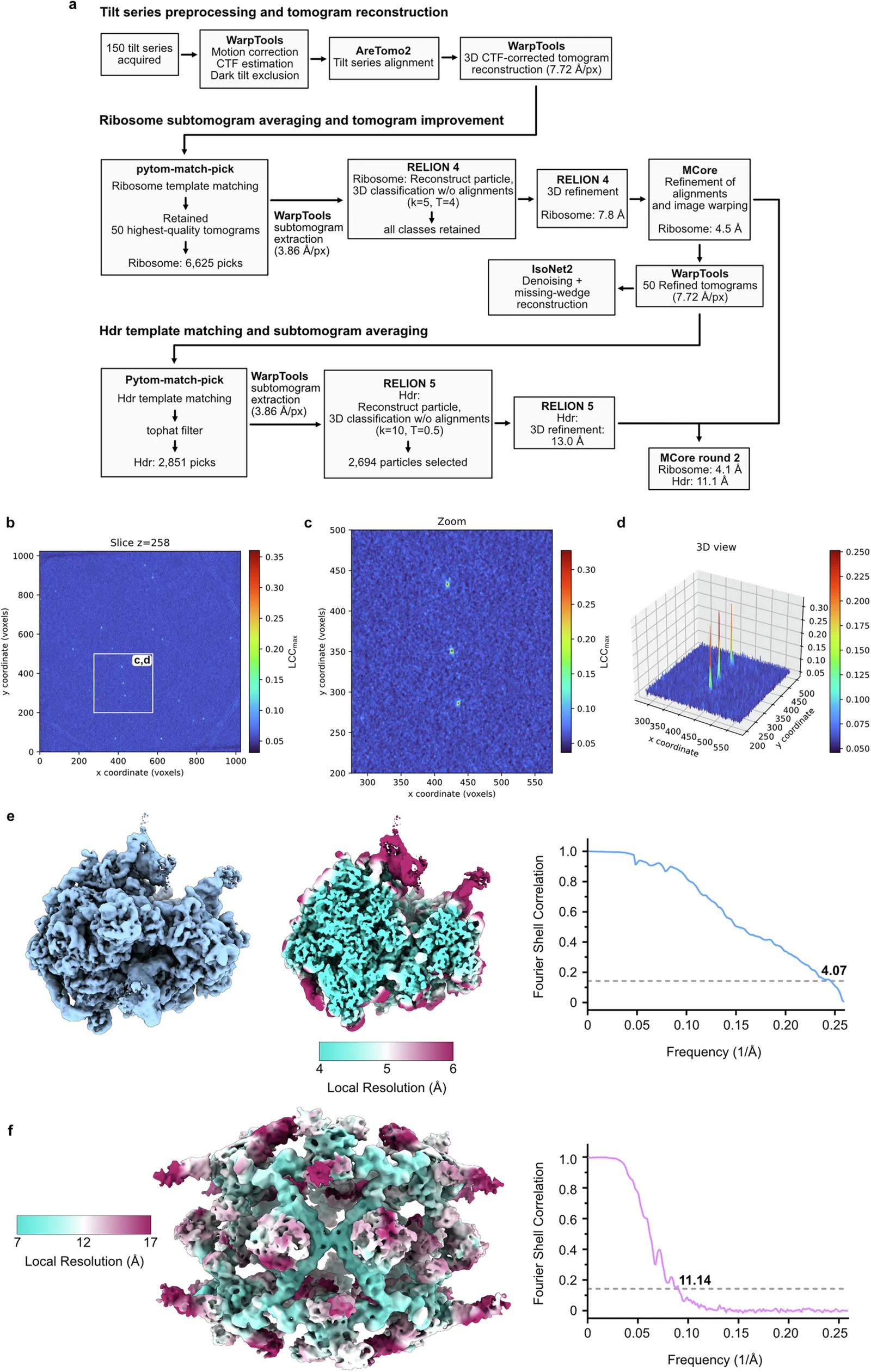
Cryo-ET processing workflow and subtomogram averaging map quality. **a**, Cryo-ET data processing workflow. Tilt series were first aligned and tomograms were reconstructed, followed by refinement of tilt series alignments using ribosomes as quasi-fiducial markers. The improved tomograms were then used for template matching of the Hdr super-assembly, which was followed by subtomogram averaging and a final round of multi-particle refinement in M. A detailed description is provided in Materials and Methods. **b**, Slice through a representative score map obtained by template matching of the Hdr-Vhu-Fwd super-assembly in the optimized tomograms. **c**, Enlarged 2D view of the score map shown in **b**, where three sharp peaks corresponding to three super-assemblies are observed. **d**, 3D view of the region of the score map shown in **c. e**, Ribosome subtomogram average, slice through the reconstruction coloured by local resolution, and masked Fourier Shell Correlation (FSC) curve. **f**, Hdr-Vhu-Fwd subtomogram average coloured by local resolution, and masked FSC curve.

To determine whether the architecture observed by single-particle cryo-EM reflects the native organization of the complex, we performed cryo-electron tomography (cryo-ET) of vitrified *M. maripaludis* cells (Fig. 2a). We used a subtomogram averaging workflow using the abundant ribosomes as quasi-fiducial markers to refine tilt-series alignment and improve tomogram quality (Extended Data Fig. 4). The refined tomograms revealed large ring-shaped densities approximately 50 nm in diameter (Fig. 2c), consistent with the expected size and shape of the Hdr–Vhu–Fwd super-assembly.

**Extended Data Fig. 5:**
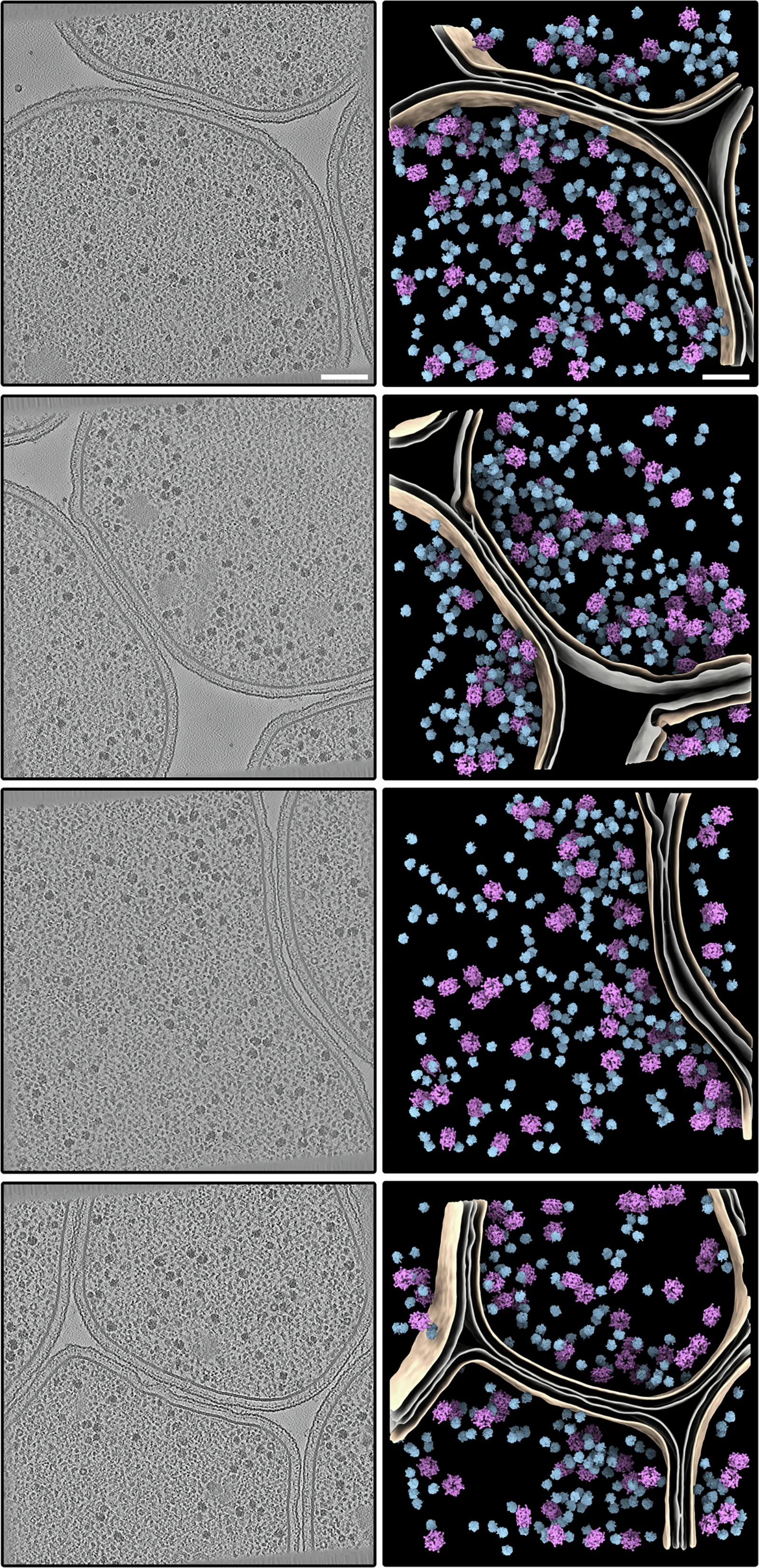
Gallery of representative tomograms showing intracellular Hdr-Vhu-Fwd distribution; representative features were consistently observed across multiple tomograms. Central slices from representative denoised tomograms of *M. maripaludis* are shown on the left. Slice thickness, 7.72 Å. Corresponding views on the right show back-projected Hdr-Vhu-Fwd super-assemblies in pink and ribosomes in blue. Membranes and S-layer segmentations are shown in tan and grey, respectively. Scale bar, 100 nm.

We then used template matching to systematically pick these particles across the tomograms, and found that the complex is distributed throughout the cytoplasm (Fig. 2b), at an apparent concentration of 1.9 ± 0.3 μM (Fig. 2d and Extended Data Fig. 5). This cytoplasmic localization contrasts with earlier immunogold labelling studies of non-F_420_-reducing hydrogenases, which suggested a membrane-associated distribution^26^. To further characterize the in situ architecture of these assemblies, we performed subtomogram averaging of the ring-shaped particles, yielding an 11.1 Å reconstruction of the Hdr–Vhu–Fwd super-assembly (Fig. 2e and Extended Data Fig. 4f). The subtomogram average revealed a double-ring architecture that closely matched that of the purified complex determined by single-particle cryo-EM. Accordingly, docking of the single-particle model showed strong agreement with the in situ density, confirming that the dodecameric Hdr–Vhu–Fwd super-assembly forms within the cytoplasm of *M. maripaludis*. Together, these observations show that the architecture observed after purification faithfully reflects the physiological organization of this electron-transfer machinery in vivo.

## Overall architecture of the complex

**Extended Data Fig. 6:**
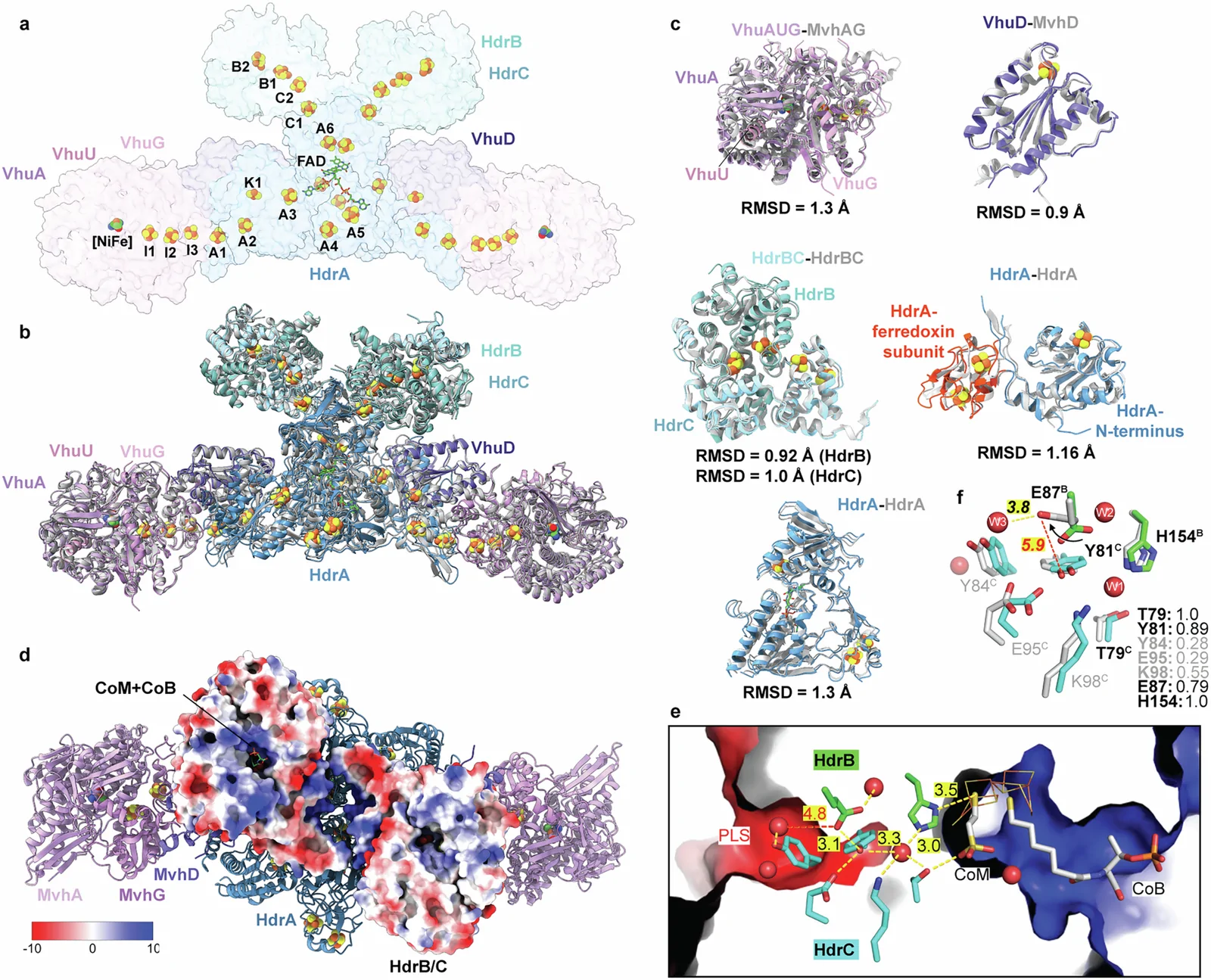
Hydrogenase VhuADGU-Heterodisulfide reductase HdrABC connection and proton transfer pathway. **a**, Surface representation of Vhu-Hdr with named cofactors. **b**, Alignment of the Hdr-Vhu complex from this study (coloured subunits) with the crystal structure from *Methanothermococcus thermolithotrophicus* (represented in grey colour)^14^. **c**, Individual subunits aligned with the crystal structure (same colour representation) and the corresponding root mean squared deviation (RMSD). **d**, Overview of the MvhADG:HdrABC complex from *Methanothermococcus thermolithotrophicus* as crystalised in the presence of FAD and heterodisulfide (PDB ID 5ODR)^14^. MvhAG is equivalent to [NiFe]-hydrogenase VhuAUG and MvhD resembles VhuD (**b, c**). Connected by the bifurcating HdrA subunit, the catalytic dimer HdrBC is depicted in surface representation where red and blue hint at preliminary negative and positive charge, respectively (calculated via the Adaptive Poisson-Boltzmann Solver)^93^. Note that only the right HdrBC dimer contains CoM and CoB in the active site pocket. **e**, This dimer reveals a negatively charged, water-filled proton loading site (PLS) and a positively charged heterodisulfide binding site. The latter is occupied by CoB and CoM as formed upon reduction of heterodisulfide and the non-cubane [4Fe-4S] clusters (shown as lines). Charged amino acid residues form a proton transfer pathway (PTP) between PLS and reactants, in particular histidine H154 and glutamate E87 of HdrB, tyrosine Y81 of HdrC as well as water molecule W1 at the interface of HdrB and HdrC. Residues T79, Y84, E94, and lysine K98 of HdrC may serve to stabilize further water moieties (W2, W3) and the CoM reactant (see panel **c** for labels). Starting in the PLS, we propose that protons proceed via W3, E87, Y81, W1, and H154 toward the thiol group of CoM. The distances range between 3.0–3.5 Å and justify the assumption of efficient proton transfer^94^; however, at 4.8 Å E87 seems to be too far away from W3 in the PLS for any proton transfer reactivity. **f**, Comparison between key residues of HdrBC from *M. thermolithotrophicus* (green and cyan sticks) and *M. maripaludis* (white sticks). While the overall architecture of the PTP is conversed between both organisms, it is notable that the side chain of E87 is oriented toward a (potential) water molecule in *M. maripaludis* rather than Y81 as observed in *M. thermolithotrophicus*. This reduces the distance between W3 and E87 by 1 Å but nearly doubles the distance between E87 and Y81 from 3.1 Å to 5.9 Å. Please note that individual water molecules where not resolved in our cryo EM structure; therefore, the position of W1-W3 from PDB ID 5ODR was utilized in the analysis. We speculate that glutamate E87 serves as a gateway connecting PLS and PTP, the latter which delivers protons to the heterodisulfide binding site at the other end of the HdrBC dimer. Residues primarily involved in proton transfer are well conserved (identity 0.79-1.0, see table in the right corner).

The overall architecture of the class I Hdr super-assembly consists of two hexameric rings, each containing six HdrABC–VhuADGU dimeric complexes (Fig. 1). The individual HdrABC dimers are equipped with two hydrogenase domains as electron-input modules, highly similar to that of the Mvh–Hdr complex from *M. thermolithotrophicus*^14^ (Extended Data Fig. 6). Located in the HdrA subunit, each Hdr protomer has an electron-bifurcating FAD cofactor that binds in the typical FAD-binding pocket conserved in a Rossmann fold^27,28^. The HdrB contains two non-cubane [4Fe-4S] clusters that function as the active site for the reduction of CoM-S-S-CoB^14^ (Extended Data Fig. 3b).

**Fig. 3:**
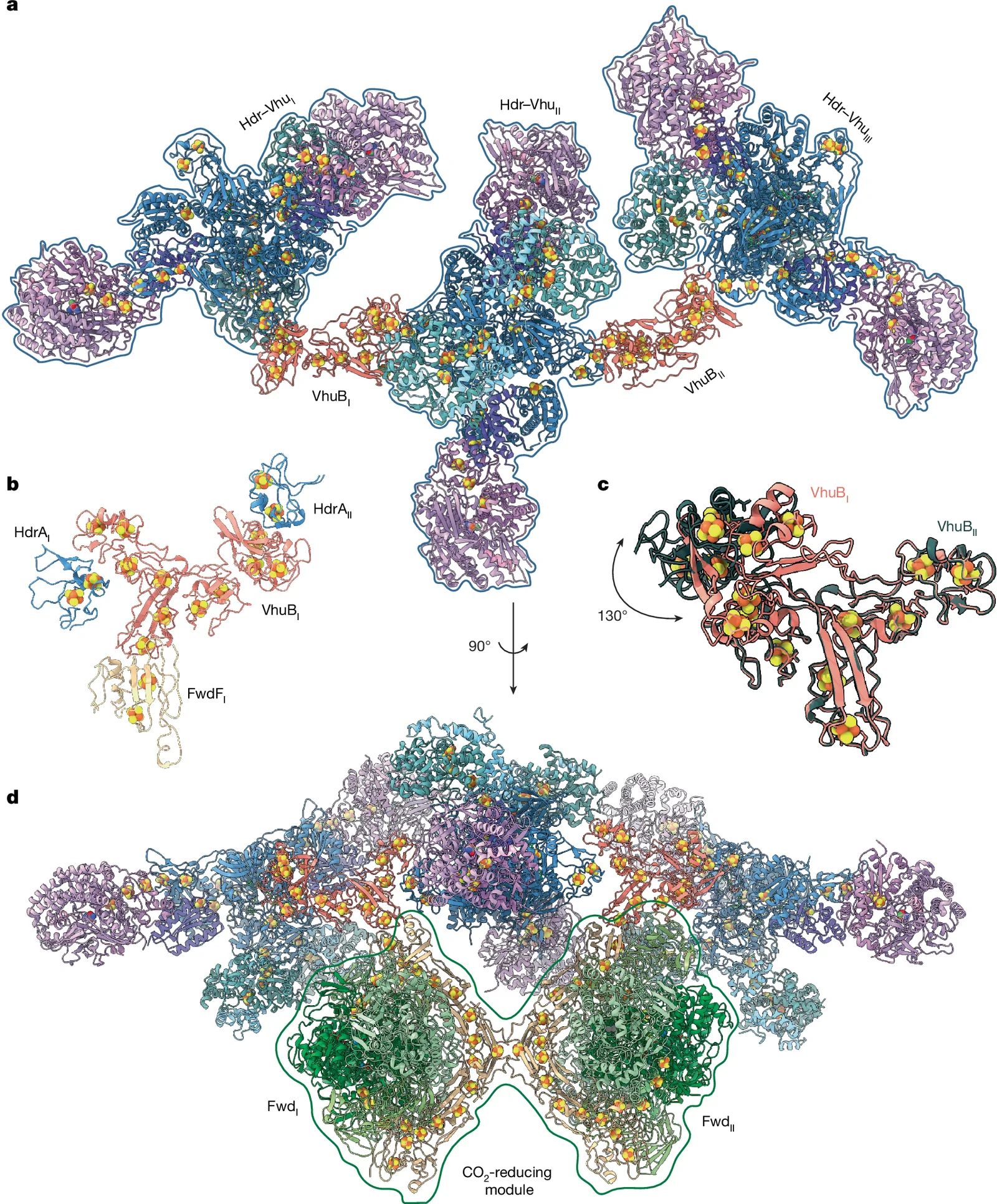
Connection of the electron-input module with the CO_2_-reducing module through a polyferredoxin. **a**, Connection of the Hdr–Vhu dimers with VhuB in different conformations. **b**, Interaction of VhuB with HdrA and FwdF. **c**, Conformational change of one ferredoxin domain within VhuB to facilitate HdrA binding. **d**, Addition of the CO_2_-reducing module (Fwd) binding to VhuB and the Hdr–Vhu dimers.

**Extended Data Fig. 7:**
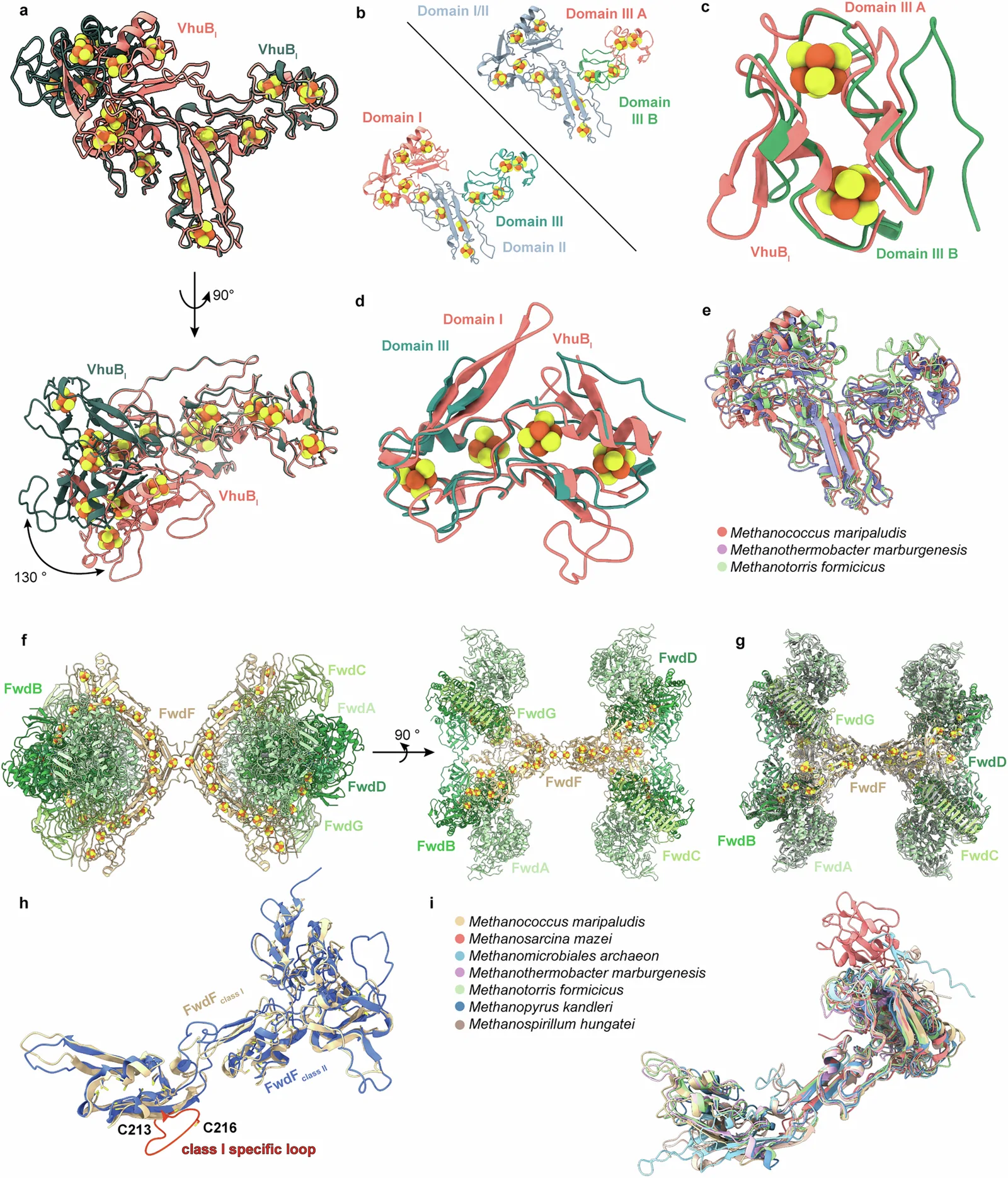
Polyferredoxin structural superpositions and formylmethanofuran dehydrogenase. **a**, Both VhuB polyferredoxins facing in different directions aligned to each other revealing a 130 ° tilt of Part I. **b**, Separation of VhuB in three parts. **c**, Alignment of two ferredoxin parts of the third part and in **d** for the parts I and two showing high similarity of the different ferredoxin subunits that are attached to HdrA (see Fig. 3). **e**, Alignment of VhuB from *M. maripaludis* (this study) with MvhB from *M. marburgenesis* (Uniprot: P60232)^95^ and an AlphaFold3 prediction^73^ from *M. formicicus* (Uniprot: H1KZP8). **f**, Model of the formylmethanofuran dehydrogenase from front and from top highlighting the hourglass shape. **g**, Alignment of the individual subunits of Fwd (coloured) with the crystal structure of *Methanothermobacter wolfeii* (represented in grey)^96^. **h**, Flexible alignment using FATCAT^97^ from FwdF of a class I methanogen (tan) and a class II methanogen (blue)^3^. **i**, Alignment of FwdF from this study with FwdF AlphaFold3^73^ predictions and published models from different organisms (Uniprot IDs: A0A0F8UQE2 (*M. mazei*), A0A7K3YAW2 (*M. archaeon*), D9PU51 (*M. marburgenesis*), H1KX37 (*M. formicicus*), Q8TYJ0 (*M. kandleri*) and PDB: 7BKB^3^ (*M. hungatei*)).

Essentially, these bifurcating HdrABC subcomplexes are connected through the VhuB polyferredoxin subunit. The VhuB polyferredoxin consists of three modular units arranged in a V-shaped orientation (Fig. 3 and Extended Data Fig. 7). Structurally, each domain features a double ferredoxin fold that is characterized by two CX_2_CX_2_CX_3_CP sequence motifs^29^, as commonly observed in classical ferredoxins containing two [4Fe-4S] clusters. Within the single unit, the second domain of the VhuB ferredoxin forms an extended internal β-hairpin structure^30^ (Fig. 3 and Extended Data Fig. 7). The N-terminal and C-terminal (domains I and III) domains of the ferredoxin are nearly identical with high sequence similarity, suggesting a gene-duplication event (Extended Data Fig. 7c,d). Using their extended β-hairpin fold, VhuB binds to the ferredoxin domain of HdrA (Fig. 3). This intricate architecture of fused polyferredoxin proteins allows VhuB to link two Hdr(ABC)_2_ subcomplexes in opposite orientations (Fig. 3a), creating an alternating upward–downward arrangement of HdrABC dimers within each ring. Depending on their orientation, the HdrBC subunits face either outward or inward. By contrast, the VhuB serves as a platform for the HdrABC–Vhu subcomplex to bind to from the opposite sides, whereas the organization in the upper and lower ring is identical (Fig. 1c,d).

The two rings are interconnected through two polyferredoxins VhuB and FwdF. In contrast to the V-shaped VhuB, the polyferredoxin FwdF is linear and contains four ferredoxin protomers, each with two [4Fe-4S] clusters, that mediate electron transfer towards the Fwd complex^3^ (Fig. 3d and Extended Data Fig. 7). The architecture and overall composition of the FwdABCDFG tetramer closely resembles that of *M. wolfeii*^12^, forming a hourglass-like structure (Extended Data Fig. 7g). Similarly, the tetramer is bridged by two additional central [4Fe-4S] clusters that can be bound only in a tetrameric organization of the Fwd complex (Fig. 3d and Extended Data Fig. 7f–h). This architecture connects the two rings by binding to two VhuB polyferredoxins in the upper ring and two in the lower ring, resulting in the distinctive X-shaped appearance of the tetrameric FwdF polyferredoxin core (Fig. 3d and Extended Data Fig. 7f). As a result, these proteins bridge the upper and lower Hdr–Vhu complexes in an overall* D*_3_ symmetric arrangement, creating the observed super-assembly architecture.

## Electron transfer and catalytic coupling

**Fig. 4:**
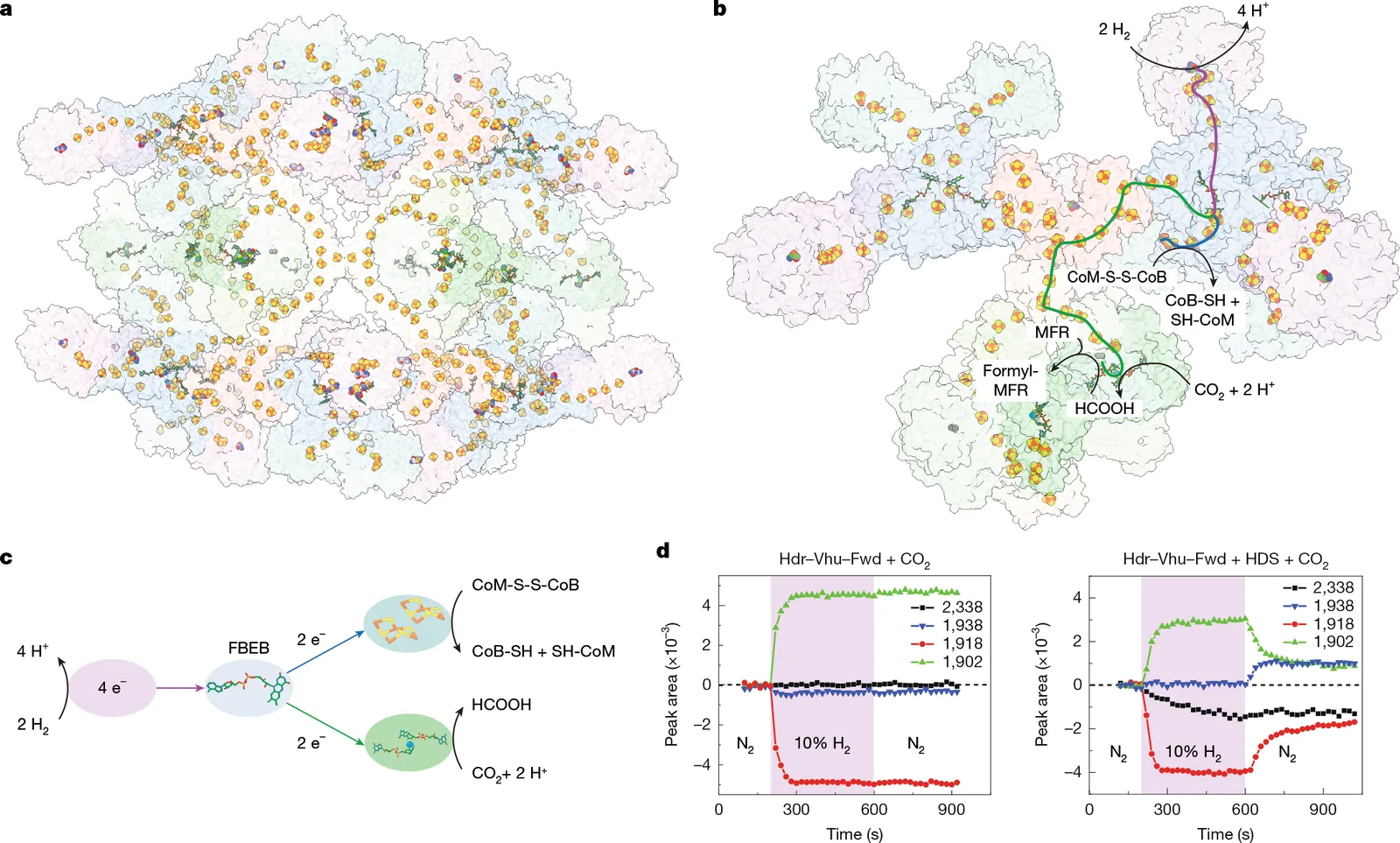
Electron-transfer pathway from the oxidation of H_2_ to the reduction of CO_2_. **a**, Surface representation of the model, highlighting the cofactors of the complex. **b**, Electron pathway going from oxidation of H_2_ (red) to the bifurcating FAD, transferring the high-potential electron to the non-cubane [4Fe-4S] cluster for the reduction of CoM-S-S-CoB (blue) and the low-potential electron to Fwd for the reduction of CO_2_. **c**, Graphical representation of FBEB and the reducing/oxidizing steps. **d**, Redox-controlled infrared measurement was performed in a gas-controlled chamber. Hydrogenase was activated by switching from N_2_ to H_2_, followed by the removal of H_2_. Carbonyl bands at 1,938, 1,918 and 1,902 cm^−^^1^ are assigned to the Ni-R, Ni-SI and Ni-L intermediates, respectively. The band at 2,338 cm^−1^ is assigned to CO_2_. CO_2_ was supplied under both conditions as a low-potential electron acceptor in Fwd, and heterodisulfide (HDS) was added (right) as a high-potential electron acceptor in HdrB.

**Extended Data Fig. 8:**
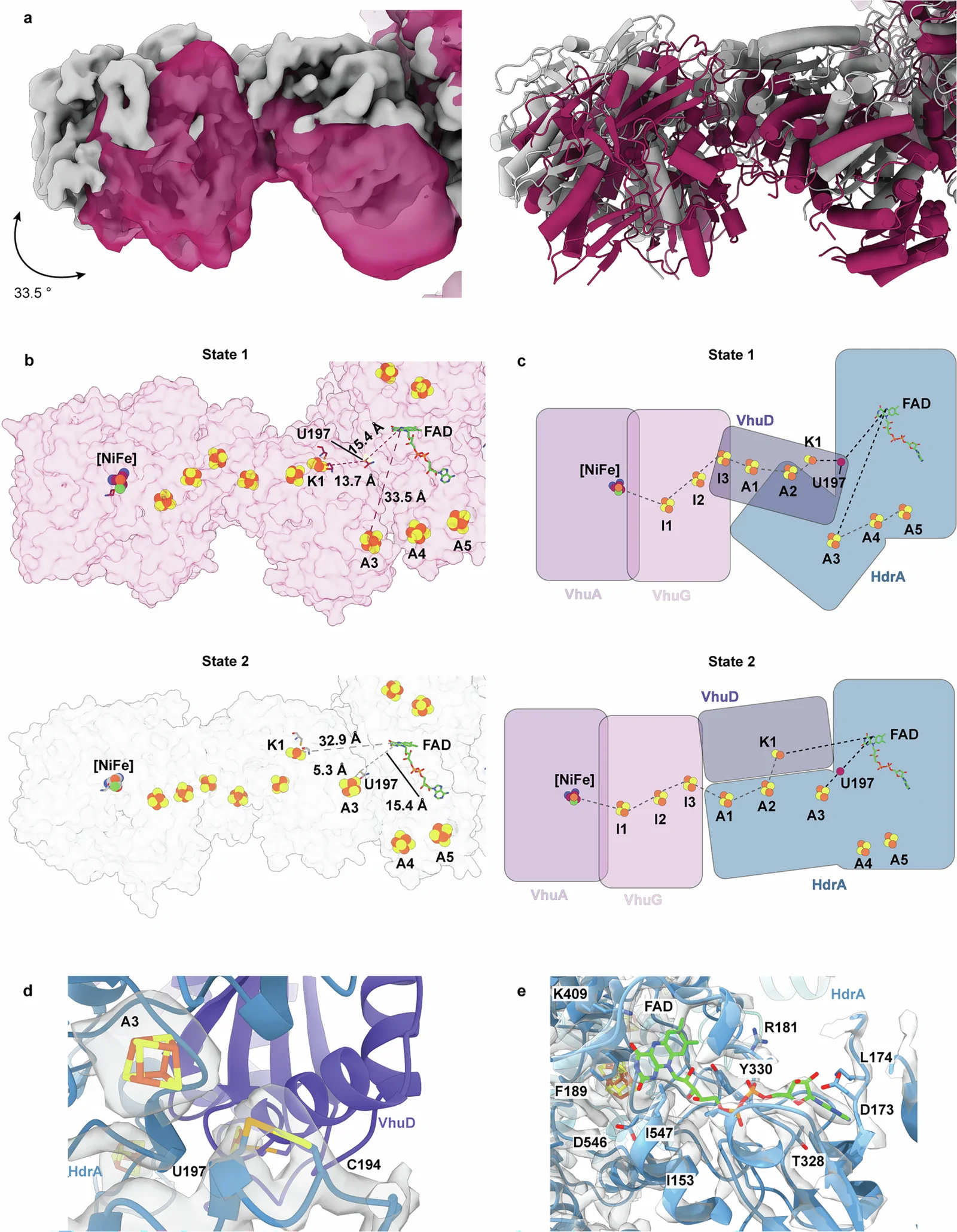
Movement of the mobile hydrogenase arm. **a**, Cryo-EM maps of the focussed refined electron input module after 3D classification analysis (left) and the corresponding, fitted models (right). **b**, Two conformational states enabling electron transport from the oxidation of H_2_ at the [NiFe] cofactor to the FAD. State 1 showing the electron-transfer from the [NiFe] cofactor to the [2Fe-2S] cluster K1 and finally to the bifurcating FAD. Due to conformational changes, the FAD comes into closer proximity to the [4Fe-4S] cluster A3 in the N-terminal domain of HdrA, enabling the electron transfer to the ferredoxin. To bridge the long distance between the iron-sulphur clusters and the FAD, the selenocysteine U197 may be involved in enabling rapid electron transfer. **c**, Schematic representation of the two conformational states displayed in **b**, highlighting the movement of the individual subunits and the clusters involved in the electron transfer. **d**, Cryo-EM map at the U197 indicating a selenosulphur bond. **e**, Highlighting the interactions of FAD embedded in HdrA where flavin-based electron bifurcation takes place.

Our structural model reveals the electron-transfer pathway extending from H_2_ oxidation at the [NiFe] active site in VhuADGU to CO_2_ reduction by the tungstopterin active site in FwdBC (Fig. 4). A central component to this chain is the bifurcating FAD in HdrA of the HdrABC homodimeric subcomplex (Extended Data Figs. 6a and 8e). Here the electron flow diverges into high- and low-potential branches of the bifurcation reaction^31^ in the HdrABC subcomplex (Fig. 4b,c). In the high-potential pathway, electrons flow from the bifurcating FAD through a chain of [4Fe-4S] clusters in HdrA and HdrC to HdrB, where two non-cubane [4Fe-4S] clusters form the active site for CoM-S-S-CoB reduction. A set of conserved residues promotes proton transfer from the surface to the active site niche (Extended Data Fig. 6d–f). Concurrently, the low-potential pathway directs the electron flow from the bifurcating FAD to the inserted ferredoxin domain of HdrA (Extended Data Figs. 6c and 8a,b). Located in the C-terminal Fd-like region, the two [4Fe-4S] clusters are directly linked to the polyferredoxin VhuB that connects HdrA with FwdF. The FwdF subunit mediates electron transfer over a series of [4Fe-4S] clusters to tungstopterin cofactor in FwdBC.

**Extended Data Fig. 9:**
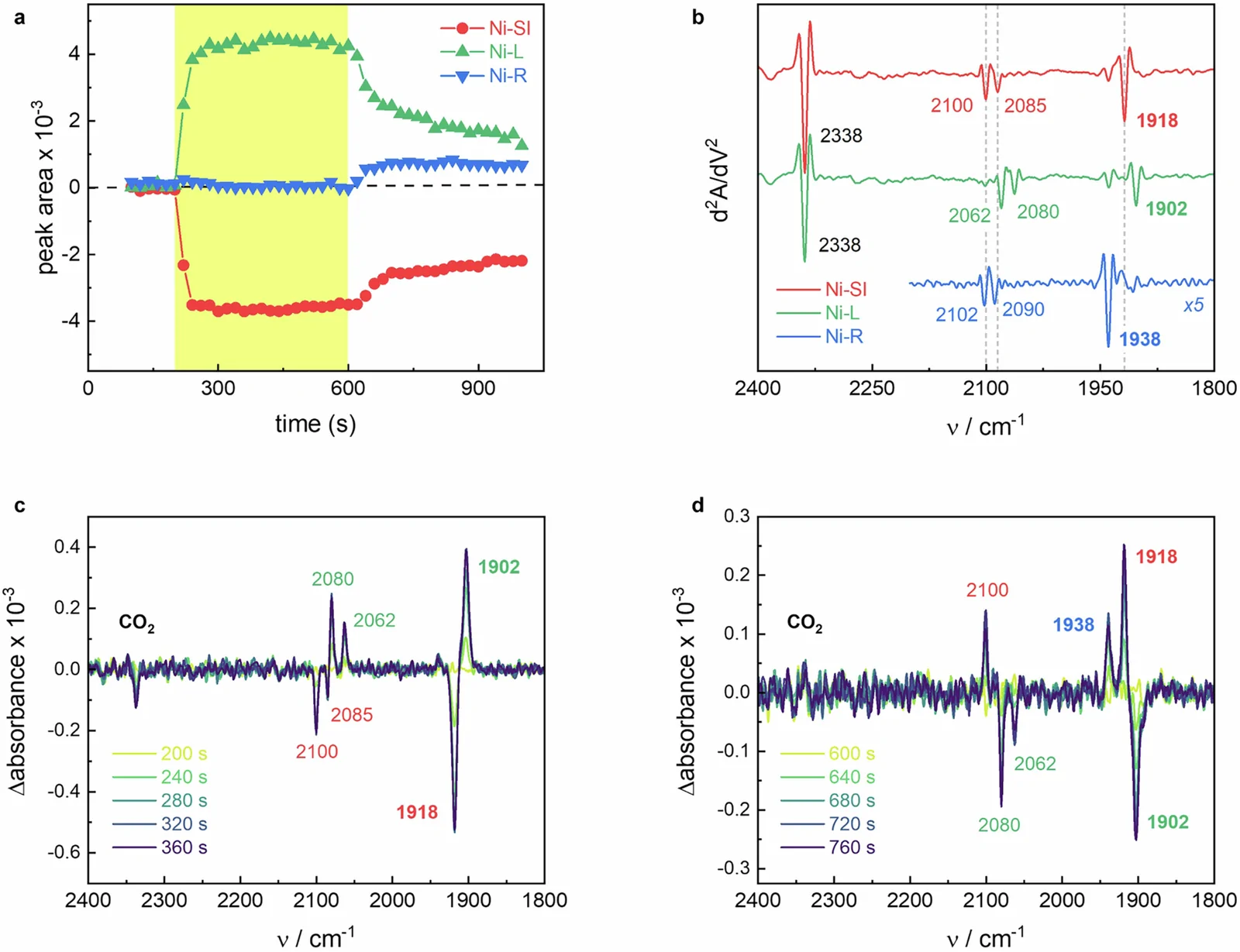
Infrared characterization. **a**, Kinetic traces of Hdr-Vhu-Fwd in the presence of heterodisulfide but without CO_2_. The conversion of Ni-SI into Ni-L under 10% H_2_ (yellow area) shows the reduction of Vhu and when the gas phase was switched back to N_2_, re-oxidation toward Ni-SI was observed (additionally, the data hint at a small fraction of Ni-R). We note that electron efflux appears to be functional in the absence of CO_2_. **b**, Second derivative analysis of the spectra from panel **a** in the cofactor regime from 2400 to 1800 cm^−1^. In the absence of H_2_, Vhu adopts the oxidized Ni-SI state (red trace). Upon reduction with H_2_, the enzyme converts into the one-electron-reduced Ni-L state (green trace). Both spectra show the absorbance of CO_2_ around 2339 cm^−1^. A third cofactor intermediate is hinted at by the band at 1938 cm^−1^. Careful subtraction allows isolating the whole CO/CN signature, which is assigned to the two-electrons-reduced Ni-R state (blue trace). Supplementary Table 5 shows the characteristics of all identified cofactor states, and Supplementary Fig. 2 explains the catalytic cycle of [NiFe]-hydrogenase. Based on these data, redox changes can be followed in real-time. **c**, “H_2_-minus-N_2_” difference spectra depict the conversion of Ni-SI into Ni-L from 200–360 s (comp. Figure 3d). Notably, this redox change is correlated with CO_2_ consumption, as indicated by the negative band at 2337 cm^−1^. The frequency of CO_2_ is slightly lower than in the 2^nd^ derivative spectrum in panel **b**, reflecting the hydrophobicity within the Fwd active site. **d**, “N_2_-minus-H_2_” difference spectra depict the re-conversion of Ni-L into Ni-SI and Ni-R from 600–760 s (comp. Figure 4d). Within the noise level of the experiment, oxidation of Vhu is not correlated with changes in the CO_2_ regime. The peak area of the bands in the difference spectra in panel **c** and **d** is plotted against time in Fig. 4d of the main script. Experiments with Hdr-Vhu-Fwd in the absence of heterodisulfide were carried out accordingly. Source data

To probe the electronic coupling within this super-assembly, we used in situ Fourier-transform infrared spectroscopy^32^. For this, the carbonyl absorbance bands of the [NiFe]-hydrogenase active-site cofactor^33^ of VhuADGU are followed in time as a function of the gas composition across the sample film, that is, 10% CO_2_ in N_2_ with or without 10% H_2_. In the absence of heterodisulfide, Fig. 4d and Extended Data Fig. 9 show how VhuADGU becomes reduced after contact with 10% H_2_ as the one-electron-reduced Ni-L state (1,902 cm^−1^) is populated over the oxidized Ni-SI state (1,918 cm^−1^). The Ni-C state, a characteristically stable intermediate of [NiFe]-hydrogenase^34^, is not observed (Supplementary Fig. 1). The concentration of CO_2_ bound to formate dehydrogenase FdhAB (2,338 cm^–1^) is unaffected by the reaction with H_2_ and, when the atmosphere was switched back to 0% H_2_, the hydrogenase remained in the reduced Ni-L state. However, in the presence of chemically synthesized heterodisulfide (Supplementary Fig. 2), a marked decrease of CO_2_ was observed after reduction by 10% H_2_ (Fig. 4d and Extended Data Fig. 9), indicative of CO_2_-to-formate conversion through FdhAB under electron-bifurcating conditions. Without H_2_, the CO_2_ concentration remained stable. The electronic coupling becomes additionally evident when VhuADGU reverts back into a redox equilibrium between Ni-SI, Ni-L, and the two-electron-reduced Ni-R state (1,938 cm^−1^) in the absence of H_2_ (Supplementary Table 5). Importantly, this behaviour was not observed when heterodisulfide was absent from the reaction mixture (Fig. 4d and Supplementary Fig. 1), indicative of impaired electron efflux toward Hdr and Fwd.

## Conformational flexibility and gating

To gain mechanistic insights, we performed extensive 3D classification and variability analysis on the Vhu electron-input module attached to the bifurcating subunit HdrA (Extended Data Fig. 8a). Our structural analysis indicates that the conformational flexibility within the HdrABC subcomplex underlies the gating of electron transfer, consistent with earlier mechanistic proposals^3^. Comparison with previous structures^3,14^ suggests that our reconstruction represents the reduced state 2 (Extended Data Fig. 8b), in which connectivity is established between the A3 [4Fe–4S] cluster of HdrA and the bifurcating FAD cofactor, enabling electron transfer towards the low-potential pathway (Fig. 4 and Extended Data Fig. 6a). However, in the oxidized state 1 (Extended Data Fig. 8b), the mobile N-terminal domain of HdrA repositions in a way that the K1 [2Fe-2S] cluster of VhuD forms the primary connection to the flavin, enabling electron transfer to FAD. Further flexibility analyses revealed pronounced motions in VhuD and the N-terminal region of HdrA, supporting a gating model in which dynamic repositioning of these modules regulates access to FAD (Fig. 4 and Extended Data Fig. 8).

The edge-to-edge distance between the A3 [4Fe–4S] cluster and the FAD cofactor (around 33 Å; Extended Data Fig. 8b,c) clearly exceeds the range of direct electron tunnelling^35^, implying the presence of an intermediate relay. Inspection of the cryo-EM map revealed a distinct, continuous feature located between A3 and FAD in state 2, coordinated by a cysteine and a selenocysteine residue Sec197 (Extended Data Fig. 8b,d). Given that HdrA contains sequence and structural features characteristic of pyridine-nucleotide-dependent thioredoxin-like fold^36,37^, it is plausible that this feature corresponds to a redox-active disulfide or selenylsulfide bridge that transiently mediates electron transfer between the two cofactors^38,39^. In canonical thioredoxin reductases, electrons are transferred from the reduced flavin to an adjacent dithiol or selenolthiol centre, which then undergoes reversible thiol-disulfide or selenenylsulfide exchange reactions, coupled to conformational changes that gate access to downstream substrates. A related mechanism operates in DsbD-mediated electron transfer across the bacterial inner membrane^16,40^ whereby electrons are propagated through a series of cysteine pairs through sequential disulfide exchange reactions, effectively forming a protein-based redox relay that bridges large spatial separations. By analogy, the Cys–Sec pair could function as a reversible thiol–selenol electron shuffle^41^, transiently cycling between oxidized and reduced states to bridge the large distance between the buried FAD cofactor and distal iron–sufur clusters^42^. The involvement of selenocysteine residues in this reaction is not strictly required, as they are not conserved across all methanogenic archaea^43^. Nevertheless, their incorporation can be crucial for redox catalysis, owing to their enhanced nucleophilicity^44^ and reactivity relative to thiol groups. Substitution of cysteine with selenocysteine has the potential to enhance catalytic efficiency in electron-transfer processes^45,46^. At present, the precise role of the Cys–Sec pair, its coupling to FAD, and the potential contribution of selenocysteine to redox tuning remain unresolved, representing an open question that will require further structural, spectroscopy, and functional studies.

## Modular flexibility of Hdr assemblies

**Fig. 5:**
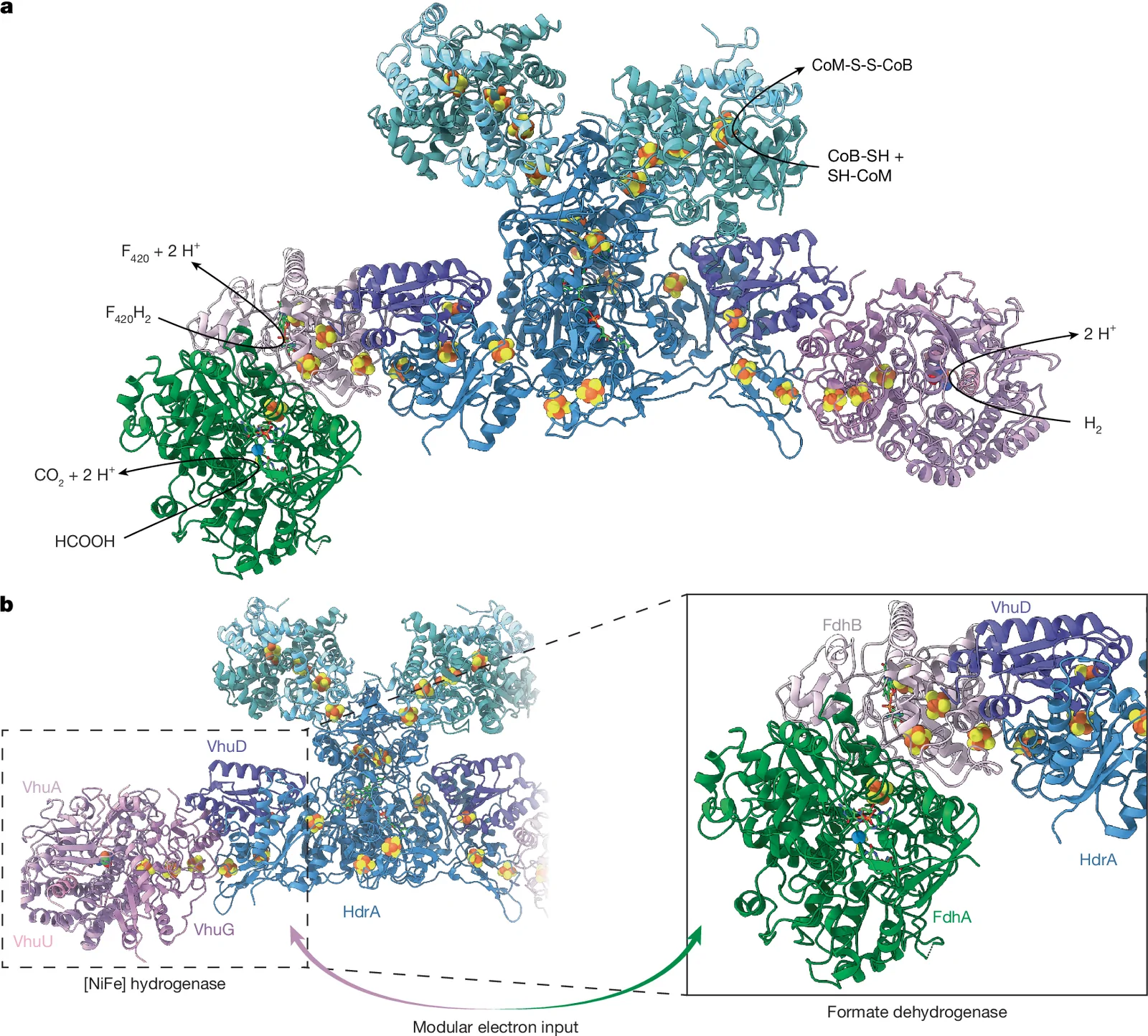
Modular exchange of the electron-input module. **a**, Model of the Hdr dimer with a hydrogenase binding on one side and a formate dehydrogenase binding on the other side. **b**, Magnified representation of the electron-input modules showing the cofactors.

Our 3D-classification analysis of the electron-input module (Extended Data Fig. 1) revealed not only large-scale motions of the hydrogenase but also the presence of an alternative electron-input module positioned at the inner arm of HdrABC within the super-assembly. In approximately 18% of the particles, we observed that Hdr associates with formate dehydrogenase (FdhAB) (Fig. 5). This heterogeneity of the electron-input module revealed a structure of HdrABC where the Vhu is located on the exterior of the ring while FdhAB binds to the interior side. In both cases, binding is mediated by the N- and C-terminal region of HdrA together with a helix (N76–L93) of VhuD^21^ (Extended Data Figs. 3 and 6). Although Hdr has previously been shown to accommodate a variety of electron-input modules^16,47,48^, concurrent binding of two distinct modules has not been reported yet. This dual association probably reflects an adaptive mechanism to metabolic stress, such as H_2_ limitation^2^, underscoring the capacity of class I methanogens to thrive under extreme conditions^20^. This dual-binding mechanism highlights the notable adaptability of the Hdr scaffold, with the ability to engage multiple electron-input modules, and suggests that modular association with distinct partners underlies the evolutionary versatility of this central bioenergetic machine^49^.

## Architectural differences in methanogens

**Fig. 6:**
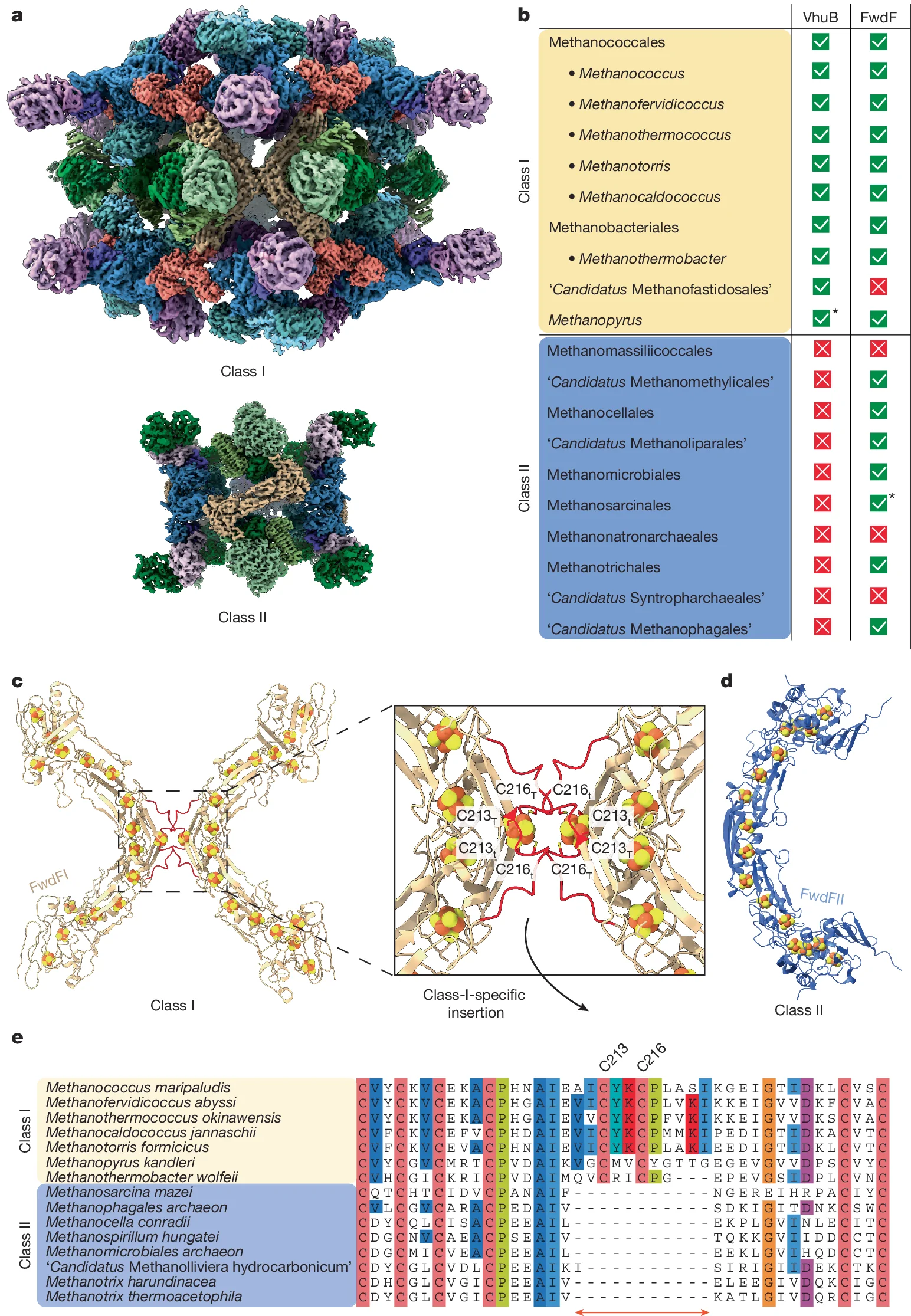
Polyferredoxins are responsible for different class I and II Hdr super-assemblies in methanogens. **a**, Comparison between the Hdr super-assemblies formed in class I (top) and class II (bottom)^3^ methanogens. **b**, The taxonomic distribution of polyferredoxins VhuB/FwdF in representative methanogen orders. Polyferredoxins marked with an asterisk within the protein group (VhuB or FwdF) have similar roles but differ in exact fold and sequence. **c**, The FwdF tetramer architecture in class I methanogens, binding two additional [4Fe-4S] clusters that are coordinated by the C213 and C216 cysteine residues of the FwdF chains T and t. **d**, The FwdF dimer structure in class II methanogens. **e**, Multiple-sequence alignment showing that cysteine residues conserved for cluster binding at the interface of the FwdF tetramer are present only in class I methanogens.

Overall, our structure shows considerable differences compared with the previously reported Mvh–Hdr–Fmd complex in *M. hungatei*—a class II methanogen^3^ (Fig. 6a). These architectural changes are largely attributed to variations in polyferredoxin subunit binding. In *M. hungatei*, FmdF homodimers bridge Hdr(ABC)_2_ and Fmd(ABCDG)_2_ units in a symmetric hexamer by binding to the N-terminal domain of HdrA’s internal ferredoxin-like domain. By contrast, in *M. maripaludis*, the structure reveals that the VhuB occupies this interface. As VhuB provides two binding sites to the dimeric Hdr(ABC)_2_ core, its incorporation leads to the formation of a dodecameric assembly. In this context, FwdF associates with the internal ferredoxin-like fold of VhuB, bridging the complexes in a V-shaped arrangement through a tetramer of FwdF subunits that assembles into a tetrameric hourglass configuration with Fwd(ABCDG)_4_ units. This alternative FwdF oligomeric state arises from additional [4Fe-4S] clusters located at the FwdF dimer–dimer interface (Fig. 6c–e). Together, these findings highlight that VhuB binding along with the supplementary dimer–dimer interface of FwdF are determinants of the distinct architecture of the *M. maripaludis* Hdr–Vhu–Fwd super-assembly.

To examine the distribution of at least two distinct Hdr super-assemblies in the methanogenic lineage, we analysed the diversity and taxonomic distribution of polyferredoxins across methanogenic Euryarchaeota (Fig. 6b). Our analysis reveals that homologues of VhuB are confined to *Methanococcus* and *Methanobacteria*, while they are entirely lacking in class II and TACK archaea. These patterns strongly indicate that the ancestral lineage of *Methanococcus* and *Methanobacteria* introduced this polyferredoxin, thereby establishing a characteristic electron-transfer architecture in hydrogenotrophic class I methanogens.

In contrast to VhuB, FwdF encodes a more broadly distributed polyferredoxin that is also present in methylotrophic TACK archaea. Its widespread occurrence suggests a functional role in metabolic pathways such as methylotrophy or alkane degradation. Furthermore, with the exception of Methanomassiliicoccales and Methanonatronarcheales, FwdF is conserved across most class II methanogens, indicating that the *M. hungatei* structure may represent the characteristic architecture of Hdr–Fmd complexes in this lineage. Methanosarcinales primarily rely on quinone-based electron transfer for energy conservation by using the HdrDE system^50^. Here CO_2_ reduction is predominantly driven by membrane potential rather than electron bifurcation; however, it remains unclear whether *Methanosarcina* couples HdrABC directly with the Fwd/Fmd complex or instead uses a soluble ferredoxin pool for electron transfer.

Further supporting the confinement of the *M. maripaludis* architecture to class I methanogens, a multiple-sequence alignment of *fwdF* reveals a conserved set of residues (Fig. 6e). This network of residues is responsible for coordinating two auxiliary [4Fe-4S] clusters that stabilizes the hourglass-shaped Fwd(ABCDFG)_4_ assembly. Importantly, they are found exclusively in organisms that also encode *vhuB*, reinforcing the finding that this structurally unique super-assembly configuration is present only in class I methanogens.

## Discussion

Here we describe the 8 MDa Hdr–Vhu–Fwd super-assembly from *M. maripaludis*—one of the largest metalloenzyme complexes reported to date. This dodecameric complex, formed by two interlinked hexameric HdrABC–Vhu rings bridged by a tetrameric FwdF core, adopts a cartwheel-like architecture that integrates H_2_ oxidation and CO_2_ reduction within a single continuous electron-transfer system.

Previous research demonstrated direct coupling between Hdr and formyl-methanofuran dehydrogenase. However, our structure reveals a larger and fundamentally different architecture, which was confirmed by in situ cryo-ET. At the core of this assembly lies a unique polyferredoxin-based architecture, in which class-I-specific VhuB subunits mediate higher-order oligomerization, while FwdF coordinates electron transfer between rings and towards the CO_2_-reduction site. By directly linking all active sites, this super-assembly is able to confine all stages of CO_2_ reduction within a single complex, effectively circumventing reliance on the cytoplasmic ferredoxin pool. The integrated Hdr–Vhu–Fwd system thereby enables ferredoxins to be allocated to energetically expensive reactions such as nitrogen fixation^51,52^, and cofactor biosynthesis^53,54^, independently of catabolic CO_2_ fixation^10^.

Only a limited number of biological systems reach comparable structural scales and, notably, many belong to energy-converting electron-transfer machineries, including respiratory chain complexes^55^, photosynthetic reaction centre–antenna assemblies^56^, filamentous nanowires such as the hydrogen-dependent CO_2_ reductase (HDCR) complex^57^, and other large assemblies described in methanogens^58,59^. In these systems, supramolecular organization establishes extended electron-transfer networks that minimize diffusional losses and stabilize low-potential electron flow between catalytic centres, providing an effective solution to the kinetic and thermodynamic constraints of biological redox processes.

Another particularly interesting aspect of this architecture is its modularity, enabling alternative electron-input modules to bind under H_2_ limitation^25^. Although previous studies have reported the association of different electron-input modules, our data show that this occurs at the level of an individual HdrABC dimer, demonstrating that substrate interchangeability is structurally encoded within the preassembled complex. This modularity is also consistent with findings that, under nickel limitation, Vhu can be exchanged with an alternative isoform of FdhA, also named F_420_-dependent electron-donating proteins (Elp)^48^, that lacks a functional molybdenum-selenium active site but retains F_420_-oxidizing activity. Similarly, under selenium limitation, the Vhu hydrogenase can be replaced with its isoform Vhc, which lacks selenocysteine, yet remains catalytically functional^16,34,60^.

The ecological implications of this distinction are substantial. In anaerobic environments—in which H_2_ and formate fluctuate independently, and nickel or selenium can be limiting—exchangeable electron-input modules allow these methanogens to sustain CO_2_ reduction and energy conservation through a rapid adjustment, without altering the bifurcation core. The ability to preserve a stable bifurcation and CO_2_ reduction core while rapidly adjusting electron-input modules is likely to be advantageous in fluctuating anaerobic habitats, and may offer class I methanogens a strategy to sustain methanogenesis under changing substrate and micronutrient availability. Such flexibility may be especially important in energetically marginal environments, including marine sediments and host-associated niches.

In this context, the Hdr–Vhu–Fwd super-assembly reveals how methanogens integrate electron bifurcation, CO_2_ reduction and modular electron donor flexibility, thereby establishing a structural basis for sustained function in dynamic anaerobic environments.

## Methods

### Plasmid cloning and transformation

To express Hdr in *M. maripaludis* JJ Δ*upt* (DSM 2067)^61^ (further referred as *M. maripaludis*), the shuttle vector pLW40^23^ was used as a backbone for inserting HdrB2 with a N-terminal Twin-Strep-tag (TS-tag). The gene (MMJJ_17970) was synthesized together with the TS-tag by Twist Bioscience. It was subsequently amplified using PCR with primers SP021 and SP061, which incorporated 30 bp overhangs. The plasmid was then digested with the restriction enzyme NsiI-HF from New England Biolabs. Subsequently, the plasmid and insert were fused by Gibson Assembly^62^.

For the transformation, 2 µg of DNA was added to 5 ml McC medium^63^ in an anaerobic culture tube that contained 50 µl of 2.5% Na_2_S, and was inoculated with 1 ml of fresh, natural competent *M. maripaludis* cells. The culture was flushed with H_2_/CO_2_ (80:20) and incubated at 37 °C with an agitation of 180 rpm for 24–48 h. Fresh McC medium with 1.25 µg ml^−1^ puromycin was then inoculated with 5% cells from the pre-culture. The culture was flushed with H_2_/CO_2_ (80:20) and incubated with agitation at 37 °C for 2 days. After multiple passages in puromycin-containing medium, the insertion of the plasmid was confirmed by PCR and Sanger sequencing, using primer SP023.

### Cultivation of *M. maripaludis* and purification

*M. maripaludis*, containing pLW40 with TS-tagged HdrB2, was cultivated in anaerobically sealed 2 l bottles with 700 ml McC medium^63^, 1.25 µg ml^−1^ puromycin and 1 ml of 2.5% Na_2_S solution. Cultures were inoculated with 2% of pre-culture and grown at 37 °C at 180 rpm for 48 h. Twice a day, the cultures were flushed with H_2_/CO_2_ (80:20). For the purification, all steps were performed under anoxic conditions in an anaerobic chamber (COY laboratory products) under a 95% N_2_/5% H_2_ atmosphere. Then, 10 l of *M. maripaludis* cells were collected at 12,000*g* for 35 min at 4 °C and resuspended in 40 ml of wash buffer (25 mM HEPES pH 7.6, 100 mM NaCl, 12.5 mM MgCl_2_, 20 µM FAD, (2 mM DTT)) and 1 mM phenylmethylsulfonyl fluoride. The lysate was sonicated for 10 min at 60% maximum intensity with a 30 s/30 s on/off cycle. The lysates were centrifuged again at 17,000*g* for 35 min at 4 °C before the supernatant was incubated with Strep-Tactin resin (IBA Lifesciences). The resin was washed with 10 column volumes of wash buffer and eluted with an addition of 2.5 mM desthiobiotin to the buffer. Four elution fractions were pooled and concentrated with a 30-kDa cut-off Amicon Filter (Merck Millipore). The protein concentration was determined using the Bradford assay^64^. To 5 µl of protein sample or BSA-standards, 195 µl Bradford reagent (100 mg l^−1^ Coomassie blue G-250, 50 ml l^−1^ ethanol and 100 ml l^−1^ phosphoric acid (85%)) was added and the absorbance at 595 nm was measured using the TECAN infinite M200 PRO system for quantification. For inductively coupled plasma MS (ICP-MS), the protein concentration was determined using the Pierce Dilution-Free Rapid Gold BCA Protein Assay from Thermo Fisher Scientific. Moreover, an SDS–PAGE (15%)^65^ was performed after each purification.

### Grid preparation

For cryo-EM SPA, grids were prepared immediately after protein purification in an anaerobic chamber. QUANTIFOIL R 2/1 copper grids with a 200 mesh base and carbon support were glow discharged with a current of 15 mA for 25 s in a PELCO easiGlow device (Ted Pella). Then, 4 µl sample at a concentration of 2.5 mg ml^−1^ was applied onto the grid and plunge-frozen in liquid ethane-propane. Freezing was automatically performed using the Vitrobot Mark IV (Thermo Fisher Scientific) at a blotting force of 8 for 8 s at 100% humidity and 4 °C.

### Data collection, processing and model building

First, an initial smaller screening dataset was acquired on the Jeol CryoArm200 microscope operated at 200 kV and armed with a K2 direct electron detector. The automated data collection was performed using SerialEM^66^; frames were recorded at a magnification of 60,000, which corresponds to a calibrated pixel size of 0.97 Å. All of the frames were recorded at a dose rate of 60 e^−^ A^−2^, fractionated into 50 frames. In total, 1,472 images were collected. The processing was performed using CryoSparc^67^. The images were initially motion-aligned and dose-weighted using Patch motion correction^68^ and the contrast transfer function (CTF) was estimated using Patch CTF estimation. Particles were manually picked on 105 micrographs and were subsequently used to train for training a topaz model^69^. After multiple rounds of topaz train, the best model was used to pick particles on entire dataset. The particles were then subjected to ab initio reconstitution with four models. The best clean ab initio class was further used for heterogeneous and homogeneous refinement and, finally, a non-uniform refinement was performed to reconstruct a map with a resolution of 5.91 Å.

For high-resolution reconstruction, we acquired data on the FEI Titan Krios G4 transmission electron microscope operated at 300 keV, equipped with a Falcon 4i direct electron detector (Thermo Fisher Scientific) and an Selectris X energy filter with a slit width of 10 eV. The automated data collection was performed using EPU, where the images were recorded with a defocus ranging between 0.5 and 2.0 µm at a dose of 60 e^−^ Å^−2^ in a counting mode at a magnification of ×130,000, which corresponds to a physical pixel size of 0.95 Å. In total two datasets with 10,709 and 20,894 images were recorded in EER format. As mentioned previously, the data processing was done on CryoSparc^67^. The images were fractionated in 60 frames and were motion corrected using patch motion correction^68^, followed by CTF estimation using Patch CTF estimation. Particles were picked manually from a subset of 1,000 micrographs and then used to train a topaz model^69^. Iterative rounds of training resulted in a model which was used to extract particles from the entire dataset with a box size of 800 pixels, however 4× pixelated. The previously generated map from the initial dataset was then used as an input volume for heterogeneous refinement to sort a clean class of particles more precisely, and these particles were segregated from the non-aligning particles. This refinement resulted in a class containing 110,231 particles, which we subjected to non-uniform refinement with a *D*_3_ symmetry. With this refinement, we could reconstruct a consensus map with a global resolution of 4.05 Å. To improve the quality of different domains within the whole complex, we performed symmetry expansion with a *D*_3_ symmetry and did focused refinement. To this end, a total of eight masks was created using UCSF Chimera (v.1.17.3) and ChimeraX (v.1.8)^70^. A mask was created for the HdrABC module, and one around the polyferredoxin VhuB for both conformations beside the Hdr stalk (named VhuB_I_ and VhuB_II_ in this study). Furthermore, for the Fwd module, we masked the FwdF dimer as well as the FwdABCDG domain on their outward- and the inward-facing position (named Fwd_I_ and Fwd_II_) within the ring structure. To additionally increase the resolution of the focused refined regions, all masks and symmetry expanded particles were recentred given the large size of the super-assembly and the resulting large box size. Recentring of the particles increased the resolution of the VhuB subunit to 3.30 Å and 3.19 Å, respectively. For the Hdr stalk, the local refinement resulted in a reconstructed map with a resolution of 3.04 Å, when the mask was created by excluding the N-terminal domain and the C-terminal ferredoxin domain. For Fwd, the complex was divided into three parts resulting in the FwdF dimer (3.17 Å) and the outward- and inward facing FwdABCDG domains (3.15 Å and 3.18 Å). 3D analysis of the Vhu hydrogenase arm revealed a high degree of flexibility. Masked local refinement of the VhuADGU, including the N-terminal and C-terminal region of HdrA, was carried out, followed by a 3D classification. Particles from suitable 3D classes were combined and further refined using local refinement. This refinement resolved a map with a resolution of 3.41 Å. For the inner-facing arm of the hydrogenase within the Hdr module, the analysis pipeline was repeated and resulted in two different 3D classes. Besides the hydrogenase, in around 18% of the classes, the hydrogenase domains were replaced by a different module. Local refinement of these classes resulted in a map with a resolution of 3.31 Å, enabling model building. ModelAngelo^71^ and a subsequent BLAST search of the sequence identified the unknown protein subunits as the formate dehydrogenase subunit FdhA2 and FdhB2.

For model building, structural predictions were generated with AlphaFold2^72^ and AlphaFold3^73^. The models were first rigid-body fitted into the local refinement maps using UCSF ChimeraX (v.1.8)^70^ and then manually fitted with Coot software (v.0.9.8.95)^74^. Cofactors and ligands were also fitted in the map using Coot and further refined real-space refinement using PHENIX (c)^75^.

### Cryo-ET sample preparation and focused ion beam milling

*M. maripaludis* cultures were grown as described above to an OD_600_ of 0.91. The cultures were then centrifuged (2,700g) and resuspended to an OD_600_ of approximately 45. Concentrated cells (5 μl) were applied to Quantifoil R1.2/1.3 200-mesh copper grids, and vitrified in a liquid ethane-propane mixture using the Vitrobot Mark IV operated inside an anaerobic chamber at 100% humidity and 10 °C with an 8 s blotting time. A Teflon ring was placed on the sample side to ensure back-blotting only.

For lamella preparation, grids were clipped into AutoGrids, mounted in a 35° pre-tilted shuttle (Thermo Fisher Scientific) and transferred to the Aquilos 2 cryo-focused ion beam/scanning electron microscope (cryo-FIB/SEM; Thermo Fisher Scientific). A protective organometallic platinum layer was deposited for 30 s using the gas-injection system. As the cells covered the grid squares as a continuous multilayer, a single lamella position was chosen near the centre of each square. In total, 45 lamellae were prepared by automated cryo-focused ion beam milling in AutoTEM (Thermo Fisher Scientific) at 30 kV, using successive beam currents of 300, 100, 50 and 30 pA for milling and final polishing. Milling was performed at an angle of 7°, corresponding to a 4° stage tilt. The milling recipe specified a lamella width of 15 μm, a lamella depth of 3 μm and a final target thickness of 160 nm. After overnight automated milling, lamellae were manually polished from the top using a 0.5° over-tilt to achieve a more uniform final thickness.

### Cryo-ET data acquisition

Tilt series were collected on the Titan Krios G4 transmission electron microscope operated at 300 kV and equipped with a Selectris X energy filter (10 eV slit width) and a Falcon 4i direct electron detector. Acquisition positions were selected manually in SerialEM^76^, and automated acquisition was carried out using PACEtomo^77^. In total, 150 tilt series were recorded in EER format, at 1.93 Å px^−1^. A dose-symmetric tilt scheme^78^ was used, with 2° increments over an angular range of ±55° relative to the lamella tilt. Each tilt series comprised 56 tilts and a cumulative electron dose of 150 e^−^ Å^−2^. Data were collected at target defocus values ranging from −1.5 to −3.5 µm, in 1 µm steps.

### Cryo-ET data processing and subtomogram averaging

Cryo-ET data were processed primarily using the Linux WarpTools-M v.2.0.0dev31 pipeline^79,80^ (Extended Data Fig. 4a). EER data were grouped to approximately 0.16 e^−^ A^−2^ per frame group before full-frame motion correction. Odd and even frame subsets were retained separately for downstream tomogram denoising. Tilt-image CTF estimation was performed on a 2 × 2 grid, after which tilt series were aligned using the AreTomo2 (v.1.0.0)^81^ implementation in WarpTools. Defocus handedness was assessed in WarpTools and subsequently inverted. Tilt series CTF parameters were then estimated, and 3D-CTF-corrected tomograms were reconstructed at 7.72 Å px^−1^.

To improve tomogram quality, ribosomes were used as quasi-fiducials for tilt series refinement in M. Ribosome positions were initially identified by template matching with pytom-match-pick (v.0.7.4)^82^ using a search template generated from a bacterial ribosome subtomogram average (Electron Microscopy Data Bank (EMDB): EMD-45266). This yielded 6,625 ribosome picks from the 50 highest-quality tomograms. 3D subtomograms were reconstructed in WarpTools at 3.86 Å px^−1^, imported into RELION 4.0 and subjected to 3D classification without alignment. As all classes represented ribosomes, the full set was retained for further processing. RELION 3D autorefinement produced an initial ribosome map with a global resolution of 7.8 Å. The ribosome particles were then resampled to 1.93 Å px^−1^, imported into M and used for three rounds of refinement of particle poses and tilt alignments, followed by two rounds of 2 × 2 image warp refinement. This procedure improved the ribosome reconstruction to 4.5 Å global resolution. The final parameters from M were then used to reconstruct optimized tomograms at 7.72 Å px^−1^.

Hdr–Vhu–Fwd super-assemblies were picked from the optimized tomograms by template matching with pytom-match-pick, using a search template derived from the composite single-particle cryo-EM map. This resulted in sharp score maps (Extended Data Fig. 4b–d), which were further processed with a dual-thresholding strategy incorporating a tophat filter^83^, yielding 2,851 Hdr–Vhu–Fwd particles from the 50 tomograms. Hdr–Vhu–Fwd particles were extracted in WarpTools as 2D stacks at 3.86 Å px^−1^ and imported into RELION v.5.0. False positives were removed by 3D classification without alignment (10 classes,* T* = 0.5), resulting in a curated set of 2,694 particles. These were refined in RELION initially without imposed symmetry and subsequently with* C*_3_ symmetry to a nominal resolution of 13.1 Å. Imposing higher (*D*_3_) symmetry was also tested, but resulted in a worse reconstruction, consistent with intrinsic flexibility of the super-assembly. The curated Hdr–Vhu–Fwd particle set was then resampled to 1.93 Å px^−1^ and imported into M together with the ribosome particles. In M, we initially performed several rounds of particle pose and image warp refinement (two rounds using a 2 × 2 grid, followed by two rounds using a 3 × 3 grid), followed by CTF refinement. Per-series and per-tilt weighting were then applied, and particle temporal trajectories were refined. A final refinement round included refinement of stage angles, 6 × 6 image warping, CTF and particle poses. This procedure yielded final reconstructions at global resolutions of 11.1 Å for the Hdr–Vhu–Fwd super-assembly and 4.1 Å for the ribosome.

### Tomogram denoising, segmentation and particle concentration analysis

For visualization and membrane segmentation, tomogram denoising and missing wedge restoration were carried out using IsoNet2.0.0-beta^84^. First, an initial denoising model was trained on the reconstructed tomograms with CTF correction enabled (cube size, 96; *B*-factor, 0) and applied to generate denoised tomograms. Masks were then generated automatically with s.d. and density percentiles both set to 50%. An initial round of IsoNet2 refinement was carried out for joint denoising and missing-wedge correction (missing-wedge weight, 200; cube size, 128; *B*-factor, 0). Masks were then regenerated from the processed tomograms, and a second round of refinement was carried out. The final trained network was then applied to generate the final denoised, missing-wedge-corrected tomograms used for visualization and segmentation.

Membranes were segmented from the IsoNet2 tomograms using membrain-seg^85^. For visualization, particles were mapped back into the tomograms in ChimeraX using the ArtiaX plugin^86^. To estimate particle concentrations, cytoplasmic masks were generated for 8 tomograms in Dragonfly (Object Research Systems) and used to calculate cytoplasmic volumes. Per-tomogram particle concentrations were then determined and expressed in μmol dm^−3^.

### Infrared spectroscopy

All experiments were performed on hydrated protein films in attenuated total reflection configuration using a Fourier-transform infrared spectrometer (Bruker Tensor27) equipped with a mercury cadmium telluride (MCT) detector cooled by liquid N_2_. All data were recorded with a spectral resolution of 2 cm^−1^ at 80 kHz scanning velocity. For 100 co-additions of interferometer scans in forward/backward direction, a temporal resolution of 20 s was achieved. Specifically, Hdr–Vhu–Fwd protein samples (around 100 µM) with or without heterodisulfide (650 µM) were dried and rehydrated on the silicon crystal of the attenuated total reflection cell under a stream 90% N_2_ and 10% CO_2_ (1 l min^−1^), humidified with 10 mM Tris/HCl (pH 8). Reduction was induced by switching the atmosphere to 80% N_2_, 10% CO_2_ and 10% H_2_ making use of digital mass flow controllers (Sierra) as reported earlier^32^. Difference spectra were calculated by subtracting the first absorbance spectrum (*t* = 0) from any later spectra. All experiments were conducted under anaerobic conditions (<2 ppm O_2_), ambient temperature and pressure, and in the dark.

### Sequence analysis

Sequences of FwdF of *Methanosarcina mazei* (WP_048038706.1), *M. maripaludis* (WP_181521371.1), *Methanofervidicoccus abyssi* (WP_131007595.1), *Methanothermococcus okinawensis* (DQVW01000100.1), *Methanotorris formicicus* Mc-S-70 (WP_007043806.1), *Methanococcus jannaschii* DSM 2661 (WP_010870679.1), *Methanothermobacter wolfeii* (WP_261599642.1), *Methanopyrus kandleri* DSM 6324 (WP_011018679.1), *Methanocella conradii* DSM 24694 (WP_014407032.1), ‘*Candidatus* Methanolliviera hydrocarbonicum’ (RZN69131.1), *Methanomicrobiales archaeon* (WP_014867622.1), *Methanothrix harundinacea* 6Ac (WP_014586169.1) and *Methanothrix thermoacetophila* DSM 6194 (WP_011696724.1) were aligned using muscle3.8.31_i86darwin64^87^. Alignments were inspected and visualized using AliView (v.1.28)^88^.

To assess the conservation of VhuB and FwdF across different orders of methanogens, all available archaeal genomes were downloaded from the Genome Taxonomy Database (GTDB)^89^ on 14 April 2025. A local BLASTP search was performed using the Sequenceserver interface^90^ against a customized database, using the VhuB (MMJJ_11370) and FwdF (WP_011171189) sequences from *M. maripaludis* as queries, with an *e* value of 1 × 10^−5^. Hits with an *e* value higher than 1 × 10^−30^ were not considered. The identified homologues were further validated based on sequence length and predicted structural features, as VhuB and FwdF possess distinct characteristic folds despite sharing ferredoxin-like domains.

### Metal quantification using ICP-MS

To quantify the metal content in the purified HDR sample, 67% (w/v) nitric acid (HNO_3_; superpure grade, Chemsolute) was added to 40 µl of the protein sample reaching a final concentration of 11% HNO_3_. The samples were mineralized at 80 °C for 4 h, then diluted with Chelex-treated water to a final concentration of 2% HNO_3_, resulting in a total volume of 250 µl. Indium (2 µg l^−1^) and germanium (20 µg l^−1^) (both VWR Merck) were added to each sample as internal standards. External calibration was conducted with ICP multi-element standard solution XVI (VWR Merck). Elemental analysis was performed using an iCAP Q ICP-MS (Thermo Fisher Scientific) equipped with an SC4DX autosampler (Elemental Scientific) and a MicroFlow PFA-100 nebulizer. Samples were introduced through a peristaltic pump and analysed for V^51^, Fe^56^, Co^59^, Ni^60^, Cu^63^, Zn^66^, Se^78^, Mo^95^ and W^184^. The ICP-MS was operated with an argon plasma gas at 15 l min^−1^, a helium/hydrogen (93/7%) collision gas at 5 ml min^−1^, an argon carrier gas at 0.72 l min^−1^ and an argon plasma makeup gas flow of 1 l min^−1^. Data acquisition was carried out in triplicate using Qtegra v.2.18 (Thermo Fisher Scientific). Blank values and quality thresholds were determined using protein buffer standards. Measured concentrations (µg l^−1^) were converted to molar units (µM) of metal per sample and normalized to the protein concentration for quantitative analysis.

### Protein analysis using nLC–MS/MS

To identify proteins in the purified HDR sample, a shotgun proteomics approach was applied using nano-liquid chromatography coupled with tandem MS (nLC–MS/MS) according to previously described protocols^91,92^. In brief, proteins were reduced with 12 mM DTT, alkylated with 40 mM IAA and digested overnight at 37 °C with 0.63 µg trypsin. Digestion was stopped by adding 1 µl formic acid, and the samples were centrifuged at 14,000*g* for 10 min. The supernatant containing the peptides was collected, dried, resuspended in 10 µl 0.1% formic acid and desalted using C_18_ ZipTips (Merck Millipore). After zip-tipping, the peptides were dried in a vacuum centrifuge. For protein MS, dried peptides were dissolved in 20 µl 0.1% formic acid, shaken for 10 min, sonicated for 30 s and transferred to glass screw vials (Waters) for analysis. A 6 µl aliquot of the peptide solution was injected into an UltiMate 3000 nHPLC system (Thermo Fisher Scientific). Peptide separation was performed on an Acclaim PepMap100 C_18_ column using eluent A (0.1% formic acid) and eluent B (80% acetonitrile, 0.1% formic acid) with the following gradient: 0–6 min, 4% eluent B; 7–126 min, linear increase to 55% eluent B; 127–131 min, linear increase to 90% eluent B; 132–138 min, re-equilibration at 4% eluent B. Peptides were ionized by electrospray (TriVersa NanoMate, Advion) and analysed on an LTQ Orbitrap Fusion Tribrid mass spectrometer (Thermo Fisher Scientific) in positive mode. The scan range was set to 350–2,000 *m/z*, with a resolution of 120,000, an AGC target of 4 × 10^5^ ions and a maximum injection time of 50 ms. The two most intense precursor ions (charge state +2 to +7, isolation window 1.6 *m*/*z*) were selected in quadrupole isolation mode and accumulated to an AGC target of 5 × 10^4^ ions or a maximum injection time of 120 ms. Fragmentation was performed by high-energy collision dissociation at a collision energy of 30%, and fragment ions were analysed in the Orbitrap at a resolution of 30,000.

Raw data were processed with Proteome Discoverer v.3.2.0.450 (Thermo Fisher Scientific) using the SequestHT search engine against the UniProt database for *M. maripaludis* deltae (taxonomy ID: 39152). Carbamidomethylation of cysteines (+57.021 Da) was set as a fixed modification, and methionine oxidation (+15.995 Da) as a variable modification. Trypsin was specified as the protease, allowing up to two missed cleavages. Precursor and fragment mass tolerances were set to 3 ppm and 0.1 Da, respectively. Peptides ranging from 5 to 144 amino acids were considered, and proteins were accepted at a false-discovery rate of ≤1%, ensuring high confidence in identification.

### Chemical synthesis of heterodisulfide

Coenzyme B (100 mg, 291 µmol) and coenzyme M sodium salt (951 mg, 5.8 mmol) were dissolved in water (10 ml) with NH_3_ (aq., conc., 0.50 ml). KI_3_ (0.5 M in water) was added dropwise until the solution remained slightly brownish. The solution was then acidified to pH 5 by dropwise addition of HCl (1 M). Subsequently, the solution was loaded onto a flash column (Büchi Flashpure Ecoflex C18, 120 g) pre-equilibrated with water containing 0.1% formic acid, and the product was eluted by using a linear gradient from water to acetonitrile (both with 0.1% formic acid) over 50 min. The fractions containing the crude product were snap-frozen and lyophilized. The resulting colourless gum-like solid was further purified by preparative HPLC (Nucleodur C18 HTec, 5 µm, 250*21, Machery-Nagel) with a linear gradient from water to acetonitrile (both with 0.05% trifluoroacetic acid) over 50 min. Product-containing fractions were immediately snap-frozen and lyophilized. The lyophilized gum was then dissolved in a minimum amount of water/methanol (50/50) and transferred to a 50 ml conic plastic centrifuge tube. A pre-chilled (4 °C) lithium perchlorate-acetone solution (3 g per 50 ml) was added, and the suspension was gently shaken and then allowed to stand for an additional 10 min at 4 °C. After centrifugation, the precipitate was washed four times with acetone, then dried with air for 20 min and final high-vacuum drying. The dried residue was dissolved in 1 ml of water, aliquoted and lyophilized. The final solid (105 mg, 217 µmol, 75%) was stored at −80 °C.

### Reporting summary

Further information on research design is available in the Nature Portfolio Reporting Summary linked to this article.

## Online content

Any methods, additional references, Nature Portfolio reporting summaries, source data, extended data, supplementary information, acknowledgements, peer review information; details of author contributions and competing interests; and statements of data and code availability are available at 10.1038/s41586-026-10744-9.

## Supplementary information

Supplementary Information Supplementary Tables 1–5 and Supplementary Figs. 1–3.

Reporting Summary

Peer Review file

## Source data

Source Data Fig. 2

Source Data Extended Data Fig. 9

## Figures and Tables

**Extended Data Table 1:**
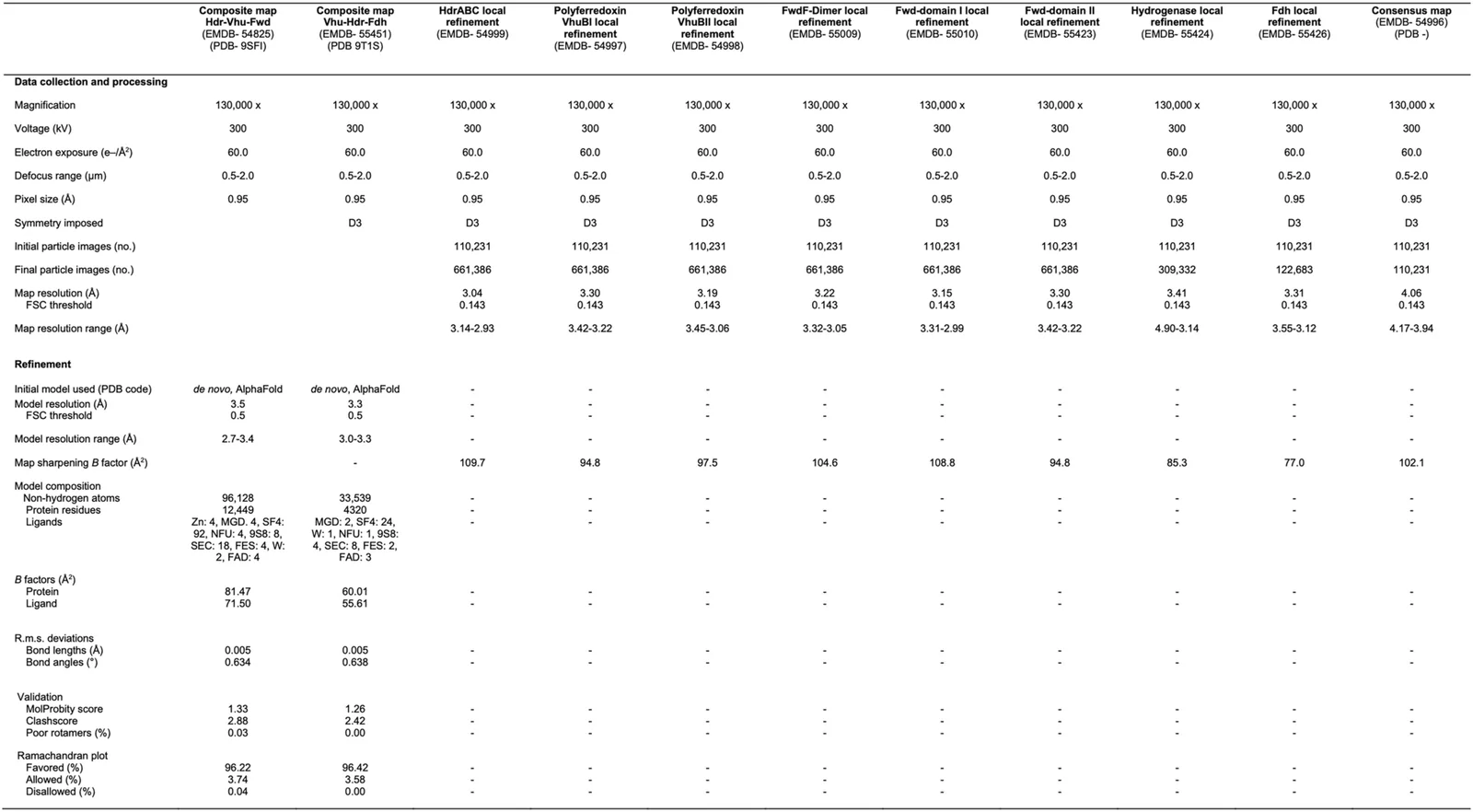
Cryo-EM data collection, refinement, and validation statistics

## Data Availability

The cryo-EM maps are available in the EMDB under accession codes EMD-54825 (composite map for the whole super-assembly) and EMD-55451 (composite map for the Hdr–Vhu–Fdh). Local refined cryo-EM maps are available under EMD-54996 (consensus map), EMD-54997 (VhuB_I_), EMD-54998 (VhuB_II_), EMD-54999 (Hdr(ABC)_2_), EMD-55009 (FwdF dimer), EMD-55010 (FwdI), EMD-55423 (FwdII), EMD-55424 (Vhu hydrogenase) and EMD-55426 (Fdh). The atomic models are available in the Protein Data Bank (PDB) under accession codes 9SFI (minimal repeating unit of the Hdr–Vhu–Fwd complex) and 9T1S (Hdr–Vhu–Fdh complex). The subtomogram averaging maps have been deposited to the Electron Microscopy Data Bank under accession numbers EMD-58202 (ribosome) and EMD-58201 (Hdr–Vhu–Fwd). Structural data that were used for comparison of Hdr–Vhu are available in the PDB (5ODC and 5ODR). For polyferredoxin VhuB comparison, MvhB from *M. marburgenesis* (UniProt: P60232) and an AlphaFold3 prediction from *M. formicicus* (UniProt: H1KZP8) was used. For the ferredoxin FwdF, the model from this study was compared with AlphaFold3 predictions and published models from different organisms: UniProt IDs: A0A0F8UQE2 (*M. mazei*), A0A7K3YAW2 (*M. archaeon*), D9PU51 (*M. marburgenesis*), H1KX37 (*M. formicicus*), Q8TYJ0 (*M. kandleri*) and PDB: 7BKB (*M. hungatei*). For sequence analysis of FwdF, sequences can be accessed at NCBI: *M. mazei* (WP_048038706.1), *M. maripaludis* (WP_181521371.1), *M. abyssi* (WP_131007595.1), *M. okinawensis* (DQVW01000100.1), *M. formicicus* Mc-S-70 (WP_007043806.1), *M. jannaschii* DSM 2661 (WP_010870679.1), *M. wolfeii* (WP_261599642.1), *M. kandleri* DSM 6324 (WP_011018679.1), *M. conradii* DSM 24694 (WP_014407032.1), ‘*Candidatus* M. hydrocarbonicum’ (RZN69131.1), *M. archaeon* (WP_014867622.1), *M. harundinacea* 6Ac (WP_014586169.1) and *M. thermoacetophila* DSM 6194 (WP_011696724.1) All available archaeal genomes were downloaded from the Genome Taxonomy Database (GTDB) on 14 April 2025 for sequence comparison. Strains and plasmids generated in this study are available from the corresponding authors on request. Source data are provided with this paper.
