# Supplementary material for: Architecture of the 8 MDa Hdr–Vhu–Fwd super-assembly in class I methanogens
Source: Nature. 2026 Jul 8;656(8129):1054–63. doi: 10.1038/s41586-026-10744-9 (PMC13518236; doi:10.1038/s41586-026-10744-9)
Supplement: Supplementary file 1 [file 41586_2026_10744_MOESM1_ESM.pdf]

nature portfolio

https://doi.org/10.1038/s41586-026-10744-9

Supplementary information

Architecture of the 8 MDa Hdr–Vhu–Fwd super-assembly in class I methanogens

In the format provided by the authors and unedited

Supplementary Information for

Architecture of the 8 MDa Hdr-Vhu-Fwd super-assembly in class I methanogens

Sophia Paul[1], Tomas C. Pascoa[1], Max A. Klamke[2], Stefan Bohn[3], Frank Abendroth[1], Darja Deobald[4], Olalla Vásquez[1,5], Sven T. Stripp[2], Jan M. Schuller[1,5*]

1 – Philipps-Universität Marburg, Department of Chemistry and Center for Synthetic Microbiology (SYNMIKRO), Karl-von-Frisch-Strasse 14, Marburg, Germany

2 – Spectroscopy & Biocatalysis, Institute of Chemistry, University of Potsdam, Karl-Liebknecht-Strasse 24/25, 14476 Potsdam, Germany.

3 – Helmholtz Munich Cryo-Electron Microscopy Platform, Helmholtz Munich, Neuherberg, Germany.

4 Department of Environmental Biotechnology, Helmholtz Centre for Environmental Research (UFZ), Leipzig, Germany.

5 - Microbes-for-Climate (M4C) Cluster of Excellence, Marburg, Germany.

Jan Michael Schuller, Center for Synthetic Microbiology (SYNMIKRO) and Department of Chemistry, Philipps-Universität Marburg, Marburg, Germany, Phone: +49-64212822584;

E-Mail: jan.schuller@synmikro.uni-marburg.de

* Corresponding author.

Supplementary information - Table of contents:

Supplementary Table 1- List of Primers **p. 3**

Supplementary Table 2- List of Plasmids **p. 3**

Supplementary Table 3- Protein components, verified by Mass-spectrometry **p. 3**

Supplementary Table 4- ICP-MS **p. 4**

Supplementary Table 5- Identified [NiFe] cofactor intermediates **p. 4**

Supplementary Figure 1- Catalytic intermediates of [NiFe]-hydrogenase **p. 5**

Supplementary Figure 2- NMR spectra of heterodisulfide **p. 6**

Supplementary Figure 3- Uncropped SDS-PAGE **p. 7**

Supplementary Tables:

Supplementary Table 1: List of primers

Name	Description	Primer sequence (5'-3')
SP021_fwd	To clone HdrB with a TS-tag in the shuttle vector pLW40	attttatagataactaataggtgaaatgcaATGTCAGCG TGGTCGCATCCCCAATTTGAG
SP061_rev		ttcgatgtgatggtgatggtggtgcatgcaTTttagtatcctaat ttttgaagtaatggttctggag
SP023_fwd	For sequencing the insertion of the HdrB sequence	taaaatttagctaataagctattga

Supplementary Table 2: List of plasmids

Name	Descriptions	Reference
pLW40	Shuttle vector for overexpression in *M. maripaludis*	Dodsworth *et al.*, 2006[1]
pLW40/HdrB	Plasmid for expression and Twin-Strep tag affinity purification of HdrB in *M. maripaludis*	This study

Supplementary Table 3: Protein components, verified by Mass-spectrometry.

Name	Description	Mol. Size (kDa)	Uniprot ID
HdrA	Heterodisulfide reductase, subunit A	71.2	A0A2L1CB19
HdrB	Heterodisulfide reductase, subunit B	32.0	A0A7J9NT94
HdrC	Heterodisulfide reductase, subunit C	20.4	A0A7J9PFQ6
VhuA	F420-non-reducing hydrogenase, subunit alpha	45.6	A0A2L1CAX5
VhuG	F420-non-reducing hydrogenase, subunit gamma	30.7	A0A7J9P3N2
VhuD	F420-non-reducing hydrogenase, subunit D	17.2	A0A2L1CBC1
VhuU	F420-non-reducing hydrogenase, subunit U	11.2	A0A2L1CB29
VhuB	Polyferredoxin	42.1	A0A2L1CB03
FwdA	Formylmethanofuran dehydrogenase, subunit A	63.3	A0A7J9NZ09
FwdB	Formylmethanofuran dehydrogenase, subunit B	48.6	A0A2L1CAZ7
FwdC	Formylmethanofuran dehydrogenase, subunit C	30.6	A0A2L1CC77
FwdD	Formylmethanofuran dehydrogenase, subunit D	14.1	A0A2L1CCC1
FwdF	Formylmethanofuran dehydrogenase, subunit F	38.2	A0A2L1CC83
FwdG	Formylmethanofuran dehydrogenase, subunit G	8.3	A0A2L1CCM5
FdhA	Formate dehydrogenase, subunit A	74.2	M9QXF8
FdhB	Formate dehydrogenase, subunit B	43.1	A0A7J9PDY4

Supplementary Table 4| Metal quantification of the Hdr-Vhu-Fwd super-assembly via ICP-MS.

Element	mol/mol protein	Expected
^{56}Fe	2712	2448
^{58}Ni	16	24
^{66}Zn	9	24
^{95}Mo	0.5	0
^{78}Se	205	108
^{184}W	32	12-16

Supplementary Table 5| Identified [NiFe] cofactor intermediates.

Intermediate	CN^-	CN^-	CO	redox state
Ni-SI	2100	2085	1918	Ni^{II}
Ni-L	2080	2062	1902	Ni^{I}
Ni-R	2102	2090	1938	Ni^{II}-μH

Supplementary Figures:

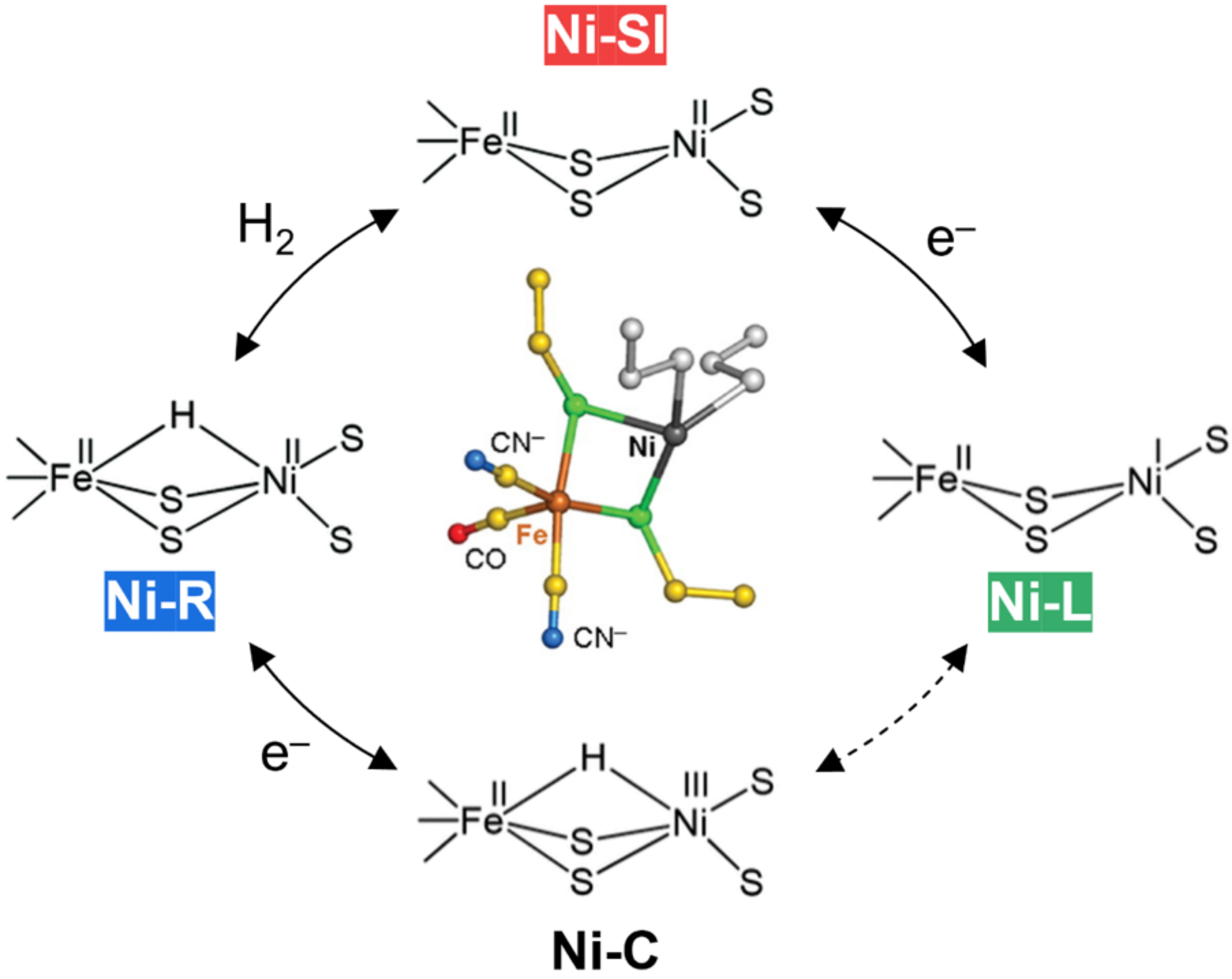

Supplementary Figure 1| Catalytic intermediates of [NiFe]-hydrogenase. Ni-SI represents the active-ready, oxidised state that stabilises an open coordination site between nickel and iron ion. H_2 reacts at the nickel ion and forms a Ni-Fe bridging hydride species in the “super-reduced” Ni-R state. Oxidisation and deprotonation of Ni-R by one electron forms the Ni-C state. Both Ni-R and Ni-C carry a bridging hydride. The release of this hydride is accompanied by a two-fold reduction of the cofactor in the Ni-L states and protonation of the protein fold. Oxidation and deprotonation restore Ni-SI and completes the cycle as shown in Extended Data Figure 9. See literature for further details on the catalytic mechanism[2]. The picture of the Ni-Fe cofactor was taken from[3].

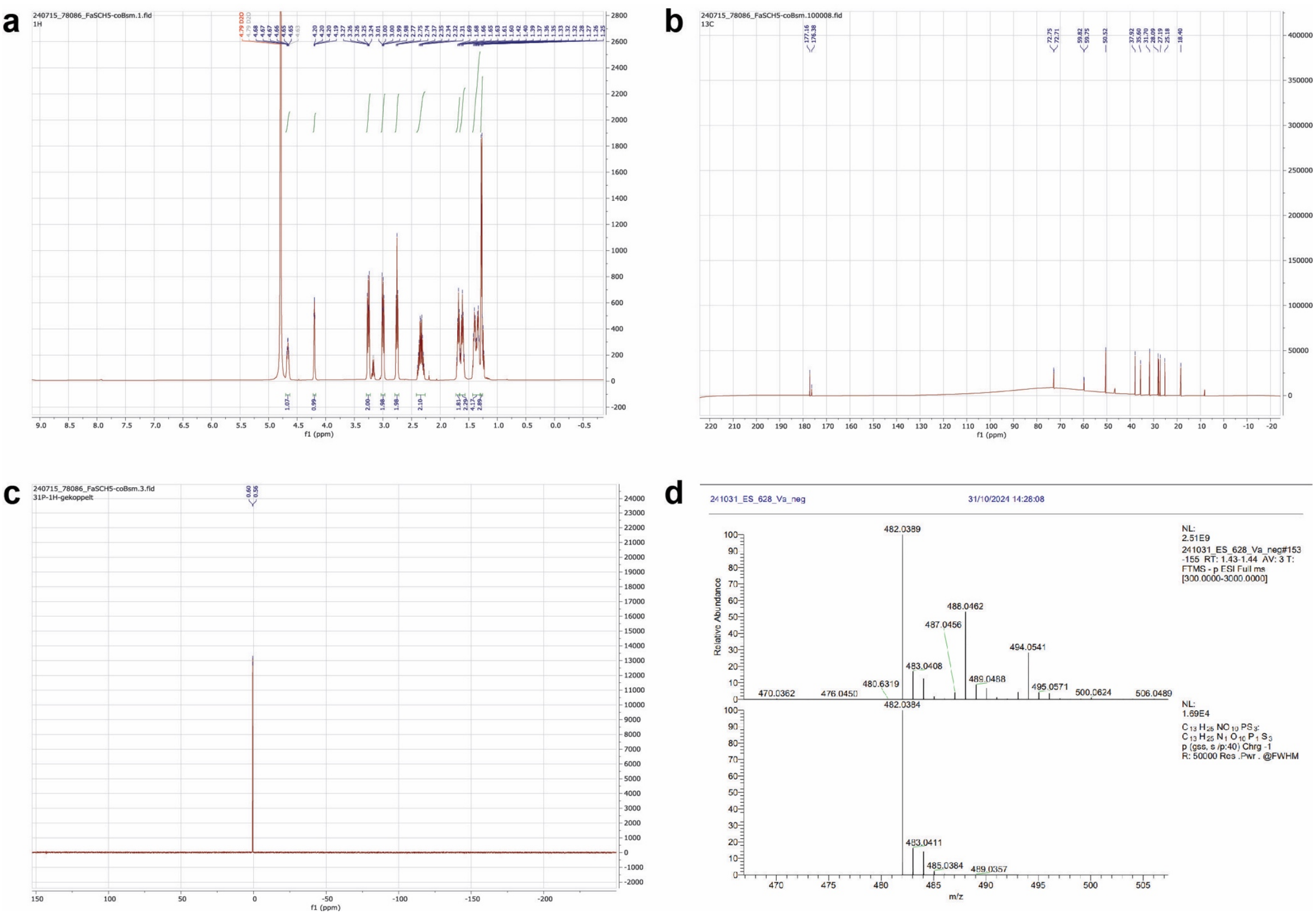

Supplementary Figure 2| NMR spectra of CoM-S-S-CoB heterodisulfide. a, ^{1}H NMR spectrum of CoM-S-S-CoB: ^{1}H NMR (500 MHz, D_2O) δ = 4.66 (dq, *J* = 6.9, 3.6 Hz, 1H, C<u>H</u>OPO_3^{2-}), 4.20 (dd, *J* = 3.5, 2.0 Hz, 1H, C<u>H</u>NHCO), 3.29 – 3.22 (m, 2H, C<u>H$_2$</u>SO_3^{-}), 3.03 – 2.96 (m, 2H, C<u>H$_2$</u>S), 2.75 (t, *J* = 7.3 Hz, 2H, C<u>H$_2$</u>S), 2.33 (tq, *J* = 14.3, 7.0 Hz, 2H, C<u>H$_2$</u>CO), 1.69 (q, *J* = 7.2 Hz, 2H, C<u>H$_2$</u>), 1.62 (td, *J* = 14.9, 13.3, 5.8 Hz, 2H, C<u>H$_2$</u>), 1.41 (d, *J* = 7.5 Hz, 1H, C<u>H$_2$</u>), 1.38 (d, *J* = 7.1 Hz, 1H, C<u>H$_2$</u>), 1.34 (d, *J* = 7.3 Hz, 2H, C<u>H$_2$</u>), 1.275 (d, *J* = 6.4 Hz, 3H, C<u>H$_3$</u>). **b,** ^{13}C NMR (126 MHz, D_2O) spectrum of CoM-S-S-CoB: δ =177.16 (COOH), 176.38 (CONH), 72.73 (d, *J* = 5.4 Hz, $CHOPO_3^{2-}$), 59.79 (d, *J* = 7.8 Hz, CHNH), 50.52 ($CH_2SO_3^{-}$), 37.92 (CH_2CO), 35.60 (CH_2S), 31.70 (CH_2S), 28.09 (CH_2), 27.19 (CH_2), 25.18 (CH_2), 18.40 (CH_3). **c,** ^{31}P NMR (202 MHz, Deuterium Oxide) spectrum: δ = 0.58 (d, *J* = 7.4 Hz). **d,** HRMS spectrum of CoM-S-S-CoB: HRMS (ESI) calc. for $C_{13}H_{25}NO_{10}PS_3$ [M - H]$^{-}$: 482.0389, Found: 482.0384

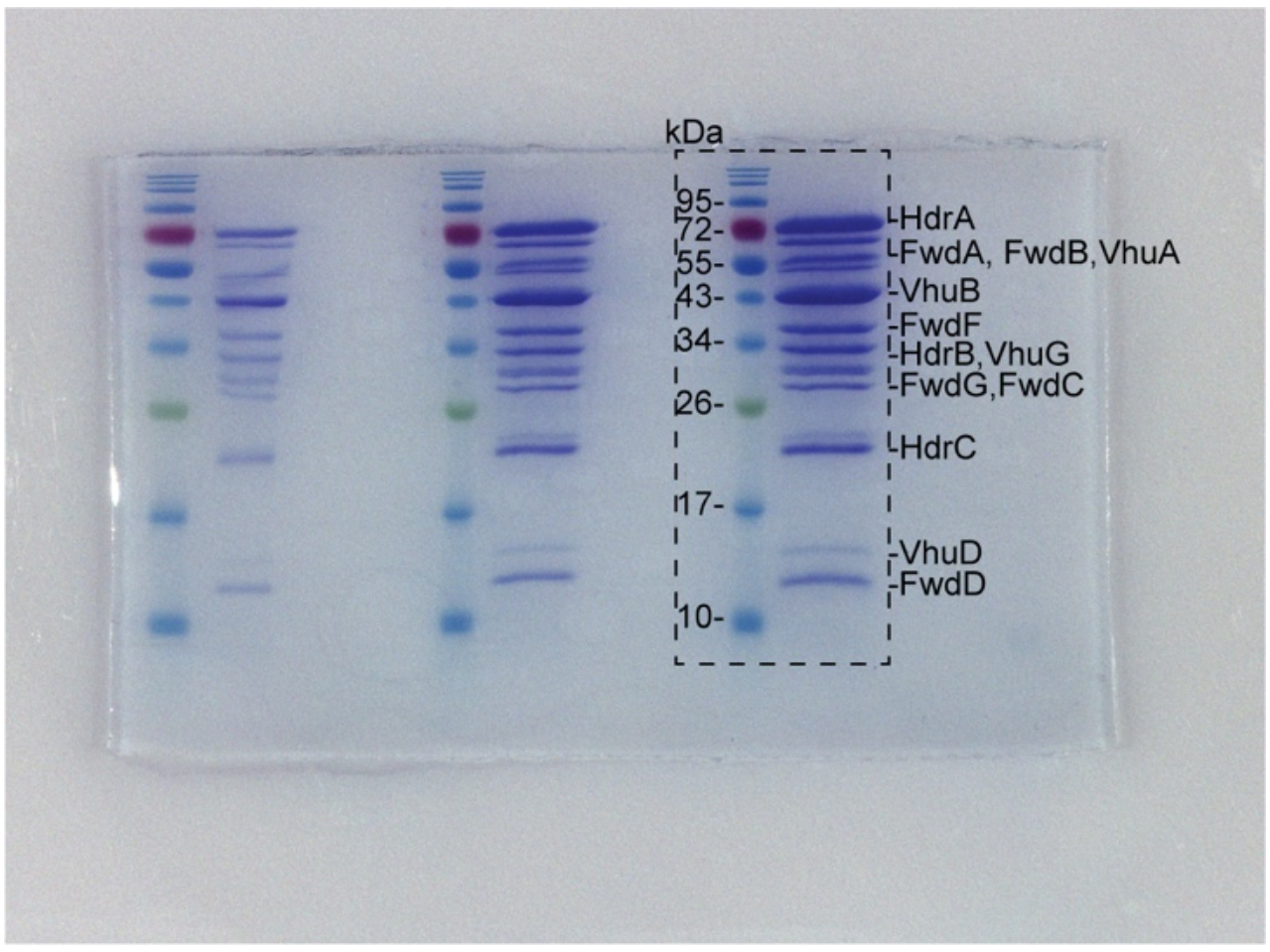

Supplementary Figure 3| Uncropped SDS-PAGE. Cropped region from Extended Data Fig. 1a is indicated with a dashed line.

Supplementary References:

1. Dodsworth, J. A. & Leigh, J. A. Regulation of nitrogenase by 2-oxoglutarate-reversible, direct binding of a PII-like nitrogen sensor protein to dinitrogenase. *Proceedings of the National Academy of Sciences* **103**, 9779–9784 (2006).
2. Shafaat, H. S., Rüdiger, O., Ogata, H. & Lubitz, W. [NiFe] hydrogenases: A common active site for hydrogen metabolism under diverse conditions. *Biochimica et Biophysica Acta (BBA) - Bioenergetics* **1827**, 986–1002 (2013).
3. Shima, S. *et al.* The Crystal Structure of [Fe]-Hydrogenase Reveals the Geometry of the Active Site. *Science* **321**, 572–575 (2008).
