# Supplementary material for: Architecture of the 8 MDa Hdr–Vhu–Fwd super-assembly in class I methanogens
Source: Nature. 2026 Jul 8;656(8129):1054–63. doi: 10.1038/s41586-026-10744-9 (PMC13518236; doi:10.1038/s41586-026-10744-9)
Supplement: Supplementary file 2 [file 41586_2026_10744_MOESM2_ESM.pdf]

nature portfolio

Corresponding author(s): Jan M. Schuller

Last updated by author(s): May 28, 2026

Reporting Summary

Nature Portfolio wishes to improve the reproducibility of the work that we publish. This form provides structure for consistency and transparency in reporting. For further information on Nature Portfolio policies, see our Editorial Policies and the Editorial Policy Checklist.

Statistics

For all statistical analyses, confirm that the following items are present in the figure legend, table legend, main text, or Methods section.

n/a	Confirmed	
☐	☒	The exact sample size (*n*) for each experimental group/condition, given as a discrete number and unit of measurement
☒	☐	A statement on whether measurements were taken from distinct samples or whether the same sample was measured repeatedly
☒	☐	The statistical test(s) used AND whether they are one- or two-sided<br>*Only common tests should be described solely by name; describe more complex techniques in the Methods section.*
☒	☐	A description of all covariates tested
☒	☐	A description of any assumptions or corrections, such as tests of normality and adjustment for multiple comparisons
☐	☒	A full description of the statistical parameters including central tendency (e.g. means) or other basic estimates (e.g. regression coefficient) AND variation (e.g. standard deviation) or associated estimates of uncertainty (e.g. confidence intervals)
☒	☐	For null hypothesis testing, the test statistic (e.g. *F*, *t*, *r*) with confidence intervals, effect sizes, degrees of freedom and *P* value noted<br>*Give P values as exact values whenever suitable.*
☒	☐	For Bayesian analysis, information on the choice of priors and Markov chain Monte Carlo settings
☒	☐	For hierarchical and complex designs, identification of the appropriate level for tests and full reporting of outcomes
☒	☐	Estimates of effect sizes (e.g. Cohen's *d*, Pearson's *r*), indicating how they were calculated

Our web collection on statistics for biologists contains articles on many of the points above.

Software and code

Policy information about availability of computer code

Data collection	SerialEM and EPU software were used to evaluate cryo-EM micrographs. SerialEM and PACEtomo were used to collect cryo-ET data. Infrared spectroscopy data was collected via Bruker's OPUS software.
Data analysis	Cryo electron microscopy data was analyzed with CryoSparc v4 software. For analysis and image generation of protein structure, UCSF Chimera v1.17.3, UCSF ChimeraX v1.8, and PyMOL v2.5.4 software was used. For model building and refinement, Coot v.0.9.8.95 and PHENIX software packages and tools were used. Prediction of structural models was performed using AlphaFold2 and 3 software and ModelAngelo v1.0. Quality assessment of the protein model was performed using PHENIX v. 1.19.2-4158. GSFSC analysis was performed using CryoSparc. Cryo-electron tomography data were processed using WarpTools and M v2.0.0dev31, RELION 5.0.0, AreTomo2 v1.0.0, pytom-match-pick v0.7.4, IsoNet2.0.0-beta and membrain-seg v0.0.8. ICP-MS analysis was performed using Qtegra. For nLC-MS/S Proteome Discoverer was used for data analysis. Multiple sequence alignment was performed with muscle v3.8.31_i86darwin64 and AliView v1.28. Infrared spectroscopy data was analyzed via Bruker's OPUS software and OriginLab's OriginPro 2026.

For manuscripts utilizing custom algorithms or software that are central to the research but not yet described in published literature, software must be made available to editors and reviewers. We strongly encourage code deposition in a community repository (e.g. GitHub). See the Nature Portfolio guidelines for submitting code & software for further information.

Data

Policy information about availability of data

All manuscripts must include a data availability statement. This statement should provide the following information, where applicable:

- Accession codes, unique identifiers, or web links for publicly available datasets
- A description of any restrictions on data availability
- For clinical datasets or third party data, please ensure that the statement adheres to our policy

Source data are provided with this paper. The Cryo-EM maps are available in the Electron Microscopy Data Bank (EMDB) with the accession codes EMD-54825 (composite map for the whole super-assembly) [https://www.ebi.ac.uk/emdb/EMD-54825] and EMD-55451 (composite map for the Hdr-Vhu-Fdh) [https://www.ebi.ac.uk/emdb/EMD-55451]. Local refined cryoEM maps are available under EMD-54996 (consensus map) [https://www.ebi.ac.uk/emdb/EMD-54996], EMD-54997 (VhuBI) [https://www.ebi.ac.uk/emdb/EMD-54997], EMD-54998 (VhuBII) [https://www.ebi.ac.uk/emdb/EMD-54998], EMD-54999 (Hdr(ABC)2) [https://www.ebi.ac.uk/emdb/EMD-54999], EMD-55009 (FwdF-Dimer) [https://www.ebi.ac.uk/emdb/EMD-55009], EMD-55010 (FwdI) [https://www.ebi.ac.uk/emdb/EMD-55010], EMD-55423 (FwdII) [https://www.ebi.ac.uk/emdb/EMD-55423], EMD-55424 (Vhu- Hydrogenase) [https://www.ebi.ac.uk/emdb/EMD-55424] and EMD-55426 (Fdh) [https://www.ebi.ac.uk/emdb/EMD-55426]. The atomic models are available in the Protein Data Bank (PDB) with the accession codes 9SFI for the minimal repeating unit of the Hdr-Vhu-Fwd complex [https://doi .org/10.2210/pdb9SFI/pdb] and under 9T1S for the Hdr-Vhu-Fdh complex [https://doi.org/10.2210/pdb9T1S/pdb]. The subtomogram averaging maps have been deposited to the Electron Microscopy Data Bank under accession numbers EMD-58202 (ribosome) [https://www.ebi.ac.uk/emdb/EMD-58202] and EMD-58201 (Hdr-Vhu-Fwd) [https://www.ebi.ac.uk/emdb/EMD-58201]. Structural Data that was used for comparison for Hdr-Vhu is available in the PDB under the code 5ODC and 5ODR. For Polyferredoxin VhuB comparison, MvhB from M. marburgenesis (Uniprot: P60232) and an AlphaFold3 prediction from M. formicicus (Uniprot: H1KZP8) was used. For the ferredoxin FwdF, the model from this study was compared with AlphaFold3 predictions and published models from different organisms: Uniprot IDs: A0A0F8UQE2 (M. mazei), A0A7K3YAW2 (M. archaeon), D9PU51 (M. marburgenesis), H1KX37 (M. formicicus), Q8TYJ0 (M. kandleri) and PDB: 7BKB (M. hungatei). For sequence analysis of FwdF, sequences can be accessed at NCBI: Methanosarcina mazei (WP_048038706.1), Methanococcus maripaludis (WP_181521371.1), Methanofervidicoccus abyssi (WP_131007595.1), Methanothermococcus okinawensis (DQVW01000100.1), Methanotorris formicicus Mc-S-70 (WP_007043806.1), Methanococcus jannaschii DSM 2661 (WP_010870679.1), Methanothermobacter wolfeii (WP_261599642.1), Methanopyrus kandleri DSM 6324 (WP_011018679.1), Methanocella conradii DSM 24694 (WP_014407032.1), Candidatus Methanolliviera hydrocarbonicum (RZN69131.1), Methanomicrobiales archaeon (WP_014867622.1), Methanothrix harundinacea 6Ac (WP_014586169.1) and Methanothrix thermoacetophila DSM 6194 (WP_011696724.1) All available archaeal genomes were downloaded from the Genome Taxonomy Database (GTDB) on 14th of April 2025 for sequence comparison. Strains and plasmids generated in this study are available from the corresponding authors upon request. Infrared spectroscopy data is provided in the source data file.

Research involving human participants, their data, or biological material

Policy information about studies with human participants or human data. See also policy information about sex, gender (identity/presentation), and sexual orientation and race, ethnicity and racism.

Reporting on sex and gender	Not applicable
Reporting on race, ethnicity, or other socially relevant groupings	Not applicable
Population characteristics	Not applicable
Recruitment	Not applicable
Ethics oversight	Not applicable

Note that full information on the approval of the study protocol must also be provided in the manuscript.

Field-specific reporting

Please select the one below that is the best fit for your research. If you are not sure, read the appropriate sections before making your selection.

☒ Life sciences ☐ Behavioural & social sciences ☐ Ecological, evolutionary & environmental sciences

For a reference copy of the document with all sections, see nature.com/documents/nr-reporting-summary-flat.pdf

Life sciences study design

All studies must disclose on these points even when the disclosure is negative.

Sample size	No statistical method was used to predetermine sample size. Sample sizes were selected based on established practice in the field, prior experience with the experimental methods, and routine implementation of these methods in the laboratory. The selected sample sizes were sufficient to ensure reproducibility and consistency across independent experiments. For cryo-EM, a data set with a total of >30,000 micrographs was acquired in order to have a sufficient number of particles, which resulted in a 3D reconstruction with a resolution of 2.7-3.4 Angstrom. The achieved resolution was enough to model the protein structure at the residue level. For cryo-ET, 150 tilt series were acquired, of which the 50 highest-quality tomograms were used for downstream subtomogram averaging.

Data exclusions	For cryo-EM, Hdr-Vhu-Fwd complex particles were picked for determination of the high resolution structure. Other particles were excluded from the analysis. For cryo-ET, analysis focused on the 50 highest-quality tomograms, selected as those in which ribosomes could be identified reliably and used as quasi-fiducials for downstream refinement. False-positive ribosome and Vhu-Hdr-Fwd picks were removed by 3D classification without alignment. No data were excluded from biochemical, biophysical or computational experiments.
Replication	Sample separation via polyacrylamide gels was performed at least in three independent biological repetitions, respectively. All replication attempts have been successful. For cryo-EM analysis of all >30K micrographs were analyzed. For cryo-ET, 150 tilt series were collected from at least an equal number of cells (typically 1-2 cells per tilt series), and subtomogram averaging was performed on particles identified across 50 tomograms. For ICP-MS and nLC-MS/MS analyses, one biological sample was used, as these measurements were performed as supportive analyses rather than for statistical comparison across conditions. These techniques are standard in the field and were used to complement and validate the structural findings obtained from the cryo-EM data, which are supported by multiple independent and consistent results.
Randomization	Resolution Determination for both single-particle cryo-EM and cryo-ET subtomogram averaging was carried out with two independent half sets according to the gold-standard FSC procedure implemented in CryoSPARC or RELION, respectively. For all other experiments, randomization was not applicable, since in microbiological assays all parameters are tightly controlled and therefore covariates are not relevant to our study.
Blinding	Blinding was not relevant to our study since no higher order species were used in this study. The study relied on objective biochemical and structural measurements, including standardized data collection and computational analysis workflows, which minimized the potential for observer bias.

Reporting for specific materials, systems and methods

We require information from authors about some types of materials, experimental systems and methods used in many studies. Here, indicate whether each material, system or method listed is relevant to your study. If you are not sure if a list item applies to your research, read the appropriate section before selecting a response.

Materials & experimental systems

n/a	Involved in the study
☒	☐ Antibodies
☒	☐ Eukaryotic cell lines
☒	☐ Palaeontology and archaeology
☒	☐ Animals and other organisms
☒	☐ Clinical data
☒	☐ Dual use research of concern
☒	☐ Plants

Methods

n/a	Involved in the study
☒	☐ ChIP-seq
☒	☐ Flow cytometry
☒	☐ MRI-based neuroimaging

Plants

Seed stocks	Not applicable
Novel plant genotypes	Not applicable
Authentication	Not applicable
