# Supplementary material for: Architecture of the 8 MDa Hdr–Vhu–Fwd super-assembly in class I methanogens
Source: Nature. 2026 Jul 8;656(8129):1054–63. doi: 10.1038/s41586-026-10744-9 (PMC13518236; doi:10.1038/s41586-026-10744-9)
Supplement: Supplementary file 3 [file 41586_2026_10744_MOESM3_ESM.pdf]

nature portfolio

Peer Review File

Architecture of the 8 MDa Hdr-Vhu-Fwd super-assembly in class I methanogens

Corresponding Author: Dr Jan Schuller

Any redactions in this file are there to maintain patient confidentiality, the confidentiality of unpublished data, or to remove third-party material.

This file contains all reviewer reports in order by version, followed by all author rebuttals in order by version.

Version 0:

Reviewer comments:

Referee #1

(Remarks to the Author)
This submission provides the first structural view of the Fwd-Hdr assembly in Class I hydrogenotrophic methanogens. While elements of the mechanistic discussion, particularly electron-bifurcation gating and cofactor-proximity arguments were already advanced by Watanabe et al in Science 2021 in the context of a Class II architecture, the present study reports a distinct Class I solution that differs substantially in stoichiometry, symmetry, overall topology, and polyferredoxin-mediated connectivity. Importantly, it also provides a structural basis for module swapping on the electron-input side.
The authors describe 8MDa, D3-symmetric complex composed of two hexameric HdrABC-Vhu rings bridged by three tetrameric Fwd units, resulting in a continuous, circular electron-transfer chain. A key new aspect is the proposed Class I specific assembly principle: the VhuB polyferredoxin occupies the central interface that in M. hungatei Class II is mediated by FmdF, thereby promoting higher-order oligomerization and rewiring how the modules are connected into a stable supramolecular architecture. The authors further identify a subpopulation of 18% in which Vhu is substituted by FdhAB at the Hdr scaffold, which they interpret as evidence for flexible integration of alternative redox inputs within the same overall framework.

Overall, the work appears substantially new relative to the 2021 Science study, primarily through demonstrating that Class I methanogens employ a different supramolecular design logic shaped by VhuB-mediated interfaces and that this framework can accommodate alternative electron-entry modules. The manuscript is well written and clearly illustrated. My comments are only technical to improve the paper and should be addressable with no problem.

1. The model statistics particularly the Ramachandran metrics can be better. Please refine the models to achieve validation metrics consistent with standards and report the improved statistics.
2. Please remove 'exceptionally large' from the abstract, the data speaks for itself.
3. Data availability: 'Data available upon request' is not appropriate. All the data should be available in the publication, and no request is needed.
4. Fig ED1, cartoons: it’s unclear what the cartoons are intended to convey. The lack of consistent scale is confusing, and the overall presentation does not meet the standard expected for a paper at this level. Please revise or remove these panels and replace them with clearer schematics.
5. Fig ED1, last panel: please add the scale bar size.
6. Fig ED2. Because the reconstructions are dominated by globular domains, the masks are difficult to see in print. Please adjust transparency and add a visual indicator to make the masking strategy obvious.
7. Fig ED8a: this figure needs work. The top-left panel is not optimal for illustrating the 120° movement. Consider overlaying maps with transparency. The cartoons could also be less spaghetti-like to make it easier for readers to relate the schematic to the maps.

Referee #2

(Remarks to the Author)

The manuscript reports a cryo-EM structure of the Hdr–Vhu–Fwd complex from Methanococcus maripaludis which couples H2 oxidation with CO_2 reduction dependent on flavin-based electron bifurcation. The structure of a variant is also reported in which the hydrogenase (Vhu) is substituted with formate dehydrogenase (Fdh). The high-quality structure of this exceptionally large complex is a tour-de-force for which the authors are commended.

The role and arrangement of multiple polyferredoxin (polyFd) subunits is prominently discussed as of central importance, as is substitution of Fdh to provide a thermodynamic advantage in the environment. However, the proposed role of the polyFd subunits (facilitating electron transfer between Hdr and Fwd) confirms the role reported for the cryo-EM structure of the Fdh-Hdr-Fwd complex from Methanospirillum hungatii (Science, 2021, v373, pp1151-56), even though the arrangement of the polyFd subunits is significantly different between the two species. The different arrangements reflect phylogenetic diversity which is not unexpected and fails to support a significant advance in mechanistic understanding. Finally, the assertion that both Vhu and Fdh electron input modules offers a thermodynamic advantage is not a reasonable conclusion considering H2 and HCOOH have similar redox potentials. Rather, the ability to switch between the two substrates offers an ecological advantage although this has already been well established in previous publications (i.e. PNAS, 2010, v107, pp11050-55). Thus, although the work has merit in reporting diversity in structures it falls short of a significant advance in mechanistic or ecological understanding.

Referee #3

(Remarks to the Author)
This is a well written and remarkable study that gives us insights into a number of aspects of biology and biological structure-function relationships. The implications will range from biophysics to evolution and ecology. It's a straightforward paper, and I have just a few comments and curiosity questions that arise that may be considered in revision by the authors.

Line 54 and 55. For the reduction potential's it's better to write both equivalently, for example either E0' for both or Em' for both. If the ferredoxin is correct as E0', then the disulfide value could be taken from the table in ref. 7

Line 132: why is it notable that FAD binding is accommodated by a Rossmann fold? Beta alpha beta and nucleotide binding is as common as bread and butter. Is there a deeper meaning here?

The section on flexibility and gating in relationship to electron flow and the bifurcation mechanism:
This is very important and interesting. I want to advocate a bit for some enhanced discussion and consideration of electron bifurcation based on the newly presented data. If the authors can consider this and add some to the paper it can make a bigger impact, though I understand this is primarily a structure support and the authors can choose as they wish.

The group in Marseille has written a lot on bifurcation e.g. 10.3389/fmicb.2018.01357 and it's worth to reflect on those papers. They describe the key role of proton transfer and pKa in modulating reduction potentials during electron bifurcation mechanism and also the geometry of redox cofactors in bifurcating proteins. Can the authors identify which protons might be key? If not just putting the question in the paper would point it out for further researchers.
Going further: How can we imaging this 4e- transfer process? With a 2e- process such as what happens in the Q cycle this has been studied and written about quite a bit. The splitting of the electrons can be rationalized for example with Marcus theory based on the rates of transfer to different acceptors. To consider the current case bit, Figure 3) panel C is not electronically balanced since 4 electrons are required. There are a number of ways this could happen, for example 4 electrons from two 2H2 molecules each singly have their reduction potentials modulated, leading to two, two electron reductions in the end.
Some inspiration for rationalizing a 4 electron transfer might come from the water oxidizing complex in photosynthesis: there a neighboring tyrosine may be acting as an electron reservoir as electrons come off the water. In the current structure there are ample places for electrons to go and it might take years to identify how they are trafficked and stored… I'm speculating in the above some but all this is said simply to point out some questions that might be raised in the paper such that readers could grasp better the implications of the current data and how to build off them.
The discussion of the thioredoxin like redox may be key here. It has been proposed that a disulfide bond in between the last Fes-cluster before the flavin actually is the (2-electron) donor to the flavin. Can the authors expand that a bit and discuss this so the reader can rationalize how R-Se-S-R specifically can be involved in the invoked scheme without having to do a deep dive into the thioredoxin literature? There may not be enough space in the main text but SI discussion could be good here.

The large genomic core of methanogens and the view of modularity: One of the interesting parts of the paper is the discussion of modularity and how some methanogens have different proteins which can affect their electron routing. This is important: for example we know that E.coli has a very large pan genome. It's one species in some taxonomy definitions but can vary hugely in its genome composition from strain to strain. In methanogens, actually the genome conservation is very high even across phyla. This poses some intriguing questions for the relationship between taxonomy and function: the variability identified by the authors is important for this taxonomically wide group of organisms and methanogens are an interesting case study of the taxonomy-physiology question.
citation: https://doi.org/10.1093/dnares/dsac048

Figure 3) panel D. Please add an arrow where the disulfide was added. I think it's obvious but good to mark that.

Extended data figure 11 is too low quality. Please improve it.

Question about the CO2, Formate, and Formyl MFR paper: the paper brings up some interesting questions about the role of formate.
We can think of reactions like this one below where the reduction takes place after carbamate formation.
CO_2 +MFR = CO_2MFR (carbamate) —> Formyl MFR
Bartoschek et al. Eur. J. Biochem. 267, FEBS 2000

But we can also think of CO_2 reduction to formate and then formation of Formyl MFR via formylmethanofuran dehydrogenase.

Can the authors discuss these two possibilities? Is it another aspect of “modularity” in that methanogens can potentially take multiple routes to FormylMFR?

~~~~~~~~~~~~~
Some time ago there were a series of fascinating papers about large molecular weight protein complexes in methanogens. These are different proteins than what the current paper is about, but it might be worthwhile referencing one or some: an important part of the current paper is not only the impact on the field of the determination of the described protein complex(s), but more broadly how biology is organized and how cells do seem to use very large protein complexes. This is important for our understandings of evolution and biophysics.

Hoppert and Mayer FEBS Volume 267, number 1, 33-37,
Braks et al. JOURNAL OF BACTIERIOLOGY, Dec. 1994, p. 7677-7687
F Mayer, M Rohde, M Salzmann, A Jussofie and G Gottschalk J. Bacteriol. 1988, 170(4):1438.

Version 1:

Reviewer comments:

Referee #1

(Remarks to the Author)
The authors have addressed my comments. They carried out additional processing, optimised the masking for local refinement, which improved the local map resolutions, improved the stereochemical quality of the models, and the presentation of the figures, as suggested.
In my view, the manuscript in its current form is ready for publication.
Congratulations.

Referee #2

(Remarks to the Author)
The authors have re-oriented the presentation to focus on organizational aspects rather than mechanistic and ecological that were previously emphasized as the more significant contribution. The new focus is primarily in response to reviewer #2 who is of the opinion that, although the organizational aspects are somewhat novel and the structure well supported, that aspect falls short of milestone advances. However, the revision also is in response to reviewers 1 and 3 that are of the strong opinion that the differences in organizational structure compared to the previous M. hungatii structure outweigh mechanistic and ecological considerations to which the authors have responded in the revision. The overwhelming support of reviewers 1 and 3 provides incentive for publication.

Referee #3

(Remarks to the Author)
The authors have provided a revised version of the manuscript with improvements in multiple aspects. The discussion added in the review phase is appreciated and will enhance the durability and readership of the paper. The paper provides a structural basis for multiple lines of continued research and begins to clarify some of the outstanding unknowns in the area.
~~~~~~~~~~~~~

Open Access This Peer Review File is licensed under a Creative Commons Attribution 4.0 International License, which permits use, sharing, adaptation, distribution and reproduction in any medium or format, as long as you give appropriate credit to the original author(s) and the source, provide a link to the Creative Commons license, and indicate if changes were made.
In cases where reviewers are anonymous, credit should be given to 'Anonymous Referee' and the source.
The images or other third party material in this Peer Review File are included in the article's Creative Commons license, unless indicated otherwise in a credit line to the material. If material is not included in the article's Creative Commons license and your intended use is not permitted by statutory regulation or exceeds the permitted use, you will need to obtain permission directly from the copyright holder.
To view a copy of this license, visit https://creativecommons.org/licenses/by/4.0/

Referee #1 (Remarks to the Author):

This submission provides the first structural view of the Fwd-Hdr assembly in Class I hydrogenotrophic methanogens. While elements of the mechanistic discussion, particularly electron-bifurcation gating and cofactor-proximity arguments were already advanced by Watanabe et al in Science 2021 in the context of a Class II architecture, the present study reports a distinct Class I solution that differs substantially in stoichiometry, symmetry, overall topology, and polyferredoxin-mediated connectivity. Importantly, it also provides a structural basis for module swapping on the electron-input side. The authors describe 8MDa, D3-symmetric complex composed of two hexameric HdrABC-Vhu rings bridged by three tetrameric Fwd units, resulting in a continuous, circular electron-transfer chain. A key new aspect is the proposed Class I specific assembly principle: the VhuB polyferredoxin occupies the central interface that in M. hungatei Class II is mediated by FmdF, thereby promoting higher-order oligomerization and rewiring how the modules are connected into a stable supramolecular architecture. The authors further identify a subpopulation of 18% in which Vhu is substituted by FdhAB at the Hdr scaffold, which they interpret as evidence for flexible integration of alternative redox inputs within the same overall framework.

Overall, the work appears substantially new relative to the 2021 Science study, primarily through demonstrating that Class I methanogens employ a different supramolecular design logic shaped by VhuB-mediated interfaces and that this framework can accommodate alternative electron-entry modules. The manuscript is well written and clearly illustrated. My comments are only technical to improve the paper and should be addressable with no problem.

1. The model statistics particularly the Ramachandran metrics can be better. Please refine the models to achieve validation metrics consistent with standards and report the improved statistics.

We thank the reviewer for these helpful and constructive comments. To further improve map and model quality, we implemented an additional processing step. In particular, recentring of the maps, combined with optimized masking for local refinement improved the resolution of all locally refined regions to 3.0–3.4 Å. As requested, we also refined the models further, resulting in improved stereochemical quality, with Ramachandran statistics exceeding 96% favored residues for all models. All relevant details and updated validation metrics are provided in Extended Data Table 4.

Additional informations in the manuscript:
Line 127: "This approach generated a series of local maps with resolutions ranging from 3.04 to 3.41 Å (Extended Data Fig. 2 and Extended Data Table 4), which enabled us to model the entire super-assembly (Fig. 1c,d and Extended Data Fig. 3)."

Addition to method section:
Line 458: "To additionally increase the resolution of the focused refined regions, all masks and symmetry expanded particles were recentred given the large size of the super-assembly and the resulting large box size. Recentring of the particles increased the resolution of the VhuB subunit to 3.30 Å and 3.19 Å, respectively. For the Hdr-stalk the local refinement resulted in a reconstructed map with a resolution of 3.04 Å, when the mask was created by excluding the N-terminal domain and the C-terminal ferredoxin domain. For Fwd, the complex was divided into three parts resulting in the FwdF-dimer (3.17 Å) and the outward- and inward facing FwdABCDG domains (3.15 Å and 3.18 Å). 3D analysis of the Vhu-hydrogenase arm revealed high degree of flexibility. Masked local refinement of the VhuADGU, including the N-terminal and C-terminal region of HdrA was carried out, followed by a 3D classification. Particles from suitable 3D classes were combined and further refined using local refinement. This refinement resolved a map with a resolution of 3.41 Å."

2. Please remove 'exceptionally large' from the abstract, the data speaks for itself.

Thank you for your advice. The term has been removed.

3. Data availability: 'Data available upon request' is not appropriate. All the data should be available in the publication, and no request is needed.

We thank the reviewer for raising this point. All relevant data have been deposited in the PDB and EMDB databases. The corresponding accession codes are provided in the Validation Table (Extended Data Table 4) as well as in the Data Availability section. All updated maps and models have been updated in the deposition and are accessible under the listed accession codes. In addition, the data have been submitted to Nature as part of the review process. We would like to clarify that the statement referred to the availability of strains upon request, rather than the deposited structural data; this was a miscommunication on our part. We apologize for any confusion caused. We will certainly provide all source data. However, we changed the Data availability section to avoid any misconception.

Data Availability section:
"Source data are provided with this paper.. The cryo-EM maps are available in the Electron Microscopy Data Bank (EMDB) with the accession codes EMD-54825 (composite map for the whole super-assembly) and EMD-55451 (composite map for the Hdr-Vhu-Fdh). Local refined cryoEM maps are available under EMD-54996 (consensus map), EMD-54997 (VhuB$_{I}$), EMD-54998 (VhuB$_{II}$), EMD-54999 (Hdr(ABC)$_{2}$), EMD-55009 (FwdF-Dimer), EMD-55010 (Fwd$_{I}$), EMD-55423 (Fwd$_{II}$), EMD-55424 (Vhu-Hydrogenas) and EMD-55426 (Fdh). The atomic models are available in the Protein Data Bank (PDB) with the accession codes 9SFI for the minimal repeating unit of the Hdr-Vhu-Fwd complex and under 9T1S for the Hdr-Vhu-Fdh complex. The subtomogram averaging maps have been deposited to the Electron Microscopy Data Bank under accession numbers EMD-XXXXX (ribosome) and EMD-XXXXX (Hdr-Vhu-Fwd)."

4. Fig ED1, cartoons: it's unclear what the cartoons are intended to convey. The lack of consistent scale is confusing, and the overall presentation does not meet the standard expected for a paper at this level. Please revise or remove these panels and replace them with clearer schematics.

We have removed this Figure and integrated the SDS-PAGE in the processing tree (now Extended Data Fig. 1).

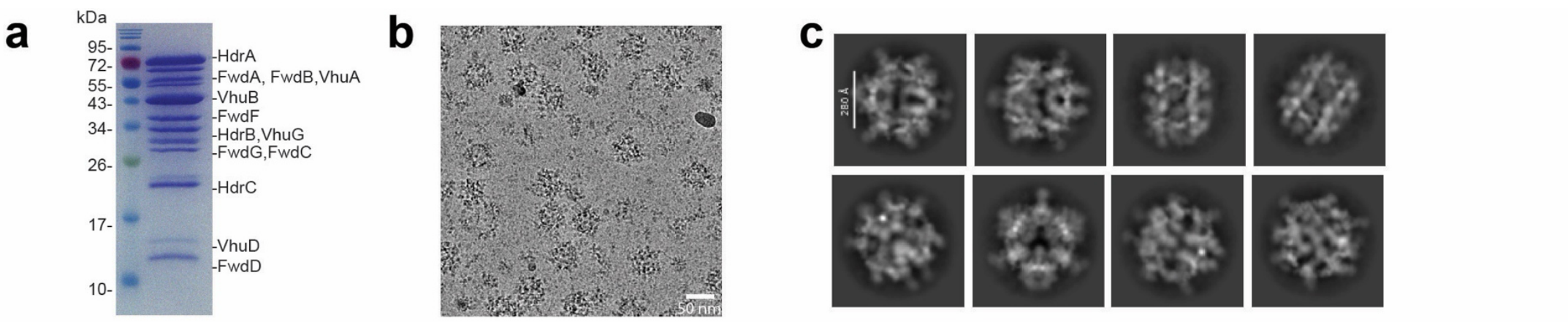

"Extended Data Fig. 1| Cryo EM processing workflow. a, *15% SDS-PAGE of the purified protein sample with the individual subunits after anaerobic affinity chromatography.* ***b,*** *Representative micrograph of the super-assembly showing ring shaped particles.* ***c,*** *2D-classes after Topaz-training revealing two stacked rings forming the assembly. Scale bar, 50 nm."*

5. Fig ED1, last panel: please add the scale bar size.

Corrected, thank you for pointing this out.

6. Fig ED2. Because the reconstructions are dominated by globular domains, the masks are difficult to see in print. Please adjust transparency and add a visual indicator to make the masking strategy obvious.

We thank the reviewer for this suggestion. We have updated the figure to improve clarity of the masking strategy by adding solid, brightly colored masks. In addition, arrows have been included as visual indicators to highlight the masked regions. We hope these changes make the masking approach clearer.

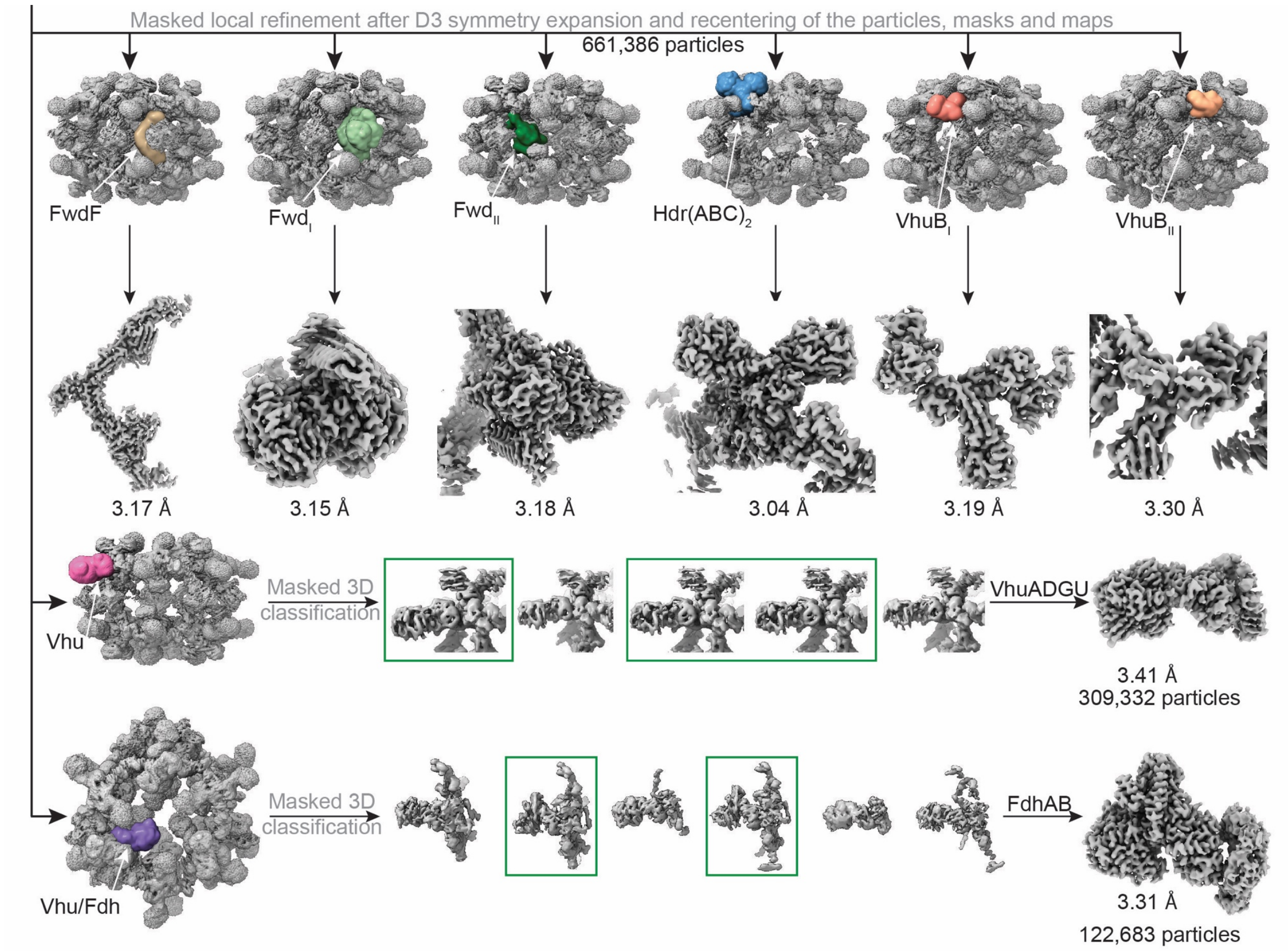

*“**Extended Data Figure 4d,** Processing tree showcasing the steps performed to determine the structure of the 8 MDa super-assembly. Moreover, local refinements were performed on the individual subunits and flexible regions to generate a composite map.”*

7. Fig ED8a: this figure needs work. The top-left panel is not optimal for illustrating the 120° movement. Consider overlaying maps with transparency. The cartoons could also be less spaghetti-like to make it easier for readers to relate the schematic to the maps.

Thank you for your suggestions to improve this figure. We have tried multiple options to visualize the movement of the hydrogenase arm. Even though 3D classification showed great movement, the number of particles for the one class (state 1) were less and could not lead to a high resolution map. Therefore, we decided to show state 2 with a resolution of 4.09 Å as a solid map (grey) and state 1 transparent (burgundy). We are now showing helices as tubes to enhance visual clarity and reduce structural clutter. We also decided to use a different color scheme to have a good contrast between the two states. In addition, we now added a schematic representation to differentiate the two states as clear as possible and to display the different subunits.
Updated Figure:

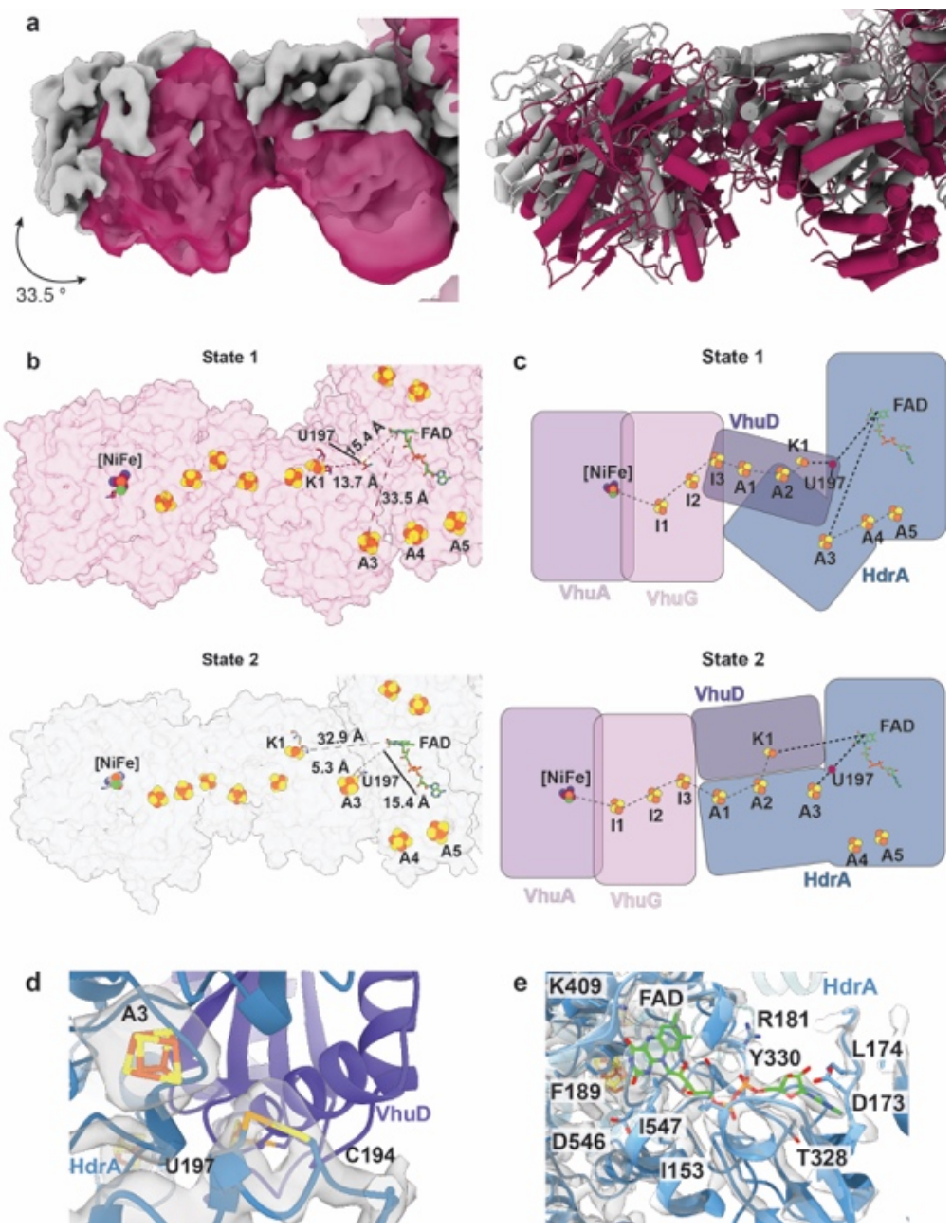

*“**Extended Data Fig. 9| Movement of the mobile hydrogenase arm. a,** Cryo-EM maps of the focussed refined electron input module after 3D classification analysis (left) and the corresponding, fitted models (right). **b,** Two conformational states enabling electron transport from the oxidation of H_2 at the [NiFe] cofactor to the FAD. State 1 showing the electron-transfer from the [NiFe] cofactor to the [2Fe-2S] cluster K1 and finally to the bifurcating FAD. Due to conformational changes, the FAD comes into closer proximity to the [4Fe-4S] cluster A3 in the N-terminal domain of HdrA, enabling the electron transfer to the ferredoxin. To bridge the long distance between the iron-sulphur clusters and the FAD, the selenocysteine SEC197 may be involved in enabling rapid electron transfer. **c,** Schematic representation of the two conformational states displayed in **b**, highlighting the movement of the individual subunits and the clusters involved in the electron transfer. **d,** Cryo-EM map at the SEC197 indicating a selenosulphur bond. **e,** Highlighting the interactions of FAD embedded in HdrA where flavin-based electron bifurcation takes place.”*

Referee #2 (Remarks to the Author):

The manuscript reports a cryo-EM structure of the Hdr–Vhu–Fwd complex from Methanococcus maripaludis which couples H2 oxidation with CO_2 reduction dependent on flavin-based electron bifurcation. The structure of a variant is also reported in which the hydrogenase (Vhu) is substituted with formate dehydrogenase (Fdh). The high-quality structure of this exceptionally large complex is a tour-de-force for which the authors are commended.

The role and arrangement of multiple polyferredoxin (polyFd) subunits is prominently discussed as of central importance, as is substitution of Fdh to provide a thermodynamic advantage in the environment. However, the proposed role of the polyFd subunits (facilitating electron transfer between Hdr and Fwd) confirms the role reported for the cryo-EM structure of the Fdh-Hdr-Fwd complex from Methanospirillum hungatii (Science, 2021, v373, pp1151-56), even though the arrangement of the polyFd subunits is significantly different between the two species. The different arrangements reflect phylogenetic diversity which is not unexpected and fails to support a significant advance in mechanistic understanding. Finally, the assertion that both Vhu and Fdh electron input modules offers a thermodynamic advantage is not a reasonable conclusion considering H2 and HCOOH have similar redox potentials. Rather, the ability to switch between the two substrates offers an ecological advantage although this has already been well established in previous publications (i.e. PNAS, 2010, v107, pp11050-55). Thus, although the work has merit in reporting diversity in structures it falls short of a significant advance in mechanistic or ecological understanding.

We thank the reviewer for the positive assessment of the structural work and for raising these important points. We agree that the general principle that polyferredoxins connect Hdr to Fwd/Fmd was established previously. The advance of the present work lies in revealing a distinct organization of this system in class I methanogens: a dodecameric, 8 MDa cellular super-assembly in which twelve electron-bifurcating modules are integrated into a continuous redox network. This architecture was previously unknown and cannot be inferred from the M. hungatei structure, which represents a 3 MDa hexameric assembly in class II methanogens.

We would like to emphasize that this study is focused on structural organization rather than on revisiting the underlying chemistry of flavin-based electron bifurcation or proposing a new ecological framework for methanogens. Instead, our objective was to resolve the integrative architecture of the class I Hdr-Vhu-Fwd system and determine how previously characterized biochemical modules build a functional cellular super-assembly. In this sense, the advance of the present work lies in bridging structural scales, from individual electron-bifurcating modules to a ~8 MDa assembly that organizes electron transfer and CO_2 fixation within a single cellular machine.

To address the reviewer's concern, we have revised the manuscript to more clearly emphasize the distinct architecture of the class I Hdr-Vhu-Fwd system and its differences from the previously reported class II Hdr-Fmd assembly. These clarifications have been incorporated in the revised Results and Discussion sections:

Updated title: "Architecture of the eight-megadalton Hdr-Vhu-Fwd super-assembly in hydrogenotrophic class I methanogens"

Line 78: "Whereas many genes are conserved among methanogenic archaea, distinct differences can be found between those that grow under different environmental conditions. Many class I methanogens exhibit hydrogenotrophic growth and thrive under high-temperature conditions, except for Methanococcus, which is reflected in their reduced genome sizes. Class II methanogens can grow on a broader substrate range and at lower temperatures, resulting in a larger genome and the ability to activate different metabolic pathways."

Line 331: "Previous work demonstrated direct coupling between Hdr and formyl-methanofuran dehydrogenase. However, our structure reveals a larger and fundamentally different architecture, which in situ cryo-ET confirms. At the core of this assembly lies a unique polyferredoxin-based architecture, in which class I-specific VhuB subunits mediate higher-order oligomerisation, while FwdF coordinates electron transfer between rings and towards the CO_2-reduction site. By directly linking all active sites, this super-assembly is able to confine all stages of CO_2 reduction within a single complex, effectively circumventing reliance on the cytoplasmic ferredoxin pool. The integrated Hdr-Vhu-Fwd system thereby enables ferredoxins to be allocated to energetically expensive reactions such as nitrogen fixation, cofactor biosynthesis, independently of catabolic CO_2 fixation."

We agree with the reviewer that H_2 and formate have similar midpoint potentials and that our previous wording regarding a "thermodynamic advantage" could be interpreted too strongly. We have therefore revised the manuscript to avoid this formulation. Instead, we now frame the coexistence of Vhu and Fdh modules as structural evidence that electron input module interchangeability is accommodated within the preassembled Hdr scaffold. With performing a 3D classification on the electron input modules attached to the Hdr-Dimer, our analysis shows that Fdh can occupy a defined position on the Hdr complex and bind only on one side while the other binding site is occupied by a hydrogenase. The simultaneous binding of two different electron input modules to a single Hdr-dimer has never been shown before. This points out the flexible and fast adaptation to changing conditions and rapid adaptation to keep the architecture and function of the super-assembly intact. This structural observation complements earlier physiological studies by illustrating how such metabolic flexibility is implemented within a single assembly. Therefore, the implementation of a formate dehydrogenase instead of a hydrogenase does not provide any thermodynamic advantage but the advantage of keeping the assembly intact even though there are environmental restraints such as hydrogen limitation.

Line 349: "Another particularly intriguing aspect of this architecture is its modularity, allowing alternative electron input modules to bind under H_2-limitation. Although previous studies have reported the association of different electron input modules, our data show for the first time that this occurs at the level of an individual HdrABC dimer, demonstrating that substrate interchangeability is structurally encoded within the preassembled complex. This modularity is also consistent with findings that under nickel limitation, Vhu can be exchanged with an alternative isoform of FdhA, also named as F_{420}-dependent electron donating proteins (Elp), that lacks a functional molybdenum-selenium active site but retains F_{420}-oxidising activity. Similarly, under selenium- limitation, the Vhu-hydrogenase can be replaced with its isoform Vhc, which lacks selenocysteine, yet remains catalytically functional.

The ecological implications of this distinction are substantial. In anaerobic environments, where H_2 and formate fluctuate independently, and nickel or selenium can be limiting, exchangeable electron input modules allow these methanogens to sustain CO_2 reduction and energy conservation through a rapid adjustment, without altering the bifurcation core. The ability to preserve a stable bifurcation and CO_2 reduction core while rapidly adjusting electron input modules is likely to be advantageous in fluctuating anaerobic habitats, and may offer class I methanogens a strategy to sustain methanogenesis under changing substrate and micronutrient availability. Such flexibility may be especially important in energetically marginal environments, including marine sediments and host-associated niches."

Finally, our study extends beyond previous structural work by demonstrating through cryo-electron tomography that the 8 MDa Hdr-Vhu-Fwd complex exists as a fully assembled complex within intact cells. In tomograms of vitrified M. maripaludis cells, we observe ring-shaped particles with dimensions matching those of the SPA reconstruction. Subtomogram averaging confirms the double-ring

architecture, and the single-particle model fits well into the tomographic density. These observations indicate that the organization revealed by cryo-EM reflects the native cellular architecture of this bioenergetic machinery and that the super-assembly exists as a discrete cytoplasmic complex without evidence for additional higher-order structures or membrane association. These results have been incorporated into manuscript.

Addition in the manuscript:

Line 30: "Cryo-electron tomography further supports that this super-assembly forms an intact structure within the cytoplasm of intact cells."

*Line 135: "**In situ cryo-electron tomography confirms the Hdr–Vhu–Fwd super-assembly in cells***

To determine whether the architecture observed by single-particle cryo-EM reflects the native organisation of the complex, we performed cryo-electron tomography of vitrified Methanococcus maripaludis cells (Fig. 2a). We applied a subtomogram averaging workflow using the abundant ribosomes as quasi-fiducial markers to refine tilt-series alignment and improve tomogram quality (Extended Data Fig. 4). The refined tomograms revealed large ring-shaped densities approximately 50 nm in diameter (Fig. 2c), consistent with the expected size and shape of the Hdr-Vhu-Fwd super-assembly.

We then used template matching to systematically pick these particles across the tomograms, and found that the complex is distributed throughout the cytoplasm (Fig. 2b), at an apparent concentration of 1.9 ± 0.3 µM (Fig. 2d and Extended Data Fig. 5). This cytoplasmic localisation contrasts with earlier immunogold labelling studies of methanogenic hydrogenases, which suggested a membrane-associated distribution[26]. To further characterise the in situ architecture of these assemblies, we performed subtomogram averaging of the ring-shaped particles, yielding an 11.1 Å reconstruction of the Hdr-Vhu-Fwd super-assembly (Fig. 2e, Extended Data Fig. 4f). The subtomogram average revealed a double-ring architecture that closely matched that of the purified complex determined by single-particle cryo-EM. Accordingly, docking of the single-particle model showed strong agreement with the in situ density, confirming that the dodecameric Hdr-Vhu-Fwd super-assembly forms within the cytoplasm of M. maripaludis. Together, these observations show that the architecture observed after purification faithfully reflects the physiological organisation of this electron-transfer machinery in vivo.

Line 331: "Previous work demonstrated direct coupling between Hdr and formyl-methanofuran dehydrogenase. However, our structure reveals a larger and fundamentally different architecture, which in situ cryo-ET confirms."

Referee #3 (Remarks to the Author):

This is a well written and remarkable study that gives us insights into a number of aspects of biology and biological structure-function relationships. The implications will range from biophysics to evolution and ecology. It's a straightforward paper, and I have just a few comments and curiosity questions that arise that may be considered in revision by the authors.

Line 54 and 55. For the reduction potential's it's better to write both equivalently, for example either E0' for both or Em' for both. If the ferredoxin is correct as E0', then the disulfide value could be taken from the table in ref. 7

We kindly appreciate the reviewer's comments. We have corrected both values to the reduction potential written as E^{0}' and used the value from ref. 7.

Following change was made to the manuscript:
Line 57: "These enzymes are powered with either a [NiFe]-hydrogenase (VhuADGU) or a formate dehydrogenase (FdhAB), connecting the exergonic reduction of CoM-S-S-CoB ($E^{0'}$= -281 mV) with the endergonic reduction of polyferredoxin subunits (ca. $E^{0'}$= -500 mV) through flavin-based electron bifurcation (FBEB)"

Line 132: why is it notable that FAD binding is accommodated by a Rossmann fold? Beta alpha beta and nucleotide binding is as common as bread and butter. Is there a deeper meaning here?

We agree that the presence of a Rossmann fold for FAD binding is not unexpected, given its well-established role in nucleotide cofactor recognition. Our intention in mentioning this feature was not to imply novelty, but rather to provide a complete and accurate structural description and to explicitly annotate the fold for clarity and consistency with standard nomenclature. We have revised the text to better reflect this point and to avoid overstating its significance.

We changed the wording in the manuscript to the following:

Line 161: "Each Hdr protomer harbors an electron-bifurcating FAD, located in the HdrA subunit, that binds in the typical FAD-binding pocket conserved in a Rossmann fold."

The section on flexibility and gating in relationship to electron flow and the bifurcation mechanism: This is very important and interesting. I want to advocate a bit for some enhanced discussion and consideration of electron bifurcation based on the newly presented data. If the authors can consider this and add some to the paper it can make a bigger impact, though I understand this is primarily a structure support and the authors can choose as they wish.

The group in Marseille has written a lot on bifurcation e.g. 10.3389/fmicb.2018.01357 and it's worth to reflect on those papers. They describe the key role of proton transfer and pKa in modulating reduction potentials during electron bifurcation mechanism and also the geometry of redox cofactors in bifurcating proteins. Can the authors identify which protons might be key? If not just putting the question in the paper would point it out for further researchers.

We thank the reviewer for these helpful comments. To address this point, we analyzed the environment of the non-cubane clusters in the HdrBC subunits and examined surrounding residues to identify potential proton transfer pathways in its vicinity. We have added a corresponding Extended Data Figure (Extended Data Fig. 10) and compared our observations with the crystal structure reported by Wagner et al. (2017).

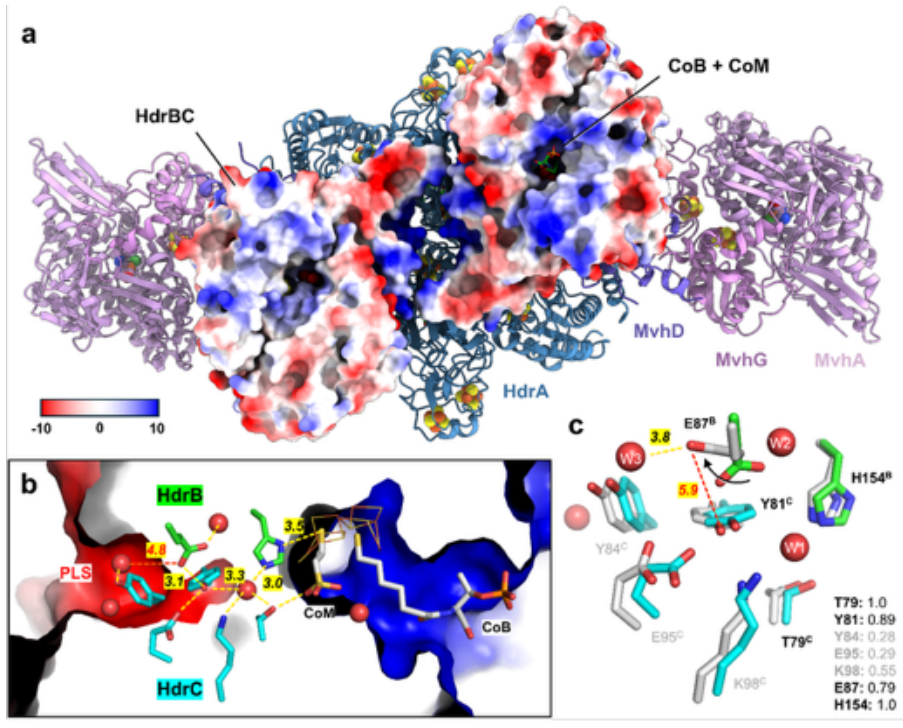

Addition in the Extended Data part of the manuscript:

Extended Data Fig. 10| Proton transfer pathway in HdrBC. a, *Overview of the MvhADG:HdrABC complex from Methanothermococcus thermolithotrophicus as crystalized in the presence of FAD and heterodisulfide (PDB ID 5ODR). MvhAG is equivalent to [NiFe]-hydrogenase VhuAUG and MvhD resembles VhuD (Fig. S5). Connected by the bifurcating HdrA subunit, the catalytic dimer HdrBC is depicted in surface representation where red and blue hint at preliminary negative and positive charge, respectively (calculated via the Adaptive Poisson-Boltzmann Solver). Note that only the right HdrBC dimer contains CoM and CoB in the active site pocket.* ***b****, This dimer reveals a negatively charged, water-filled proton loading site (PLS) and a positively charged heterodisulfide binding site. The latter is occupied by CoB and CoM as formed upon reduction of heterodisulfide and the non-cubane [4Fe-4S] clusters (shown as lines). Charged amino acid residues form a proton transfer pathway (PTP) between PLS and reactants, in particular histidine H154 and glutamate E87 of HdrB, tyrosine Y81 of HdrC as well as water molecule W1 at the interface of HdrB and HdrC. Residues T79, Y84, E94, and lysine K98 of HdrC may serve to stabilize further water moieties (W2, W3) and the CoM reactant (see panel c for labels). Starting in the PLS, we propose that protons proceed via W3, E87, Y81, W1, and H154 toward the thiol group of CoM. The distances range between 3.0–3.5 Å and justify the assumption of efficient proton transfer; however, at 4.8 Å E87 seems to be too far away from W3 in the PLS for any proton transfer reactivity.* ***c****, Comparison between key residues of HdrBC from M. thermolithotrophicus (green and cyan sticks) and M. maripaludis (white sticks). While the overall architecture of the PTP is conversed between both organisms, it is notable that the side chain of E87 is oriented toward a (potential) water molecule in M. maripaludis rather than Y81 as observed in M. thermolithotrophicus. This reduces the distance between W3 and E87 by 1 Å but nearly doubles the distance between E87 and Y81 from 3.1 Å to 5.9 Å. Please note that individual water molecules where not resolved in our cryo EM structure; therefore, the position of W1–W3 from PDB ID 5ODR was utilized in the analysis. We speculate that glutamate E87 serves as a gateway connecting PLS and PTP, the latter which delivers protons to the heterodisulfide binding site at the other end of the HdrBC dimer. Residues primarily involved in proton transfer are well conserved (identity 0.79–1.0, see table in the right corner).*

Going further: How can we imaging this 4e- transfer process? With a 2e- process such as what happens in the Q cycle this has been studied and written about quite a bit. The splitting of the electrons can be rationalized for example with Marcus theory based on the rates of transfer to different acceptors. To consider the current case bit, Figure 3) panel C is not electronically balanced since 4 electrons are required. There are a number of ways this could happen, for example 4 electrons from two 2H2 molecules each singly have their reduction potentials modulated, leading to two, two electron reductions in the end.
Some inspiration for rationalizing a 4 electron transfer might come from the water oxidizing complex in photosynthesis: there a neighboring tyrosine may be acting as an electron reservoir as electrons come off the water. In the current structure there are ample places for electrons to go and it might take years to identify how they are trafficked and stored… I'm speculating in the above some but all this is said simply to point out some questions that might be raised in the paper such that readers could grasp better the implications of the current data and how to build off them.
The discussion of the thioredoxin like redox may be key here. It has been proposed that a disulfide bondin between the last Fes-cluster before the flavin actually is the (2-electron) donor to the flavin. Can the authors expand that a bit and discuss this so the reader can rationalize how R-Se-S-R specifically can be involved in the invoked scheme without having to do a deep dive into the thioredoxin literature? There may not be enough space in the main text but SI discussion could be good here.

We thank the reviewer for these insightful and stimulating comments. We have considered in detail the mechanistic implications of the mentioned literature on our structure, in particular how sequential one-electron inputs could be coupled to the two-electron chemistry required for flavin reduction.

Based on the structural arrangement, we can outline a plausible working hypothesis. The large distance of ~20-30 Å between the K1 [2Fe–2S] cluster, the Cys-Sec motif, and FAD exclude direct thiol-disulfide exchange as well as efficient single-step electron transfer. Instead, this architecture suggests a distributed mechanism in which electron transfer proceeds through a series of electronically coupled steps, with the thiol/selenol module acting as a local redox-active intermediate.
In this model, electrons derived from the oxidation of hydrogen, transferred over multiple [4Fe-4S] clusters would arrive sequentially at the K1 [2Fe-2S] cluster, coordinated by three cysteines and two selenocysteines, and be transferred to a proximal redox-active center involving the Cys-Sec pair (U197 and C194). The presence of selenocysteine is particularly noteworthy, as its lower pK_a and higher nucleophilicity relative to cysteine may stabilize transient selenenylsulfide intermediates and facilitate rapid redox cycling. Such a Cys-Sec module could therefore act as a two-electron reservoir, transiently accumulating reducing equivalents from two successive one-electron transfer events. This would be analogous in principle to thioredoxin reductase-like systems, where a disulfide/dithiol motif couples one-electron transfer from a flavin to two-electron substrate reduction, although in those systems the cofactors are spatially co-localized.

In the present case, the spatial separation implies that electron storage and electron delivery are decoupled. The reduced dithiol/selenol state could then transfer a two-electron equivalent to the flavin, potentially via a transient thiolate-flavin interaction or via electronically coupled tunneling pathways involving intervening residues or cofactors. This would allow the system to reconcile sequential one-electron input from FeS clusters with the two-electron reduction chemistry of the bifurcating FAD.

At the same time, such a mechanism would provide a framework for multi-electron (ultimately four-electron) flow, in which reducing equivalents are accumulated and redistributed across the network of FeS clusters and redox-active residues. In this context, the thioredoxin-like motif could function not only as a redox buffer but also as a gating element, controlling when sufficient reducing equivalents are available to drive productive flavin chemistry and downstream bifurcation.
However, we emphasize that this model remains highly speculative. The current structures do not resolve the precise pathways of electron or proton transfer, nor do they establish the redox states of the proposed intermediates. In particular, the identity of proton donors/acceptors, the role of proton-coupled electron transfer, and the kinetics of electron redistribution within the network remain unclear. We are therefore cautious not to overinterpret the structural data. As noted above, even carefully formulated hypotheses can be taken as established mechanisms in the literature, and we prefer to avoid presenting such a model as definitive in the absence of direct experimental support.
For this reason, in the manuscript we have adopted a more cautious formulation, highlighting the thioredoxin-like motif as a potential redox-active module while explicitly framing its mechanistic role as an open question. We agree with the reviewer that this is an important area for future investigation, and that further spectroscopic, kinetic, and mutational studies will be required to test these hypotheses.

We have made the following changed to the manuscript:

Line 243: "The edge-to-edge distance between the A3 [4Fe–4S] cluster and the FAD cofactor (~33 Å; Extended Data Fig. 9b,c) exceeds the range of direct electron tunnelling, implying the presence of an intermediate relay. Inspection of the cryo-EM density revealed a distinct, continuous feature located between A3 and the flavin in state 2, coordinated by a cysteine and a selenocysteine residue U197 (Extended Data Fig. 9b,d). Given that HdrA contains sequence and structural features characteristic of

pyridine-nucleotide-dependent thioredoxin-like fold, it is plausible that this feature corresponds to a redox-active disulfide or selenylsulfide bridge that transiently mediates electron transfer between the two cofactors. In canonical thioredoxin reductases, electrons are transferred from the reduced flavin to an adjacent dithiol or selenolthiol centre, which then undergoes reversible thiol-disulfide or selenenylsulfide exchange reactions, coupled to conformational changes that gate access to downstream substrates. A related mechanism operates in DsbD-mediated electron transfer across the bacterial inner membrane, where electrons are propagated through a series of cysteine pairs via sequential disulfide exchange reactions, effectively forming a protein-based redox relay that bridges large spatial separations. By analogy, the Cys-Sec pair could function as a reversible thiol/selenol electron shuffle, transiently cycling between oxidised and reduced states to bridge the large distance between the buried FAD and distal Fe-S clusters[39]. The involvement of selenocysteine residues in this reaction is not strictly required, as they are not conserved across all methanogenic archaea. Nevertheless, their incorporation can be crucial for redox catalysis, owing to their enhanced nucleophilicity and reactivity relative to thiol groups. Substitution of cysteine with selenocysteine has the potential to enhance catalytic efficiency in electron transfer processes. At present, the precise role of the Cys–Sec pair, its coupling to FAD, and the potential contribution of selenocysteine to redox tuning remain unresolved and represents an open question that will require further structural, spectroscopic, and functional studies."

Additionally, we have updated the Extended Data Fig. 9 to visualize the electron transfer in the two conformational states:

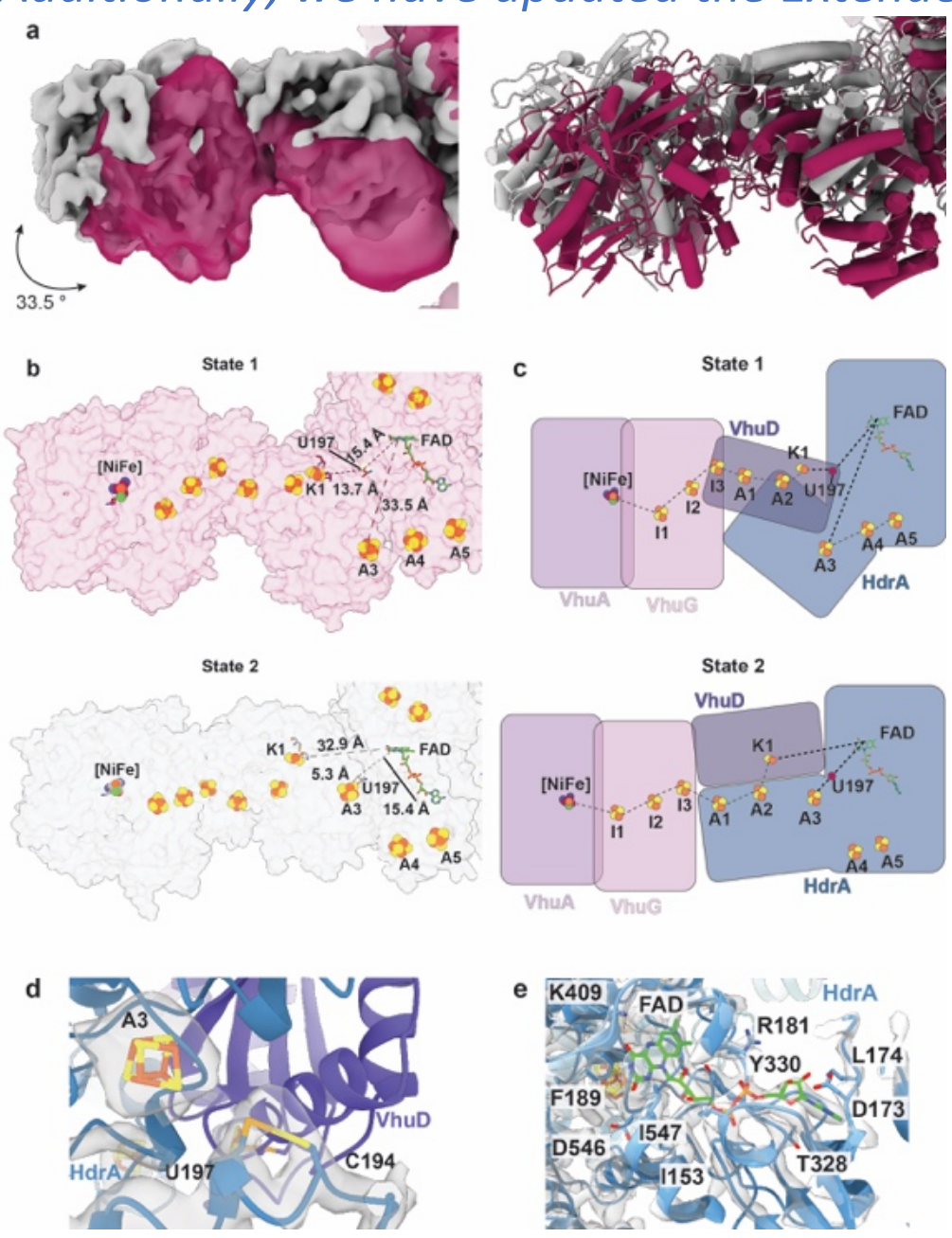

*"**Extended Data Fig. 9| Movement of the mobile hydrogenase arm. a,** Cryo-EM maps of the focussed refined electron input module after 3D classification analysis (left) and the corresponding, fitted models (right). **b,** Two conformational states enabling electron transport from the oxidation of H_2 at the [NiFe] cofactor to the FAD. State 1 showing the electron-transfer from the [NiFe] cofactor to the [2Fe-2S] cluster K1 and finally to the bifurcating FAD. Due to conformational changes, the FAD comes into closer proximity to the [4Fe-4S] cluster A3 in the N-terminal domain of HdrA, enabling the electron transfer to the ferredoxin. To bridge the long distance between the iron-sulphur clusters and the FAD, the selenocysteine SEC197 may be involved in enabling rapid electron transfer. **c,** Schematic representation of the two conformational states displayed in **b**, highlighting the movement of the individual subunits and the clusters involved in the electron transfer.*

***d,** Cryo-EM map at the SEC197 indicating a selenosulphur bond. **e,** Highlighting the interactions of FAD embedded in HdrA where flavin-based electron bifurcation takes place."*

The large genomic core of methanogens and the view of modularity: One of the interesting parts of the paper is the discussion of modularity and how some methanogens have different proteins which can affect their electron routing. This is important: for example we know that E.coli has a very large pan genome. It's one species in some taxonomy definitions but can vary hugely in its genome composition from strain to strain. In methanogens, actually the genome conservation is very high even across phyla. This poses some intriguing questions for the relationship between taxonomy and function: the variability identified by the authors is important for this taxonomically wide group of organisms and methanogens are an interesting case study of the taxonomy-physiology question. citation: https://doi.org/10.1093/dnares/dsac048

Class I and class II methanogens exhibit substantial differences, although many genes are conserved across these groups. Previous studies reported that growth temperature has an influence on the genome size as many class I methanogens are thermophilic. While Methanococcus species typically grow at moderate temperatures (e.g., 37 °C), many other class I methanogens are adapted to higher temperatures (see figure below):

REDACTED - Source article : https://
www.sciencedirect.com/science/article/pii/
S2590262822000478#f0010 (Figure 2)

Figure from Li et. al., 2023 showing the phylogeny of methanogens and their temperature optima

However, genome size is not determined by temperature alone. Environmental conditions and substrate availability also play a major role. For example, Methanosarcina acetivorans possesses the largest known archaeal genome, which is consistent with its ability to utilize a wide range of substrates and thrive under diverse environmental conditions.

To further clarify the differences between class I and class II methanogens, we have added the following text to the introduction section of the manuscript:

Line 78: "Whereas many genes are conserved among methanogenic archaea, distinct differences can be found between that grow under different environmental conditions. Many class I methanogens exhibit hydrogenotrophic growth and thrive under high-temperature conditions, except for Methanococcus, which is reflected in their reduced genome sizes. Class II methanogens can grow on a broader substrate range and at lower temperatures, resulting in a larger genome and the ability to activate different metabolic pathways."

In the discussion we have added the following section:

Line 349: "Another particularly intriguing aspect of this architecture is its modularity, allowing alternative electron input modules to bind under H_2-limitation. Although previous studies have reported the association of different electron input modules, our data show for the first time that this occurs at

the level of an individual HdrABC dimer, demonstrating that substrate interchangeability is structurally encoded within the preassembled complex. This modularity is also consistent with findings that under nickel limitation, Vhu can be exchanged with an alternative isoform of FdhA, also named as F_{420}-dependent electron donating proteins (Elp), that lacks a functional molybdenum-selenium active site but retains F_{420}-oxidising activity. Similarly, under selenium- limitation, the Vhu-hydrogenase can be replaced with its isoform Vhc, which lacks selenocysteine, yet remains catalytically functional.

The ecological implications of this distinction are substantial. In anaerobic environments, where H_2 and formate fluctuate independently, and nickel or selenium can be limiting, exchangeable electron input modules allow these methanogens to sustain CO_2 reduction and energy conservation through a rapid adjustment, without altering the bifurcation core. The ability to preserve a stable bifurcation and CO_2 reduction core while rapidly adjusting electron input modules is likely to be advantageous in fluctuating anaerobic habitats, and may offer class I methanogens a strategy to sustain methanogenesis under changing substrate and micronutrient availability. Such flexibility may be especially important in energetically marginal environments, including marine sediments and host-associated niches."

Figure 3) panel D. Please add an arrow where the disulfide was added. I think it's obvious but good to mark that.

For the infrared experiments, samples were incubated without any substrates (left graph) and with the addition of heterodisulfide (right graph). Therefore, no heterodisulfide was added during the measurement but incubated before the infrared measurement. In our experimental setup, the gas-atmosphere could be controlled and therefore, we exchanged nitrogen gas (N_2) for hydrogen (H_2), which led to an activation of the hydrogenase. We could also see reversible effects once the hydrogen was replaced with nitrogen again.

We expanded the figure description to make it more clear for the reader:

*Line 704: "**Fig. 3d**. Redox controlled infrared measurement performed in a gas-controlled chamber. Hydrogenase was activated by switching from N_2 to H_2, followed by removal of H_2. CO_2 was supplied under both conditions as a low-potential electron acceptor in Fwd, while heterodisulfide (HDS) was added (right) as a high-potential electron acceptor in HdrB."*

Extended data figure 11 is too low quality. Please improve it.

We have made efforts to improve the quality of Extended Data Figure 11. However, due to file size limitations and the compression required for submission, a slight reduction in figure quality is unavoidable at this stage. To ensure optimal presentation, we will provide the original high-resolution and vector-format files separately to enable inclusion of a high-quality final version.

Question about the CO2, Formate, and Formyl MFR paper: the paper brings up some interesting questions about the role of formate.
We can think of reactions like this one below where the reduction takes place after carbamate formation.
CO2 +MFR = CO2MFR (carbamate) —> Formyl MFR
Bartoschek et al. Eur. J. Biochem. 267, FEBS 2000

But we can also think of CO2 reduction to formate and then formation of Formyl MFR via formylmethanofuran dehydrogenase.

Can the authors discuss these two possibilities? Is it another aspect of "modularity" in that methanogens can potentially take multiple routes to FormylMFR?

The structural work by Wagner et al. (2017) on the class I formyl-methanofuran dehydrogenase provides important insight into this question. The authors describe a hydrophilic tunnel connecting the CO_2 reduction site and the formyl-MFR formation site, which enables the transient storage and channeling of formate between these two active sites. This architecture effectively separates the reduction of CO_2 from the subsequent transfer to methanofuran, while ensuring that the generated formate is specifically directed toward formyl-MFR formation. This mechanistic arrangement also implies that, even when formate dehydrogenase serves as an electron input module, formate primarily functions as an intermediate carrier of reducing equivalents within the enzyme complex, rather than as a freely diffusing metabolite that is re-assimilated.

While carbamate formation from CO_2 and MFR cannot be excluded and may occur spontaneously in solution, its direct conversion to formyl-MFR would require a multi-electron reduction of a carboxylate group. Such a reaction is not compatible with the known catalytic capabilities of formyl-methanofuran dehydrogenase, making this pathway highly unlikely.

In this context, the modularity of the electron input system is best understood as a means to ensure efficient and continuous electron supply for CO_2 reduction. Within the Hdr super-assembly, multiple electron transfer pathways can be routed to different active sites, supporting catalytic flexibility while maintaining a defined sequence of chemical transformations.

~~~~~~~~~~~~~

Some time ago there were a series of fascinating papers about large molecular weight protein complexes in methanogens. These are different proteins than what the current paper is about, but it might be worthwhile referencing one or some: an important part of the current paper is not only the impact on the field of the determination of the described protein complex(s), but more broadly how biology is organized and how cells do seem to use very large protein complexes. This is important for our understandings of evolution and biophysics.

Hoppert and Mayer FEBS Volume 267, number 1, 33-37,
Braks et al. JOURNAL OF BACTIERIOLOGY, Dec. 1994, p. 7677-7687
F Mayer, M Rohde, M Salzmann, A Jussofie and G Gottschalk J. Bacteriol. 1988, 170(4):1438.

We thank the reviewer for highlighting these important early studies. The cited work indeed provided pioneering evidence for the existence of large macromolecular assemblies in methanogens and has been highly inspiring for our study. In particular, the work by Braks et al. (1994) is of direct relevance, as it investigated the non-F_{420}-reducing hydrogenase, which is also a central component of the super-assembly described here.

In that study, immunogold labeling suggested a predominantly membrane-associated localization of the enzyme. However, using cryogenic electron tomography, we observe that the non-F_{420}-reducing hydrogenase (Vhu), when part of the Hdr super-assembly, is distributed throughout the cytoplasm. We therefore interpret the earlier membrane-associated signal as likely influenced by methodological limitations of immunogold labeling, including its limited spatial resolution and a known tendency for particle accumulation at membrane surfaces.

We appreciate the early finding of these very large complexes and therefore included the citations in the manuscript:

Line 341: "Only a limited number of biological systems reach comparable structural scales, and notably many belong to energy-converting electron transfer machineries, including respiratory chain complexes, photosynthetic reaction centre-antenna assemblies, filamentous nanowires such as the
~~~~~~~~~~~~~

hydrogen-dependent CO_2 reductase (HDCR) complex, and other large assemblies described in methanogens"

Additionally, we included the early findings of the non-F_{420}-reducing hydrogenase in the manuscript as following:

Line 143: "We then used template matching to systematically pick these particles across the tomograms, and found that the complex is distributed throughout the cytoplasm (Fig. 2b), at an apparent concentration of 1.9 ± 0.3 μM (Fig. 2d and Extended Data Fig. 5). This cytoplasmic localisation contrasts with earlier immunogold labelling studies of non-F_{420}-reducing hydrogenases, which suggested a membrane-associated distribution"

Reference:

Li, J., Akinyemi, T.S., Shao, N., Chen, C., Dong, X., Liu, Y. and Whitman, W.B., 2023. Genetic and metabolic engineering of Methanococcus spp. Current Research in Biotechnology, 5, p.100115.

Referees' comments:

Referee #1 (Remarks to the Author):

The authors have addressed my comments. They carried out additional processing, optimised the masking for local refinement, which improved the local map resolutions, improved the stereochemical quality of the models, and the presentation of the figures, as suggested.
In my view, the manuscript in its current form is ready for publication.
Congratulations.

Thank you for your positive evaluation and helpful comments. We appreciate your time and are glad the revisions addressed your concerns. We are grateful for your recommendation for publication.

Referee #2 (Remarks to the Author):

The authors have re-oriented the presentation to focus on organizational aspects rather than mechanistic and ecological that were previously emphasized as the more significant contribution. The new focus is primarily in response to reviewer #2 who is of the opinion that, although the organizational aspects are somewhat novel and the structure well supported, that aspect falls short of milestone advances. However, the revision also is in response to reviewers 1 and 3 that are of the strong opinion that the differences in organizational structure compared to the previous M. hungatii structure outweigh mechanistic and ecological considerations to which the authors have responded in the revision. The overwhelming support of reviewers 1 and 3 provides incentive for publication.

Thank you for your careful evaluation and constructive summary of the reviews. We appreciate your guidance on the revised focus and are grateful for your recommendation for publication.

Referee #3 (Remarks to the Author):

The authors have provided a revised version of the manuscript with improvements in multiple aspects. The discussion added in the review phase is appreciated and will enhance the durability and readership of the paper. The paper provides a structural basis for multiple lines of continued research and begins to clarify some of the outstanding unknowns in the area.

Thank you very much for your positive assessment. We appreciate your recognition of the revisions and are glad the added discussion improves the manuscript. We are also grateful for your encouraging comments on its broader impact and significance.
